# *De novo* design of constrained and sequence-independent peptide scaffolds with topologically-formidable disulfide connectivities†
†Electronic supplementary information (ESI) available: Detailed experimental section, characterization of peptides, tryptic digestion HPLC and MS analysis of disulfide pairing in peptides (Fig. S1–S50). See DOI: 10.1039/c7sc03956e


**DOI:** 10.1039/c7sc03956e

**Published:** 2017-11-20

**Authors:** Yiwu Zheng, Xiaoting Meng, Yaqi Wu, Yibing Zhao, Chuanliu Wu

**Affiliations:** a The MOE Key Laboratory of Spectrochemical Analysis and Instrumentation , State Key Laboratory of Physical Chemistry of Solid Surfaces , Department of Chemistry , College of Chemistry and Chemical Engineering , Xiamen University , Xiamen , 361005 , P. R. China . Email: chlwu@xmu.edu.cn

## Abstract

Disulfide-rich peptides are interesting scaffolds for drug design and discovery. However, peptide scaffolds constrained by disulfide bonds, either naturally occurring or computationally designed, have been suffering from the elusive (oxidative) folding behavior complying with Anfinsen's dogma, which strongly restricts their applicability in bioactive peptide design and discovery; because when primary peptide sequences are extensively manipulated, their disulfide connectivities might become scrambled. Here we present the design of cysteine/penicillamine (C/Pen)-mixed peptide frameworks that are capable of folding into specific regioisomers without dependence on primary amino acid sequences. Even certain folds that are considered to be topologically formidable can be generated in high yields. Currently, almost all disulfide-rich peptide scaffolds are vitally correlated to primary amino acid sequences, but ours are exceptional. These scaffolds should be of particular interest for further designing constrained peptides with new structures and functions, and more importantly, the ultimately designed peptides would not suffer from general oxidative folding problems.

## Abstract 2

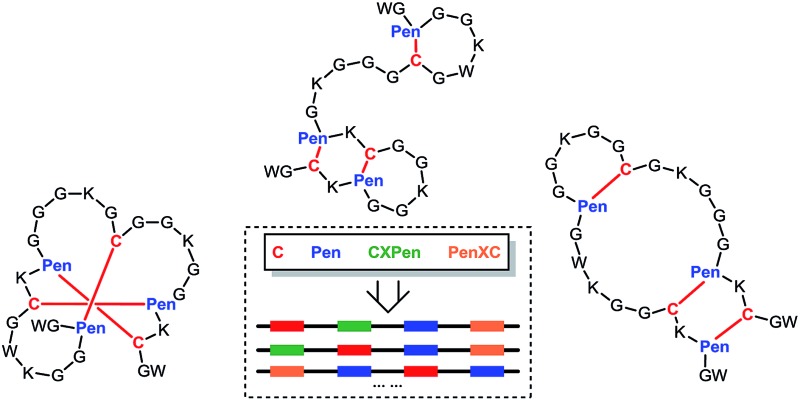
We developed a novel approach for designing a class of constrained and sequence-independent peptide scaffolds with three or four disulfide bonds. Even specific peptide folds that have been considered to be topologically formidable can be *de novo* created and synthesized in high yields.

## Introduction

Constrained peptides—lying between larger biologics and small molecules in size, and presumably combining the advantages of both—have been a treasured chemical space for drug developments.^1^,^2^ Among the diverse pharmacologically active peptides constrained with covalent crosslinks, naturally occurring disulfide-rich peptides, including plant-derived cyclotides, antimicrobial defensins, and conotoxins from venoms of predatory marine snails, are most extensively explored.^2^–^5^ Novel bioactive disulfide-constrained peptides can be routinely developed by re-engineering these naturally occurring scaffolds using loop grafting and high-throughput sequence selection.^6^,^7^ However, there are limits for the structural variety of naturally occurring constrained peptides, which thus restricts the design of inhibitors to certain targets with a surface topology congenitally complementary to the constrained peptide scaffolds.^8^ A strategy to break through this limitation comes from the recent advances in *de novo* protein design, based on which disulfide-constrained peptide scaffolds with new structures can be created without reference to known structures.^8^,^9^ Moreover, the computational methods enable the design of constrained peptide structures with non-canonical backbones through incorporating unnatural amino acids. However, for both the naturally occurring and *de novo* designed disulfide-rich peptides, the correct pairing of disulfide bonds and the formation of native structure is primarily driven by the amino acid sequences according to Anfinsen's dogma.^2^,^4^,^10^ Thus, existing peptide scaffolds are, in principle, not compatible with the design of sequence-randomized libraries, where the sequences are extensively manipulated and the disulfide pairing may become scrambled. Therefore, it is certainly worth developing constrained and sequence-independent peptide scaffolds with an inherently high propensity of forming correct disulfide connectivities. Such scaffolds should be ideal topologically-fixed backbones amenable to sequence randomization, and if combining with the computational design,^8^ disulfide-constrained peptides with new structures and functions would be created *de novo*.

**Fig. 1:**
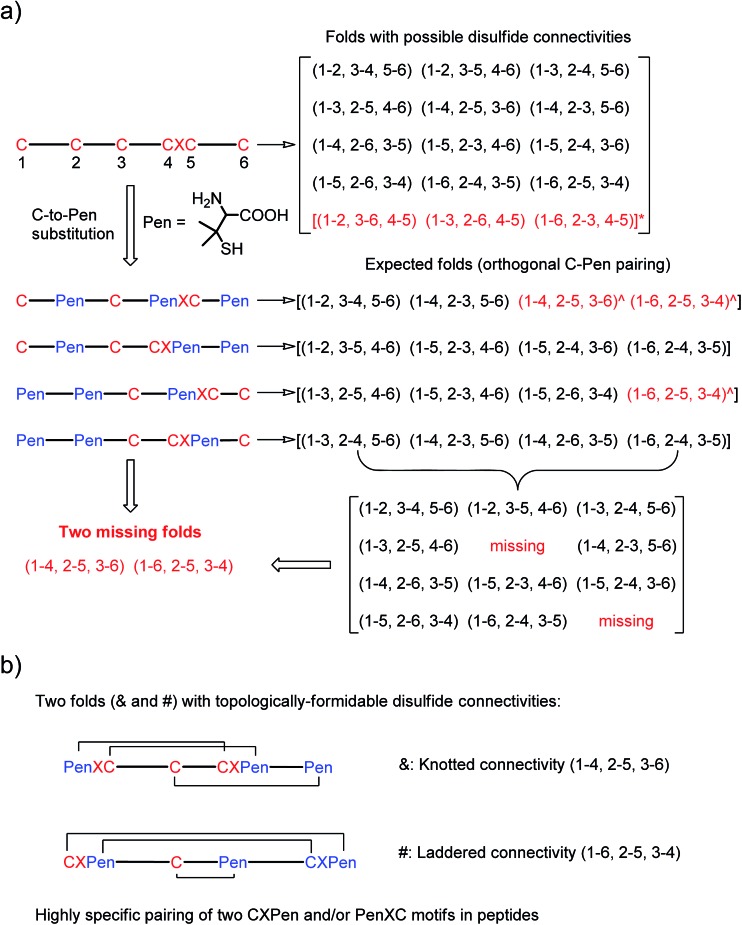
(a) All possible or expected folds that might form after the oxidative folding of peptides with specific cysteine-rich or C/Pen-mixed frameworks. * represents folds with an intra-CXC motif disulfide connectivity (4–5) that are strongly disfavored due to high ring-tension of the CXC motifs;^11^ ^ represents folds with a knotted or laddered disulfide connectivity that are not observed after oxidative folding (*i.e.*, two missing folds). (b) The two missing folds (& and #) can be *de novo* created using C/Pen-mixed frameworks containing two CXPen and/or PenXC motifs. Note that: the symbols & and # represent visually the knotted and laddered fold, respectively.

Previous reports have demonstrated the unique effect of CXC (cysteine-*any*-cysteine) motifs and the incorporation of penicillamine (Pen) on the oxidative folding of either natural or synthetic peptides.^11^–^13^ However, the essentials of directing the folding of peptide into specific isomers with up to three disulfide bonds have not yet been grasped. We hypothesized that the interplay between CXC motifs and Pen residues in peptides can be rationally manipulated to direct the folding of C/Pen-rich peptides into specific folds with three or four disulfide bonds without dependence on amino acid sequences. Thus, in this work we describe our effort of *de novo* designing C/Pen-mixed peptide frameworks with one or two CXPen/PenXC motifs that can precisely fold into a subset of specific folds fully covering all possible isomers oxidatively folding from typical cysteine-rich peptides (Fig. 1). All designs can generate as low as four specific isomers, and a subset of them are able to fold into one or two specific ones. Even certain peptide scaffolds that have been considered to be formidable in topology can be generated in high yields (*e.g.*, folds with three disulfide bonds in a knotted (&) and laddered (#) arrangement). These topologically-formidable scaffolds are of particular interest for further designing constrained peptides with new structures and functions, because they are hyper-constrained and new peptides created based on them should be exceptionally resistant to proteolysis and chemical denaturation. This work demonstrate the power of combining two conceptually-different orthogonal disulfide pairing technologies for *de novo* designing constrained and sequence-independent peptide scaffolds without recourses to naturally occurring peptide sequences and computational methods.

## Results and discussion

**Fig. 2:**
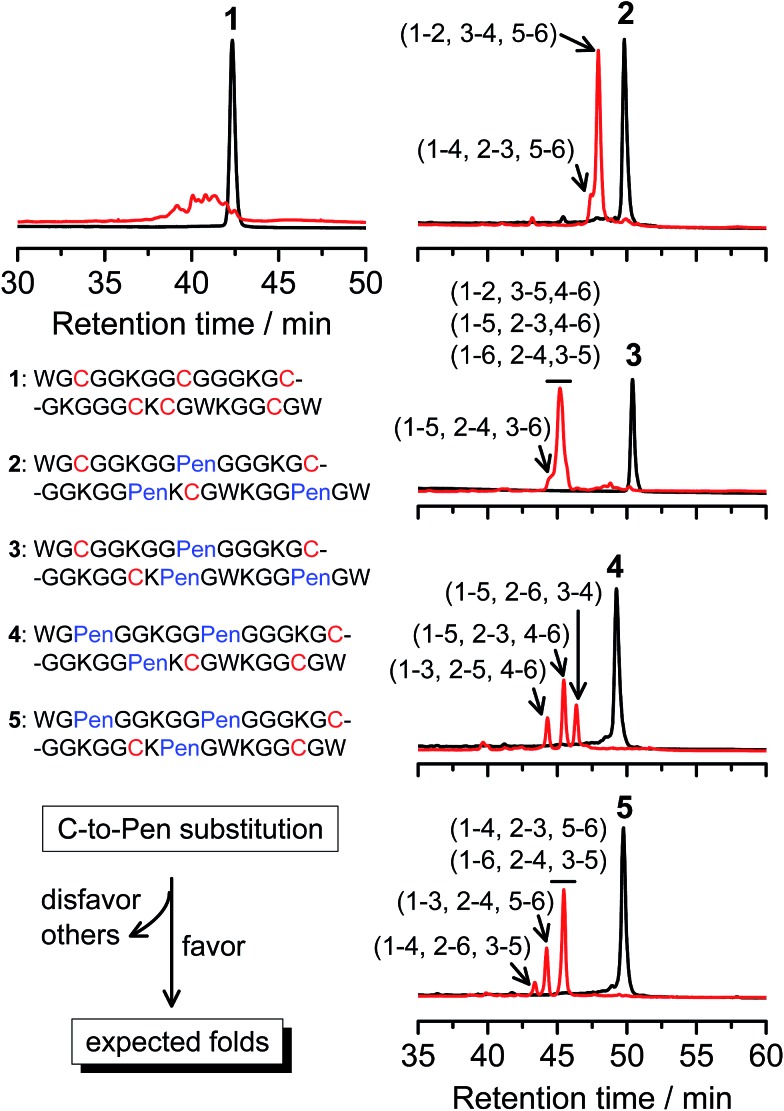
Amino acid sequences of peptides **1–5** (from N- to C-terminus). Chromatograms showing the oxidation of **1–5** (10 μM) in 0.2 mM GSSG (100 mM phosphate buffer, pH 7.4) (black line: before the oxidation; red line: 2 h after oxidative folding). C-to-Pen substitutions favor the folding of several expected folds and disfavor the folding of many others (see Fig. 1a). Disulfide pairings were established by tryptic digestion HPLC and mass spectrometry (Fig. S1–S14†).

A strategy to pull out four specific isomers from the total of 15 ones forming from the oxidation of a six-cysteine-bearing peptide was demonstrated first by transforming a model peptide with one CXC motif and four isolated cysteine residues (**1**) into its triple Pen-substituted analogs (**2–5**) (Fig. 1a). While the oxidation of the model peptide (**1**) results in the formation of scrambled products that cannot be efficiently isolated using high-performance liquid chromatography (HPLC) (Fig. 2; <20% conversion evaluated through the total peak area of the produced isomers relative to that of the reducing peptide), less than four products formed from the oxidation of each peptide from **2–5** (Fig. 2; >90% conversion, as either separable isomers or inseparable mixtures). Tryptic digestion analysis of the oxidizing products using HPLC and mass spectrometry indicated, as expected, that cysteine residues only paired heterogeneously with Pen residues and the formation of ring-closing CXPen or PenXC was not observed. Thus, in principle our designs to these peptides (**2–5**) should enable the highly specific formation of a subset of isomers with disulfide connectivities fully covering all possible isomers that might form from the model peptide **1** (Fig. 1). However, further analysis of the folded isomers suggests that two desired folds with disulfide bonds in a 1–4, 2–5, 3–6 (*i.e.*, knotted or &) and 1–6, 2–5, 3–4 (*i.e.*, laddered or #) arrangement were not obtained, which can be considered as two of the most compact folds in topology forming from six-cysteine peptides.^5^,^14^ Other isomers can be obtained in different yields likely depending on their conformational entropies, with the least compact fold with a disulfide arrangement of 1–2, 3–4, 5–6 (*i.e.*, bead-like fold) most efficiently formed (Fig. 2; ∼80% conversion). It is worth mentioning that the oxidative folding of these C/Pen-mixed peptides into specific isomers is certainly not driven by primary sequences because all peptides are composed of primarily achiral glycine residues; and some other lysine and tryptophan residues are strategically inserted for facilitating the tryptic digestion analyses. Our strategy to favor the folding of several specific isomers and yet disfavor many other undesired ones without the recourse to manipulation of primary sequences should be of particular interest to *de novo* design of proteins, particularly considering that the position of CXC motif, the length of each peptide segment, and the cysteine residues for Pen substitution might all be manipulated arbitrarily.

**Fig. 3:**
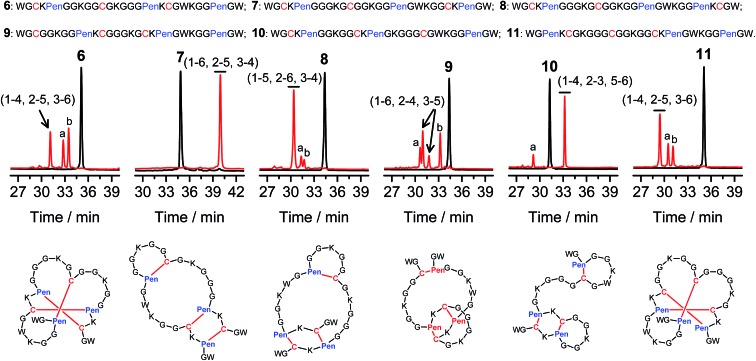
Amino acid sequences of peptides **6–11** (from N- to C-terminus). Chromatograms showing the oxidation of **6–11** (10 μM) in 0.2 mM GSSG (100 mM phosphate buffer, pH 7.4) (black line: before oxidation; red line: 2 h after oxidative folding). Topological drawings of the expected folds were given in bottom panels (red lines denote disulfide bonds; amino acid sequences can be read in a counter-clockwise direction). The disulfide connectivity of peaks a and b shown in the chromatograms (by products): **6**-a = (1–6, 2–5, 3–4), **6**-b = (1–4, 2–3, 5–6); **8**-a = (1–5, 2–3, 4–6), **8**-b = (1–4, 2–6, 3–5); **9**-a = (1–5, 2–4, 3–6), **9**-b = (1–2, 3–5, 4–6); **10**-a = (1–6, 2–3, 4–5); **11**-a = (1–4, 2–6, 3–5), **11**-b = (1–3, 2–5, 4–6). Disulfide pairings were established by tryptic digestion HPLC and mass spectrometry (Fig. S15–S36†). Of note, a pair of conformers formed from the folding of **9**, which have identical disulfide connectivity (1–6, 2–4, 3–5).

Then, we developed a strategy to direct the oxidative folding of peptides into specific isomers with topologically-formidable disulfide connectivities, including the two missing folds not obtained from the folding of **2–5**. This strategy takes advantage of the high propensity of disulfide pairing between two CXC motifs in peptide described previously.^11^ Though the overall yield of the pairing of two CXC motifs in six-cysteine peptides is relatively low (∼5–20%),^11^ here we demonstrate for the first time that the pairing of two CXPen and/or PenXC motifs in peptides (Fig. 1b) can achieve good to excellent yields (up to ∼100% in some cases), which enables the highly specific formation of topologically-formidable folds in high yields (*e.g.*, the folds with a laddered or a knotted disulfide connectivity). Peptides **6–10** were designed and synthesized (Fig. 3), into which a pair of CXPen and/or PenXC motifs are incorporated adjacently (**9** and **10**), alternately (**6**), or distantly at the N- and C-terminal regions (**7** and **8**). For these peptides, the total number of isomers formed after oxidative folding can be reduced, in principle, to a minimum of three. Our results clearly show that oxidation of **6** yielded the expected fold with a knotted disulfide connectivity (**6**-&) in ∼38% conversion as one of the three isomers (Fig. 3; totally ∼100% conversion); **7** and **8** can be oxidized to the expected folds with the laddered disulfide connectivity (**7**-#) and a connectivity of 1–5, 2–6, 3–4 in ∼100% and ∼80% conversion, respectively (Fig. 3). In addition, the specific disulfide pairings can still be preserved in the oxidation of **9** and **10**, though in these peptides the relative distance between the two pivotal motifs (*i.e.*, CXPen or PenXC) is obviously shorter compared to that in other peptides (**6–8**), a factor that can significantly disfavor the dimeric disulfide pairing of CXC motifs.^11^ We can still obtain the expected folds in ∼49% and ∼85% conversion after the oxidative folding of **9** and **10**, respectively, and importantly the fold with a connectivity of 1–6, 2–4, 3–5 from **9** are highly constrained in structure. For all of the peptides examined in this study, only ≤3 isomers were detected after the folding, and the disfavored folds against the specified C/Pen pairing rules were not observed.

The folds with their three disulfide bonds in a knotted arrangement are one of the most interesting scaffolds with exceptional stability and resistance to proteolytic degradation for peptide-based drug design.^5^,^15^ However, the *de novo* designed **6**-& can only be obtained in a moderate yield (∼38%). After analyzing side products forming from the oxidation of **6** (Fig. 3), we realized that the low yield of **6**-& is very likely caused by the relative easiness of forming one of the other two side-products that is obviously less compact in conformation; that is, the fold with a disulfide connectivity of 1–4, 2–3, 5–6 (or termed as “tandem fold”). Accordingly, to further increase the yield of the knotted fold, we strategically switched the position of the CXPen and PenXC motif in **6** to design and synthesize a new peptide **11**, which should retain the capability of forming a knotted fold (**11**-&), but without hindered by forming the structurally-incompact tandem or bead-like folds, based on the specified disulfide pairing rules. Interestingly, after oxidation, the yield of **11**-& was increased to 57%, obviously higher than that observed for **6** (Fig. 3). This result also demonstrated the feasibility of increasing the yield of desired folds through further optimizing the C/Pen pattern of peptides to disfavor competing folds.

**Fig. 4:**
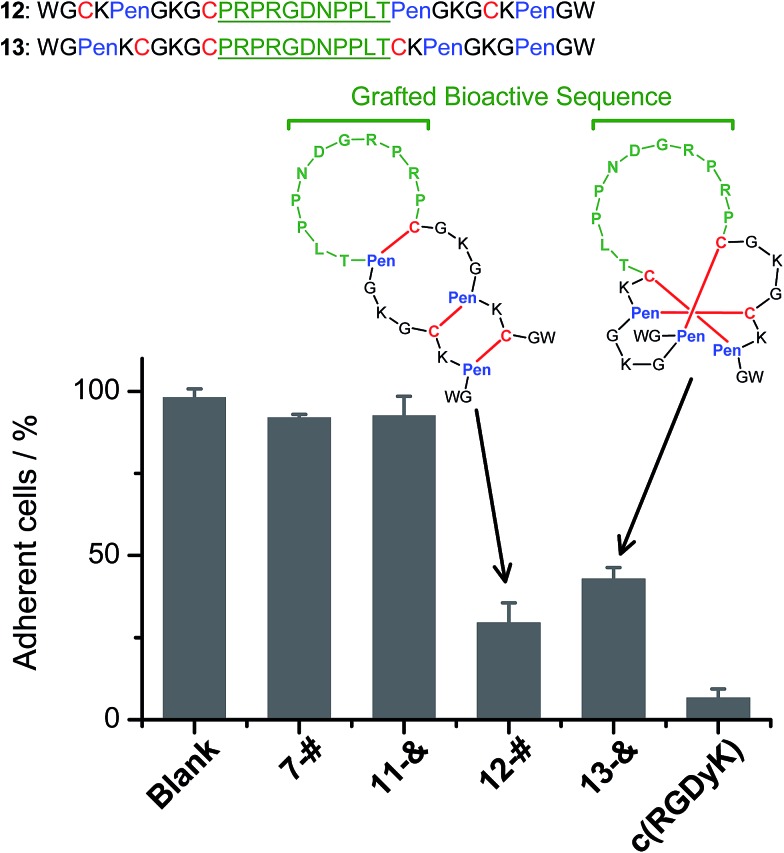
Amino acid sequences of peptides **12** and **13** (from N- to C-terminus); bioactive sequence grafted into the scaffolds was highlighted in green. The inhibition of U87MG cell adhesion by the peptides (**7**-#, **11**-&, **12**-#, **13**-&, and c(RGDyK); 50 μM/3 h) determined by MTT assays. **7**-# and **11**-& are negative controls (without the grafted bioactive sequence), which exhibit a negligible ability of inhibiting cell adhesion; c(RGDyK) is commercially available cyclic RGD, which was served as a positive control. The error bars represent the standard deviation of the mean (*n* = 3).

We further demonstrated that both **7**-# and **11**-& are tolerant to the grafting of bioactive peptide sequences (*i.e.*, bioactive epitopes). While a yeast-selected sequence (PRPRGDNPPLT; capable of binding to the cell-surface integrins) was grafted into the scaffolds (peptides **12** and **13**; the other two peptide segments was shortened by two residues for the ease of synthesis),^6^ the yields of the oxidative folding into the desired isomers (**12**-# and **13**-&) were only reduced moderately (∼63% and ∼70%, respectively; Fig. S37–S43†). In addition, both peptides can block the adhesion of U87 glioblastoma cells (with surface-expressed integrins^16^) to cell culture plates (Fig. 4), indicating the integrin-binding capability.

**Fig. 5:**
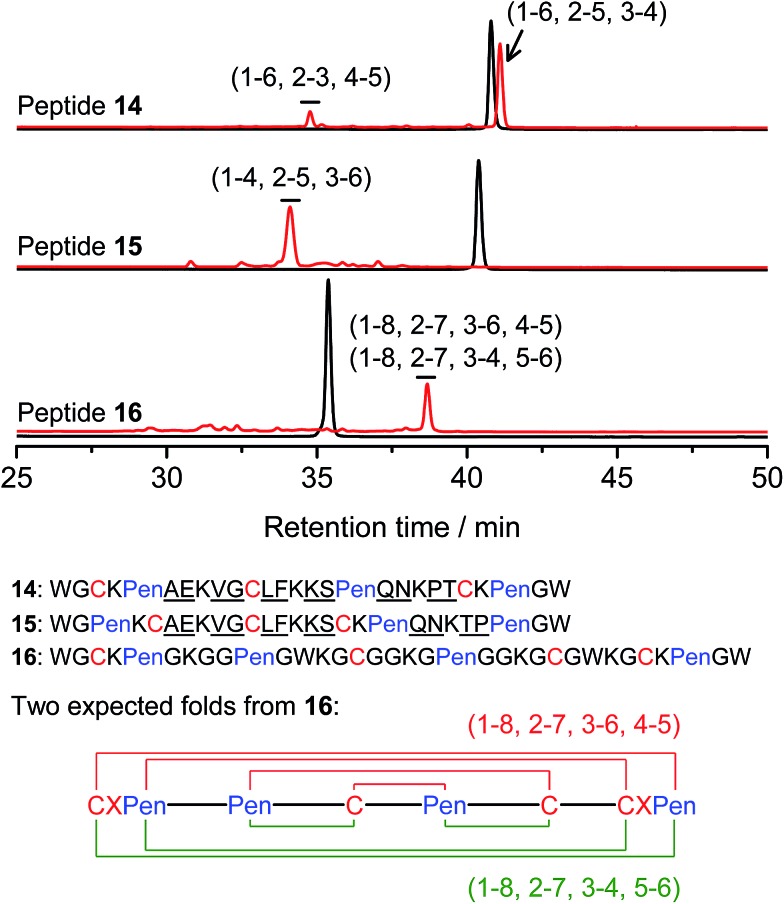
Amino acid sequences of peptides **14–16** (from N- to C-terminus); randomized amino acid residues were underlined (AE VG LF KS QN PT/TP). In peptide **15**, the residues threonine (T) and proline (P) following the lysine residue were exchanged to facilitate the analysis of disulfide pairings by the tryptic digestion HPLC and mass spectrometry. The length of the each variable segment in **16** was shortened by depletion of one amino acid residue to facilitate the synthesis. Chromatograms showing the oxidation of **14–16** (10 μM) in 0.2 mM GSSG (100 mM phosphate buffer, pH 7.4) (black line: before oxidation; red line: 2 h after oxidative folding). For the folding of **15** and **16**, other fully oxidizing isomers were not detected, and impurity peaks in the chromatograms should mainly come from trapped folding intermediates, which restrict overall yields of the expected folds. Disulfide pairings were established by tryptic digestion HPLC and mass spectrometry (Fig. S44–S50†).

Thus far, the tolerance of C/Pen-bearing peptide scaffolds to sequence randomizations has never been evaluated. The following designs and experiments will unambiguously demonstrate that even the peptide scaffolds (or folds) with the topologically-formidable disulfide connectivities (*i.e.*, **7**-# and **11**-&) are amenable to sequence randomization. A random 12-residue sequence was first generated by a sequence generator (cysteine residue excluded), which was then introduced into C/Pen-mixed frameworks with a laddering and knotting propensity, respectively, upon oxidative folding (Fig. 5; peptides **14** and **15**). A lysine residue is pre-placed into each peptide segment to facilitate the tryptic digestion analysis of disulfide connectivities of the folded products. After oxidation, the yields of **14**-# and **15**-& were ∼85% and ∼73%, respectively (Fig. 5), which were both comparable respectively to that observed for their glycine-rich analogs. It is worth mentioning that though peptides **14** and **15** comprise identical random-sequence segments, they can respectively fold into two topologically different folds. This finding further stresses that the sequence-specific folding effect on the formation of specific folds is, to some extent, negligible, compared to the C/Pen-directed disulfide pairing effects.

Finally, encouraged by the extremely high yield (∼100%) of **7**-# (*i.e.*, precise disulfide pairing of the two terminal CXPen motifs), we attempted to challenge the possibility of *de novo* designing constrained and sequence-independent peptide scaffolds with four disulfide bonds. Our design started with the C/Pen-mixed framework of **7**, into which an additional cysteine and Pen residue, as well as two additional glycine-rich segments, was introduced, which generates a new peptide **16** (Fig. 5). Owing to the high efficiency of dimeric pairing between the two CXPen motifs, we expected that two major isomers will form in a high yield after oxidation—because when the two terminal CXPen motifs are paired, the central Pen–C–Pen–C segment has two possible disulfide connectivities. Indeed, the result shown in Fig. 5 confirmed our prediction; and the two expected folds were obtained in a yield of ∼32% after oxidative folding. The yield is extremely high, considering that a peptide constrained through four disulfide bonds could, in theory, gain a total of up to 105 different structures (only based on possible disulfide connectivities).

## Conclusion

In summary, we have showed that structurally constrained and sequence-independent peptide scaffolds can be *de novo* designed by synergistically manipulating CXPen/PenXC motifs and the orthogonal disulfide pairing between cysteine and Pen residues. Particularly, the scaffolds (or folds) with topologically-formidable disulfide connectivities can be obtained from the oxidative folding of C/Pen-mixed peptides directly in good to excellent yields (∼38–100%). We have demonstrated that these scaffolds are tolerant to sequence manipulations, *e.g.*, bioactive sequence grafting and sequence randomization. The rules of disulfide pairing revealing in this study clearly indicate that the spatial arrangement of cysteine and Pen residues can efficiently force the oxidative folding of peptides to several specific isomers (1–4 folds) by disfavoring a substantial number of competing folds. To any specific fold of interest developed in this work for further computational design or random library developments, though the competing folds might still exist, the present scaffolds would provide a robust platform for generating constrained peptides that bind to targets of interest. Undoubtedly, with the present strategies for regulating oxidative folding of peptides in hand, disulfide-constrained peptides with unique and stable structures and/or new functions would be more conveniently designed or screened, because most non-native folds have already been pre-excluded by the specified disulfide pairing rules. So far, almost all disulfide-rich peptide scaffolds are vitally correlated to their primary sequences, but ours are exceptional ones. Moreover, this work also offers an approach to emerging fields such as the precision folding of synthetic polymers and topological polymer chemistry.^17^

## Materials and methods

### Materials

Fmoc-protected amino acids, Rink amide-AM resins and 2-chlorotrityl hydrazine resin were supplied by GL Biochem (Shanghai, China). All other chemicals used for peptide syntheses and analyses were purchased from suppliers, including Energy Chemical (Shanghai, China), Sigma-Aldrich (Shanghai, China), Alfa Aesar (Beijing, China), and TCI (Shanghai, China).

### Synthesis of peptides

All peptides were synthesized using solid-phase peptide synthesis (SPPS) and native chemical ligation methods according to reported procedures (detailed steps were described in the ESI, Pages S3–S5†).^18^ The peptides were purified by analytical and semi-preparative RP-HPLC and characterized using mass spectrometry (mass spectra were given in the ESI, Pages S93–S98†).

### Oxidative folding of peptides

Fully reduced peptides were dissolved in 100 mM phosphate buffer (pH 7.4) containing 0.2 mM GSSG to achieve a concentration of ∼10 μM. Two hours later, aliquots were taken into an empty tube and were immediately treated with 10% HPO_3_ to quench the oxidative reactions. The samples were then analyzed using an analytical HPLC (HPLC gradient for the analysis was given in the ESI†).

### Analysis of disulfide pairing in peptides

Certain amounts of oxidized peptides (isolated by HPLC, ∼30 μM) were dissolved in 90 μL phosphate buffer (100 mM, pH = 7.4), which were then digested by the addition of 10 μL aqueous solution of trypsin (1 mg mL^–1^) at room temperature (∼0.5 h). The digested fragments were then analyzed by HPLC and MS.

### Integrin-dependent cell adhesion assays

Cell adhesion assays were evaluated by the MTT assay reported previously in our lab.^13^ U87MG cells were plated into 24-well plates at an initial cell density of 80 000 cells per well and grown overnight at 37 °C, 5% CO_2_. After 24 h, the medium was removed and the peptides were added to each well in 300 μL DMEM contained FBS, incubated at 37 °C, 5% CO_2_ for 3 h. After that, the medium was removed and cells were washed three times with DMEM to remove the detached cells. Then 300 μL fresh DMEM and 30 μL MTT (50 mg mL^–1^) was added to each well. The cells were incubated for 4 h at 37 °C in culture hood. After that, remove the supernate and add 300 μL MTT solvent (DMSO). Cover with tinfoil and agitate cells on orbital shaker for 15 min. The absorbance was then measured at 490 nm using a plate reader (PerkinElmer Enspire®). The obtained absorbance was blank-corrected (blank: DMEM + MTT, no cells) and the cell viability in percent was calculated according to the following equation:Cell viability = (OD_490,sample_/OD_490,control_) × 100%where OD_490,sample_ and OD_490,control_ represent the optical density of the cells treated with peptides and that of the cells only treated with DMEM, respectively.

## Conflicts of interest

There are no conflicts to declare.

## Supplementary Material

Supplementary information
