# Supplementary material for: De novo design of constrained and sequence-independent peptide scaffolds with topologically-formidable disulfide connectivities
Source: Chem Sci. 2017 Nov 20;9(3):569–75. doi: 10.1039/c7sc03956e (PMC5869988; doi:10.1039/c7sc03956e)
Supplement: Supplementary file 1 [file SC-009-C7SC03956E-s001.pdf]

Electronic Supplementary Material (ESI) for Chemical Science.
This journal is © The Royal Society of Chemistry 2017

# Electronic Supplementary Information for

# *De Novo* Design of Constrained and Sequence-Independent Peptide Scaffolds with Topologically-Formidable Disulfide Connectivities

Yiwu Zheng, Xiaoting Meng, Yaqi Wu, Yibing Zhao, Chuanliu Wu*

*Department of Chemistry, College of Chemistry and Chemical Engineering, State Key Laboratory of Physical Chemistry of Solid Surfaces, The MOE Key Laboratory of Spectrochemical Analysis and Instrumentation, Xiamen University, Xiamen, 361005, P.R. China.*

*To whom correspondence should be addressed, Email: chlwu@xmu.edu.cn

# Table of contents

**Experimental Section……………………………………………………………….……………..3**

1) **Materials**
2) **HPLC gradient**
3) **Preparation of 2-chlorotrityl hydrazine resin**.
4) **Peptide synthesis.**
5) **Native chemical ligation.**

**Figure | S1-S50 Tryptic digestion HPLC/MS analysis of disulfide pairing for peptides………..7**

**Characterization of peptides…………………………………………………………………93**

# Experimental Section

**1) Materials**. Fmoc-protected amino acids, Rink amide-AM resin and 2-chlorotrityl hydrazine resin were supplied by GL Biochem. Tris(2-carboxyethyl)phosphine hydrochloride (TCEP), trifluoroacetic acid (TFA), 1-hydroxybenzotriazole (HOBT), N,N'-diisopropylcarbodiimide (DIC) and ethyldiisopropylamine (DIEA) were bought from Energy Chemical (Shanghai, China), L(-)-Glutathione and 3-4,5-dimethyl-2-thiazolyl)-2,5-diphenyl-2-H-tetrazolium bromide (MTT) was purchased from Sigma-Aldrich (Shanghai, China). 4-Mercaptophenylactic acid (MPAA) was purchased from Alfa Aesar (Beijing, China). Thioanisole and 1,2-ethanedithiol (EDT) were purchased from TCI (Shanghai, China). U87 cells were purchased from CoBioer Biosciences CO., LTD (Nanjing, China). Dulbecco's Modified Eagle Medium (DMEM) with high glucose and phosphate buffered saline (PBS) were obtained from Thermo Scientific (Beijing, China). Eppendorf tubes (1.5 mL), 24-well chambers and cell culture dishes were purchased from JET BIOFIL (Guangzhou, China). was purchased from Sigma-Aldrich.

**2) HPLC gradient**

**Method 1:** 1.0 mL.min$^{-1}$ flow rate, isocratic with water (+0.1 % *v/v* TFA) for 5 min followed by a linear gradient of ACN (+0.1 % *v/v* TFA) over 40 min (0–95 % *v/v*)).

**Method 2:** 1.0 mL.min$^{-1}$ flow rate, isocratic with water (+0.1 % *v/v* TFA) for 5 min followed by a linear gradient of ACN (+0.1 % *v/v* TFA) over 80 min (0–95 % *v/v*)).

**Method 3:** 1.0 mL.min$^{-1}$ flow rate, isocratic with water (+0.1 % *v/v* TFA) for 5 min followed by a linear gradient of ACN (+0.1 % *v/v* TFA) over 160 min (0–95 % *v/v*)).

**3) Preparation of 2-chlorotrityl hydrazine resin**. 2-Chlorotrityl hydrazine resin was synthesized from 2-chlorotrityl chloride resin using a procedure adapted from L, Liu et al. *Angew. Chem. Int. Ed.*

2012, **51**, 10347–10350. 2-Chlorotrityl chloride resin (1.5 g) was swelled in DMF (10 mL) for 15 min. hydrazine (0.5 mL) and DIEA (2 mL) were dissolved in DMF (5 mL). The above solution was then added dropwise into the resin and stirred for 1.5 h at room temperature. The reaction was quenched by adding 1 mL of methanol and then stirred for 20 min. The resulting resin was washed with DMF, $H_2O$, DMF, MeOH and $Et_2O$ in sequence. The resin was then dried and determined to have a loading of 0.857 mmol/g.

**4) Peptide synthesis.** All Peptides were synthesized by the Fmoc solid-phase method by using Liberty Blue™ Automated Microwave Peptide Synthesizer (CEM Corporation). Unless noted, coupling of the amino acid to the resin was performed within the reaction vessel using standard coupling (50℃ coupling for Cys, Pen) and standard deprotection (Fmoc-protected amino acid (0.2 M, 1.25 mL), HOBT (1 M, 0.5 mL), DIC (0.5 M, 1mL), The Fmoc protecting group was cleaved with 20% piperidine/DMF), peptides were cleaved from the resin and side chains were deprotected with a standard cleavage cocktail containing 2.5% (v/v) EDT, 2.5% (v/v) $H_2O$, and 5% (v/v) TIPS in TFA for 3 hours at room temperature.

**Standard Coupling:**

| Stage | Temp (℃) | Power (w) | Time (sec) | Delta T |
|---|---|---|---|---|
| 1 | 75 | 170 | 15 | 2 |
| 2 | 90 | 30 | 110 | 1 |

**50℃ coupling:**

| Stage | Temp (℃) | Power (w) | Time (sec) | Delta T |
|---|---|---|---|---|

| | | | | |
|---|---|---|---|---|
| 1 | 25 | 0 | 120 | 2 |
| 2 | 50 | 35 | 1080 | 1 |

**Standard deprotection:**

| Stage | Temp (°C) | Power (w) | Time (sec) | Delta T |
|---|---|---|---|---|
| 1 | 75 | 155 | 15 | 2 |
| 2 | 90 | 30 | 50 | 1 |

**5) Native chemical ligation.** The general procedure for ligation of peptides using peptide hydrazides was followed as reported in L, Liu. et al. *Angew. Chem. Int. Ed.* 2012, **51**, 10347–10350. Taking the synthesis of peptide **1** for example (All peptides were synthesized using the same protocol used for the synthesized of the peptide **1**). Ac-WGCGGKGGCGGGKG-$NHNH_2$ (5 mg, 4.0 μmol) was dissolved in 0.8 mL ligation buffer at pH 3.3 (0.2 M phosphate solutions containing 6 M Gn·HCl.) The solution was stirred in an ethanol bath at –14°C, Then, 80 μL of $NaNO_2$ (500 mM, aq.) was added dropwise, and stirred for 30 min. After that, 0.8 mL of MPAA (500 mM) was added to the solution in order to quench the $NaNO_2$ and convert the acyl azide into the corresponding MPAA thioester. The pH of the mixed solution was slowly adjusted to pH 7.0 with 6 M NaOH, and CGKGGGCKCGWKGGCGW-$NH_2$ (10 mg, 5.9 μM) was added. Gently agitate the solution by magnetic stirring for 3 h. After that, the reaction solution was reduced by TCEP (50 mM, aq.) and was purified over a semi-preparative HPLC. The collected products was lyophilized and dissolved in $H_20$ (0.1% TFA), and then further purified by $C^{18}$ reversed-phase analytical HPLC. The concentration of peptide was calculated by using Beer–Lambert's law with the molar extinction coefficient of tryptophan (5502 $cm^{-1}$ $m^{-1}$ at 280 nm).

| | Peptide sequences |
|---|---|
| 1 | Ac-WGCGGKGGCGGGKGCGKGGGCKCGWKGGCGW-$NH_2$ |
| 2 | Ac-WGCGGKGGPenGGGKGCGGKGGPenKCGWKGGPenGW-$NH_2$ |
| 3 | Ac-WGCGGKGGPenGGGKGCGGKGGCKPenGWKGGPenGW-$NH_2$ |
| 4 | Ac-WGPenGGKGGPenGGGKGCGGKGGPenKCGWKGGCGW-$NH_2$ |
| 5 | Ac-WGPenGGKGGPenGGGKGCGGKGGCKPenGWKGGCGW-$NH_2$ |
| 6 | Ac-WGCKPenGGKGGCGKGGGPenKCGWKGGPenGW-$NH_2$ |
| 7 | Ac-WGCKPenGGGKGCGGKGGPenGWKGGCKPenGW-$NH_2$ |
| 8 | Ac-WGCKPenGGGKGCGGKGGPenGWKGGPenKCGW-$NH_2$ |
| 9 | Ac-WGCGGKGGPenKCGGGKGCKPenGWKGGPenGW-$NH_2$ |
| 10 | Ac-WGCKPenGGKGGCKPenGKGGGCGWKGGPenGW-$NH_2$ |
| 11 | Ac-WGPenKCGKGGGCGGKGGCKPenGWKGGPenGW-$NH_2$ |
| 12 | Ac-WGCKPenGKGCPRPRGDNPPLTPenGKGCKPenGW-$NH_2$ |
| 13 | Ac-WGPenKCGKGCPRPRGDNPPLTCKPenGKGPenGW-$NH_2$ |
| 14 | Ac-WGCKPenAEKVGCLFKKSPenQNKPTCKPenGW-$NH_2$ |
| 15 | Ac-WGPenKCAEKVGCLFKKSCKPenQNKTPPenGW-$NH_2$ |
| 16 | Ac-WGCKPenGKGGPenGWKGCGGKGPenGGKGCGWKGCKPenGW-$NH_2$ |
| Note | **The green cysteine means the ligation site.** |

# Supplementary Data

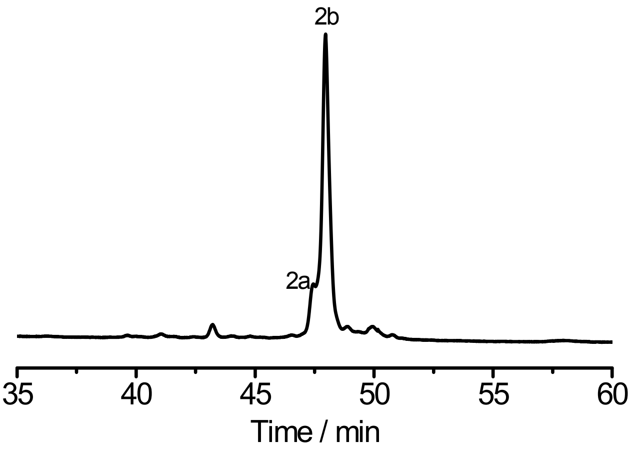


**Figure S1.** Chromatogram of the products formed after the oxidation of peptide **2**.

a)

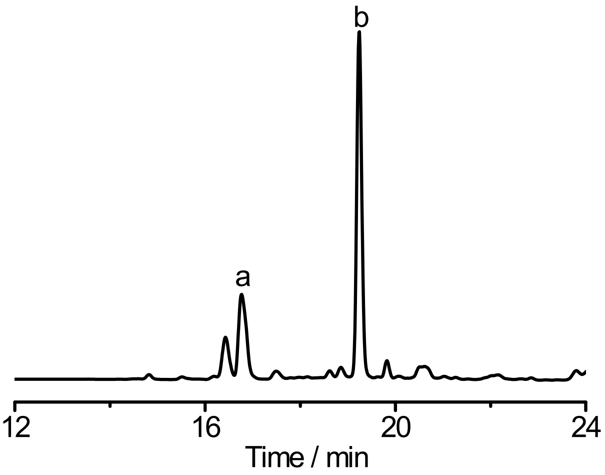


b)

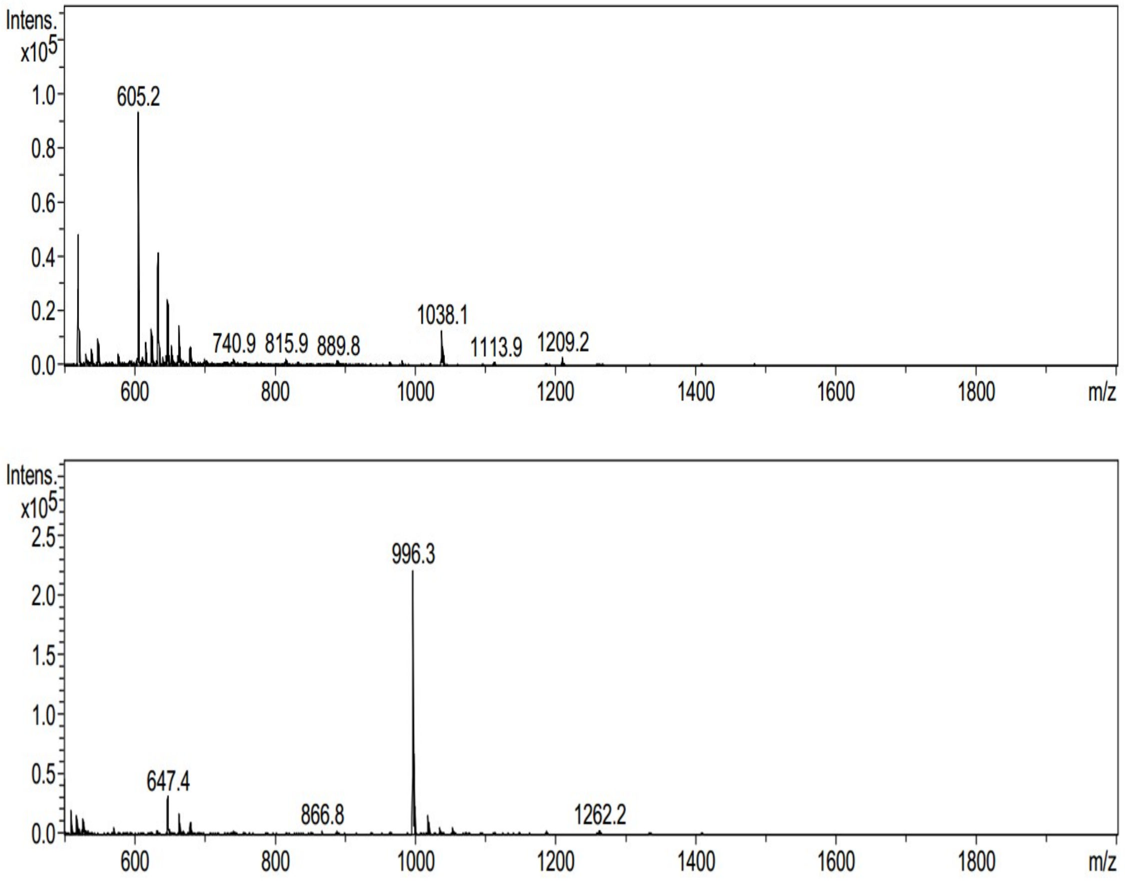


**Figure S2.** Tryptic digestion HPLC/MS analysis of **2a**: a) chromatogram of digested fragments from 2**a**; b) mass spectra of fragment a and b labeled in the chromatogram.

**Fragment analysis:**

| Peak NO. | Disulfide pairing | *m/z*$(M)^+$ expected | *m/z*$(M)^+$ or$(M+2H)^{2+}$ found |
|---|---|---|---|
| a | 1- 4 | 1038.23 | 1038.1/519.7 |
| b | 5-6 | 996.18 | 996.3 |

The arrow indicates the cleavage site of trypsin digestion.

Ac-WGCGGK↓GGPenGGGK↓GCGGK↓GGPenK↓CGWK↓GGPenGW-$NH_2$

1 2 3 4 5 6

Th

ese fragments indicate the formation of 1-4, 2-3, 5-6

a)

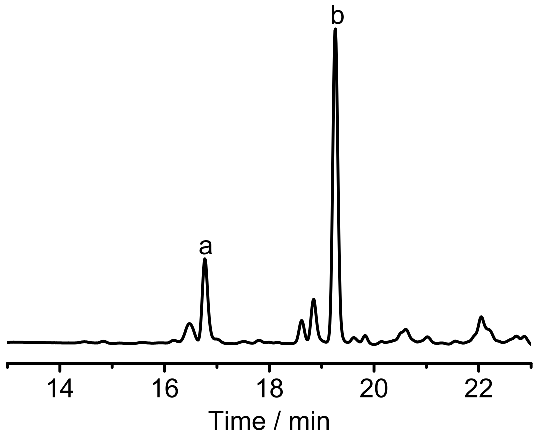


b)

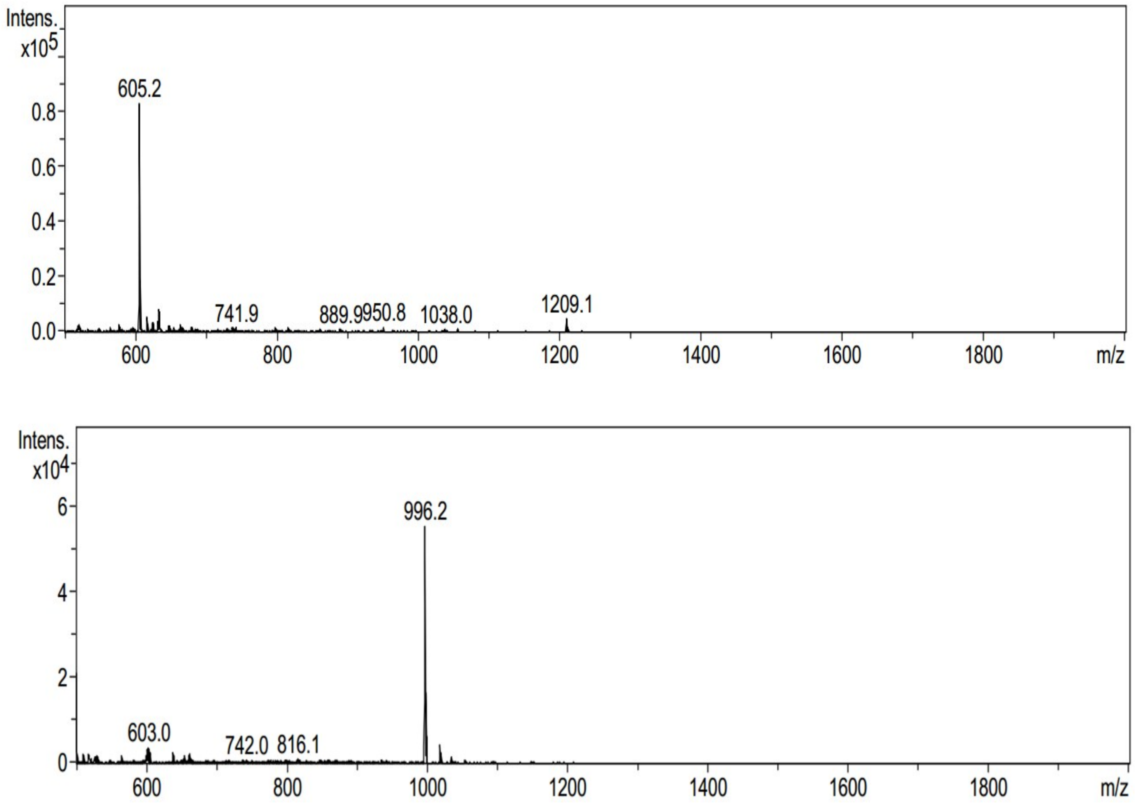


**Figure S3.** Tryptic digestion HPLC/MS analysis of **2b**: a) chromatogram of digested fragments from **2b**; b) mass spectra of fragments a and b labeled in the chromatogram.

**Fragment analysis:**

| **Peak NO.** | **Disulfide pairing** | ***m/z*(M)$^+$ expected** | ***m/z*(M)$^+$or(M+2H)$^{2+}$ found** |
|---|---|---|---|
| a | 1-2 | 1209.38 | 1209.1/605.2 |
| b | 5-6 | 996.18 | 996.2 |

The arrow indicates the cleavage site of trypsin digestion.

Ac-WGCGGK↓GGPenGGGK↓GCGGK↓GGPenK↓CGWK↓GGPenGW-$NH_2$

1 2 3 4 5 6

These fragments indicate the formation of 1-2, 3-4, 5-6.

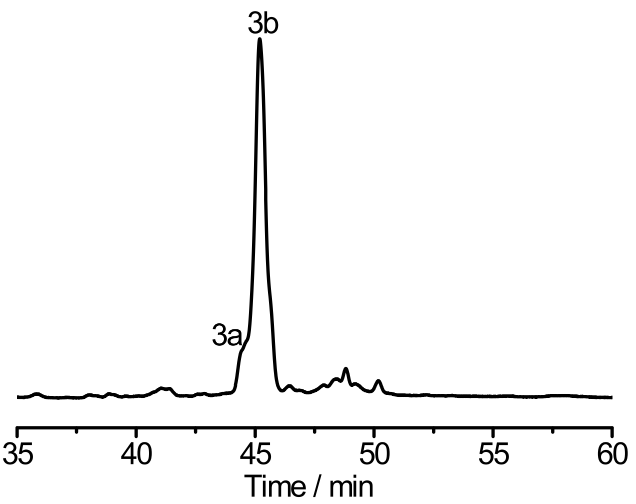


**Figure S4.** Chromatogram of the products formed after the oxidation of peptide **3**.

a)

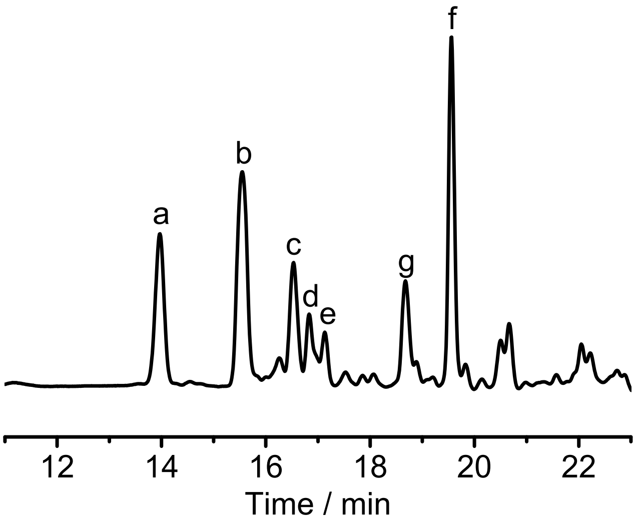


b)

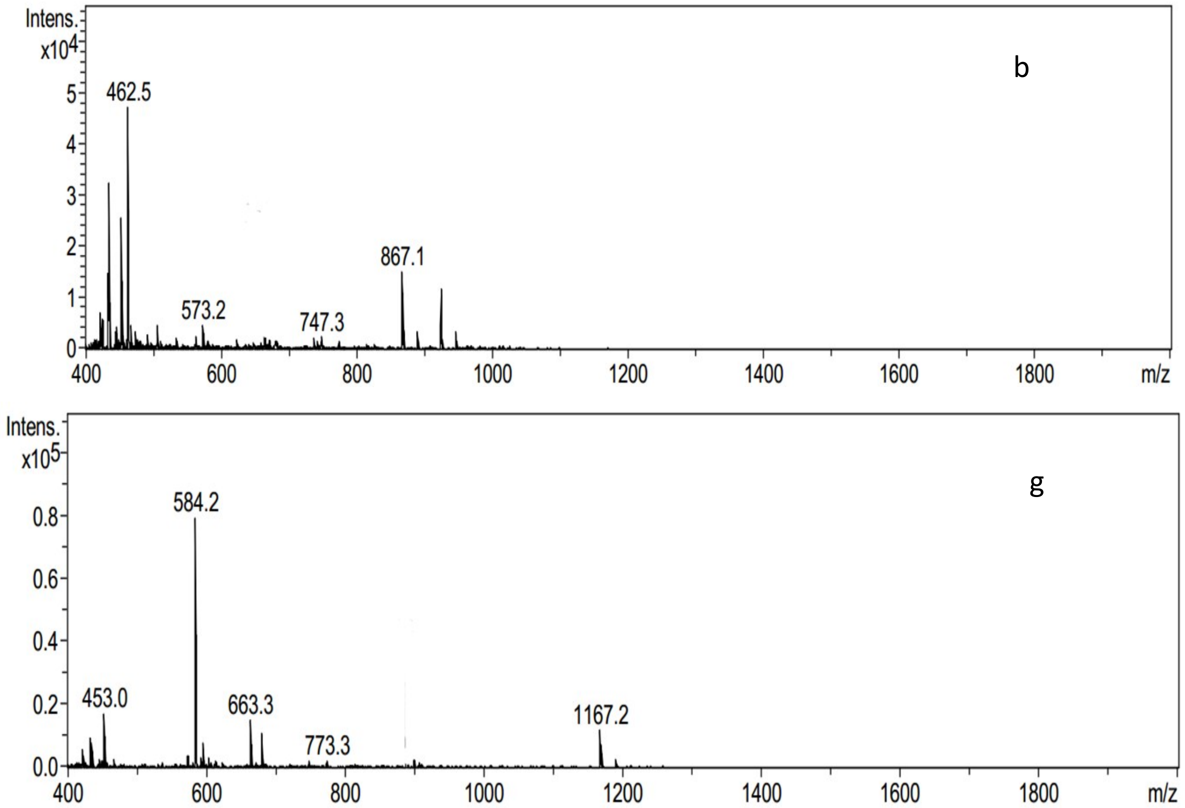


**Figure S5.** Tryptic digestion HPLC/MS analysis of **3a**: a) chromatogram of digested fragments from **3a**; b) mass spectra of fragments b and g labeled in the chromatogram.

**Fragment analysis:**

| Peak NO. | Disulfide pairing | *m/z*$(M)^+$ expected | *m/z*$(M)^+$or$(M+2H)^{2+}$ found |
|---|---|---|---|
| b | 3-6 | 924.08 | 924.1/462.5 |
| | and | | |
| | 4-6 | 867.03 | 867.1 |
| g | 1-5 | 1167.38 | 1167.2/584.2 |

The arrow indicates the cleavage site of trypsin digestion.

Ac-WGCGGK↓GGPenGGGK↓GCGGK↓GGCK↓PenGWK↓GGPenGW-$NH_2$

1 2 3 4 5 6

By carefully analyzing the fragments of enzyme digestion of **3a** and **3b**, 3-6 pairing (Fragments b) was only found in isomer **3a**. Therefore, it is reasonable to presume that the isomer **3a** with a connectivity of I-V, II-Ⅳ, III-Ⅵ from fragments b and g.

Note that Fragments a, c, d, e and f was a contaminant from isomer **3b** that was difficult to remove, 4-6 pairing in fragment b was a contaminant from isomer **3b**.

a)

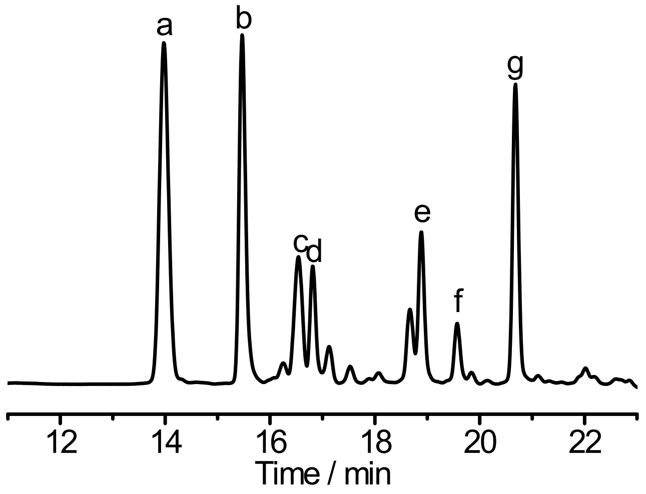
a
b
c
d
e
f
g
12
14
16
18
20
22
Time / min

b)

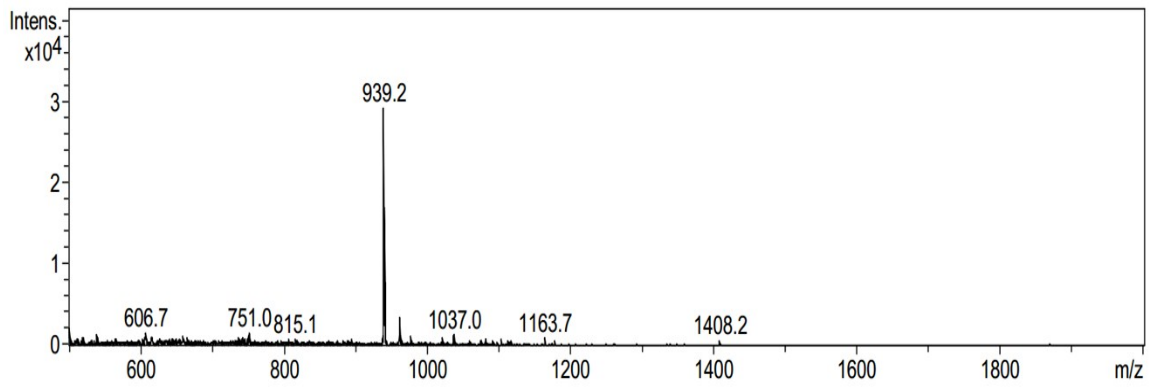
Intens.
x10^4
3
2
1
0
939.2
606.7
751.0
815.1
1037.0
1163.7
1408.2
600
800
1000
1200
1400
1600
1800
m/z

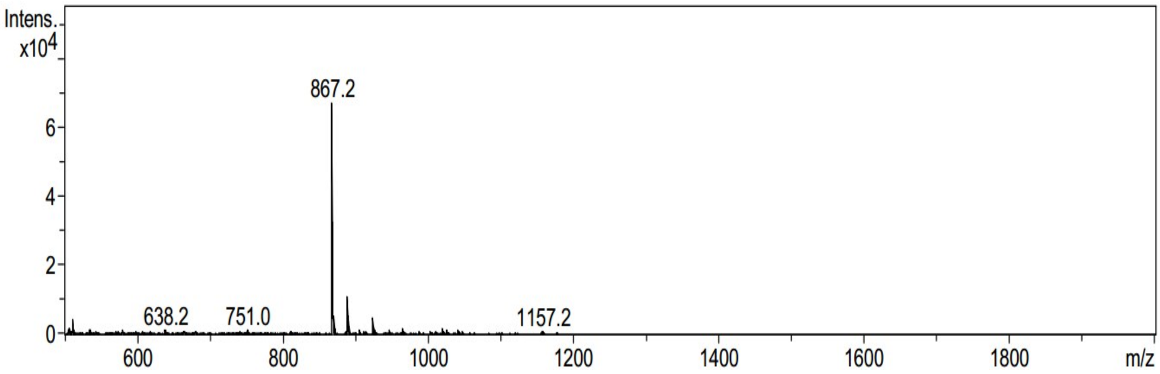
Intens.
x10^4
6
4
2
0
867.2
638.2
751.0
1157.2
600
800
1000
1200
1400
1600
1800
m/z

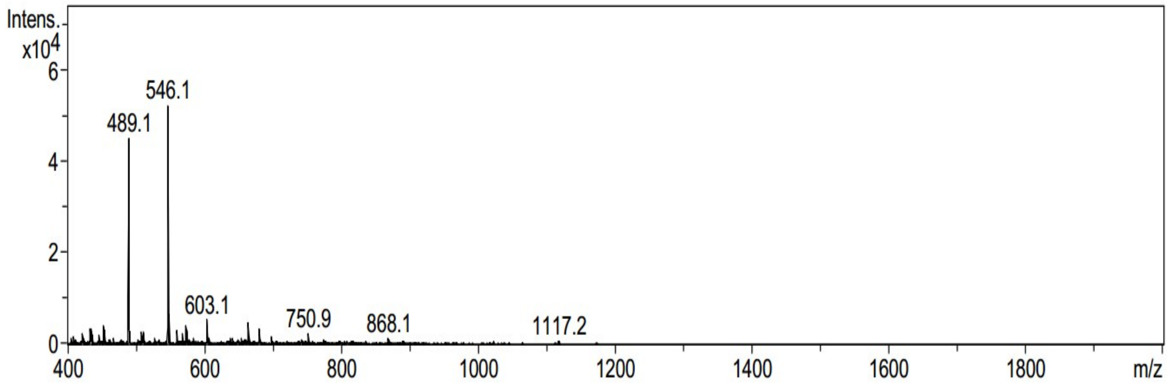
Intens.
x10^4
6
4
2
0
546.1
489.1
603.1
750.9
868.1
1117.2
400
600
800
1000
1200
1400
1600
1800
m/z

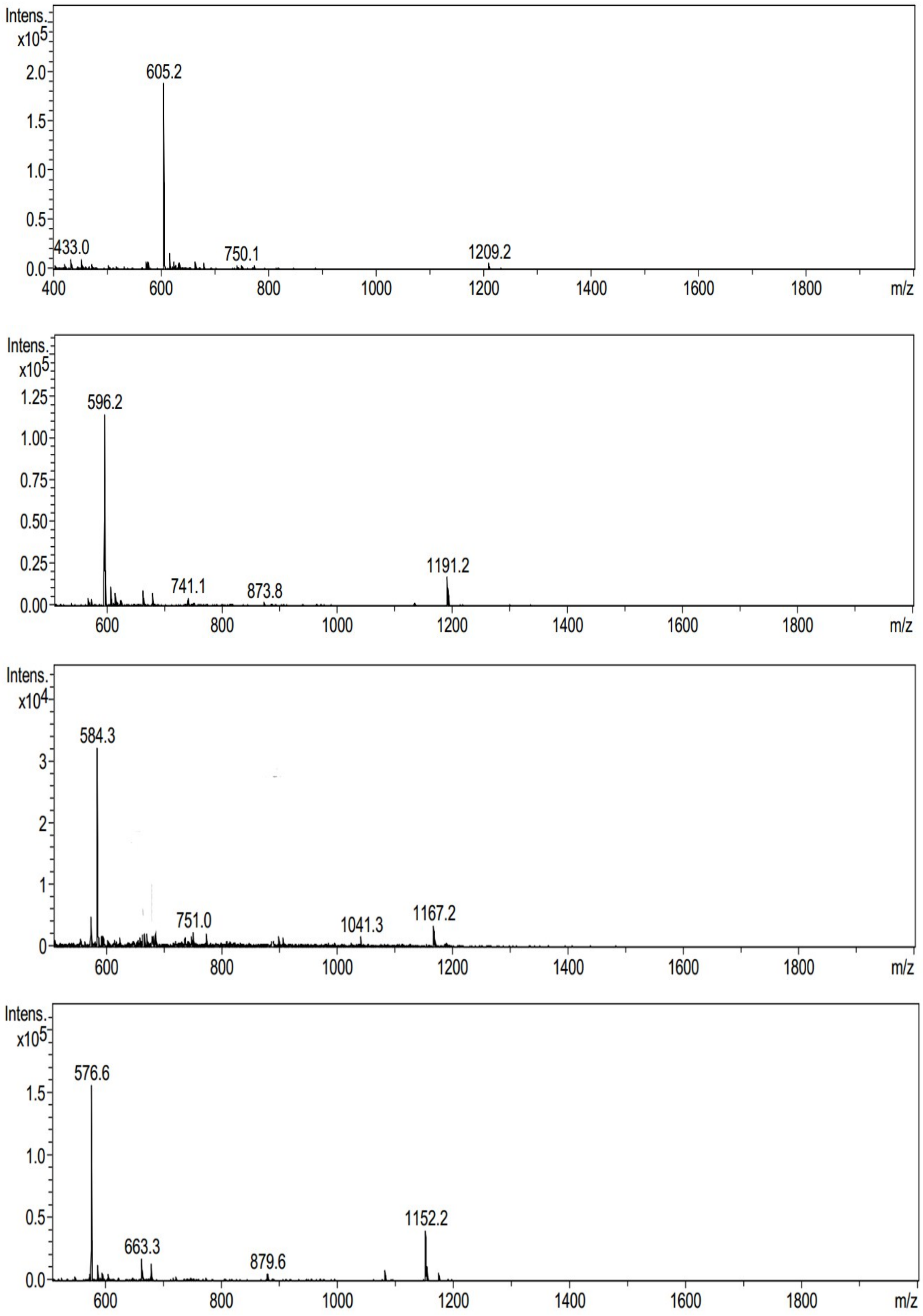


**Figure S6.** Tryptic digestion HPLC/MS analysis of **3b**: a) chromatogram of digested fragments from **3b**; b) mass spectra of fragments a-g (from top to bottom) labeled in the chromatogram.

**Fragment analysis:**

| **Peak NO.** | **Disulfide pairing** | ***m/z*(M+H)$^+$ expected** | ***m/z*(M+H)$^+$or(M+2H)$^{2+}$ found** |
|---|---|---|---|
| a | 3-5 | 939.13 | 939.2 |
| b | 4-6 | 867.03 | 867.2 |
| c | unknown peak | | 546.1/489.1 |
| d | 1-2 | 1209.38 | 1209.2/605.2 |
| e* | 1-2 | 1191.38 | 1191.2/596.2 |
| f | 1-5 | 1167.38 | 1167.2/584.3 |
| g | 1-6 | 1152.33 | 1152.2/576.6 |

The arrow indicates the cleavage site of trypsin digestion.

Ac-WGCGGK↓GGPenGGGK↓GCGGK↓GGCK↓PenGWK↓GGPenGW-$NH_2$
1 2 3 4 5 6

e*: the cleavage site within the WGCGGKGGPenGGGK fragment was not cleaved.

Fragments a, b,d and e* indicate the formation of 1-2, 3-5, 4-6.

Fragments b and f indicate the formation of 1-5, 2-3, 4-6.

Fragments a and g indicate the formation of 1-6, 2-4, 3-5.

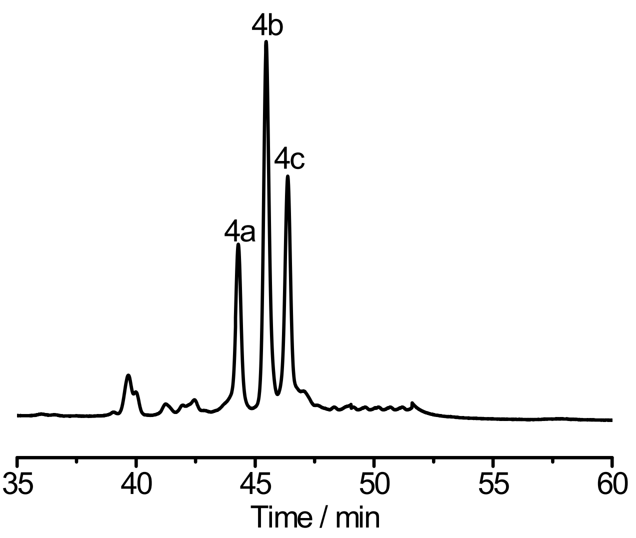


**Figure S7.** Chromatogram of the products formed after the oxidation of peptide **4**.

a)

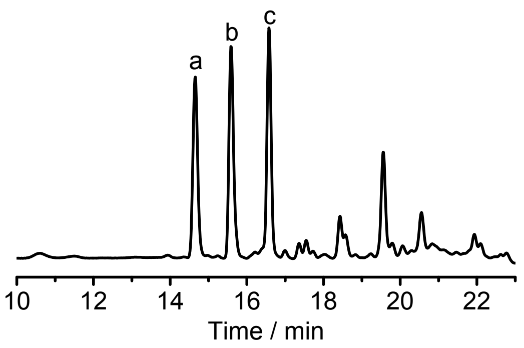


b)

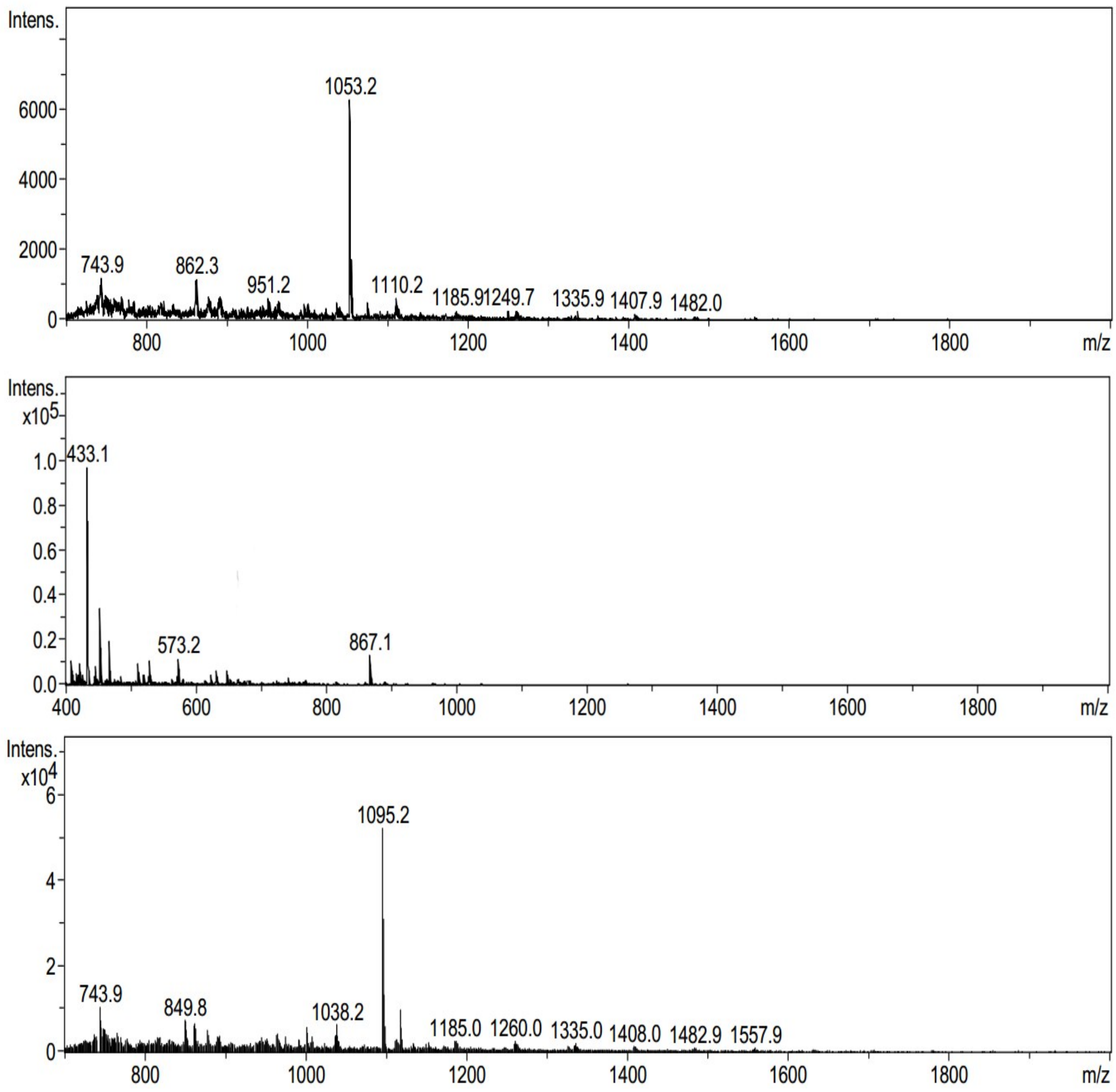


**Figure S8.** Tryptic digestion HPLC/MS analysis of **4a**: a) chromatogram of digested fragments from **4a**; b) mass spectra of fragments a-c (from top to bottom) labeled in the chromatogram.

**Fragment analysis:**

| **Peak NO.** | **Disulfide pairing** | ***m/z*(M)$^+$ expected** | ***m/z*(M)$^+$or(M+2H)$^{2+}$ found** |
|---|---|---|---|
| a | 2-5 | 1053.23 | 1053.2 |
| b | 4-6 | 867.03 | 867.1/433.1 |
| c | 1-3 | 1095.28 | 1095.2 |

The arrow indicates the cleavage site of trypsin digestion.

Ac-WGPenGGK↓GGPenGGGK↓GCGGK↓GGPenK↓CGWK↓GGCGW-$NH_2$

1 2 3 4 5 6

These fragments indicate the formation of 1-3, 2-5, 4-6

a)

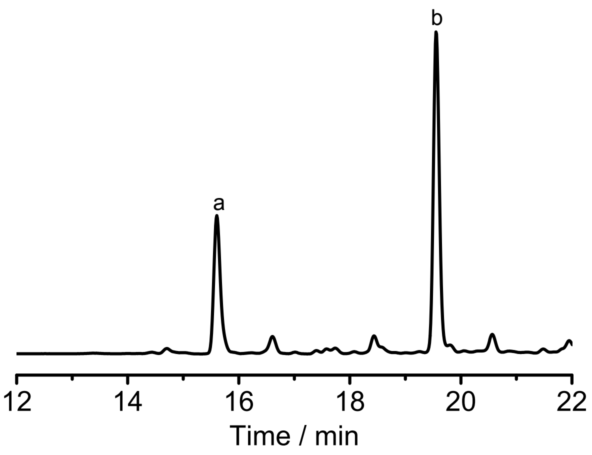


b)

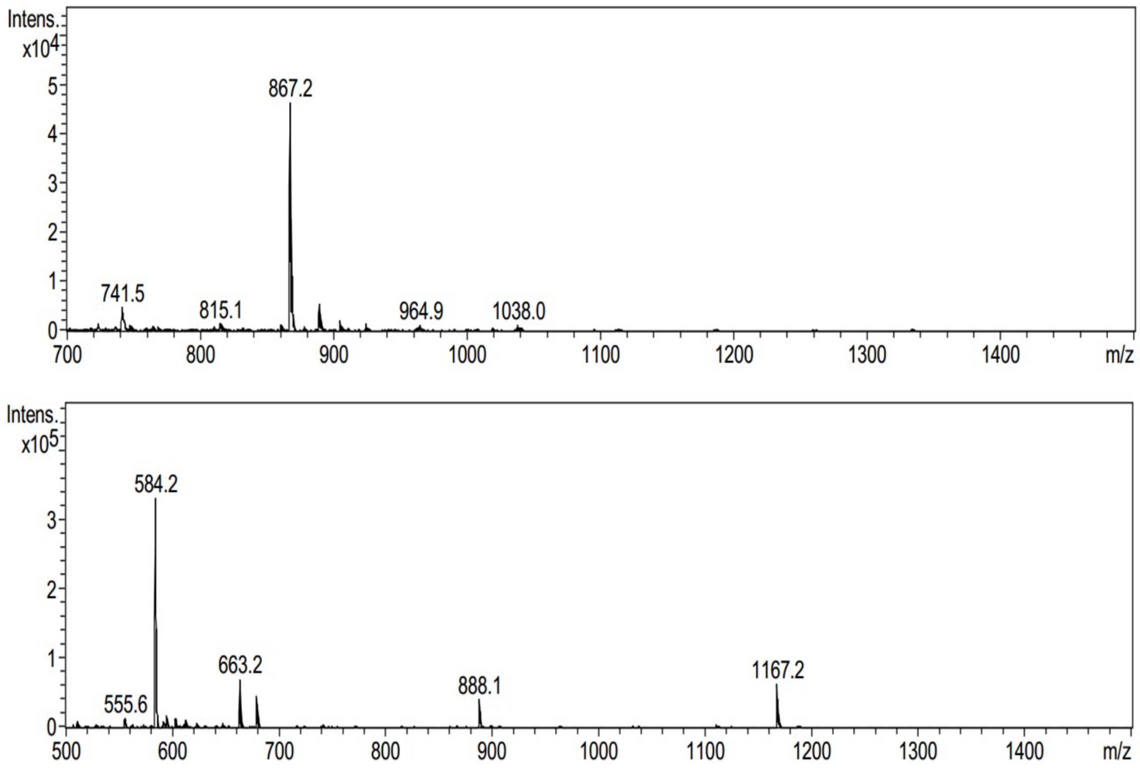


**Figure S9.** Tryptic digestion HPLC/MS analysis of **4b**: a) chromatogram of digested fragments from **4b**; b) mass spectra of fragment a and b labeled in the chromatogram.

**Fragment analysis:**

| Peak NO. | Disulfide pairing | $m/z(\mathrm{M})^+$ expected | $m/z(\mathrm{M})^+$or$(\mathrm{M+2H})^{2+}$ found |
|---|---|---|---|
| a | 4-6 | 867.03 | 867.1/433.1 |
| b | 1-5 | 1167.38 | 1167.2/584.2 |

The arrow indicates the cleavage site of trypsin digestion.

Ac-WGPenGGK↓GGPenGGGK↓GCGGK↓GGPenK↓CGWK↓GGCGW-$NH_2$

1 2 3 4 5 6

These fragments indicate the formation of 1-5, 2-3, 4-6.

a)

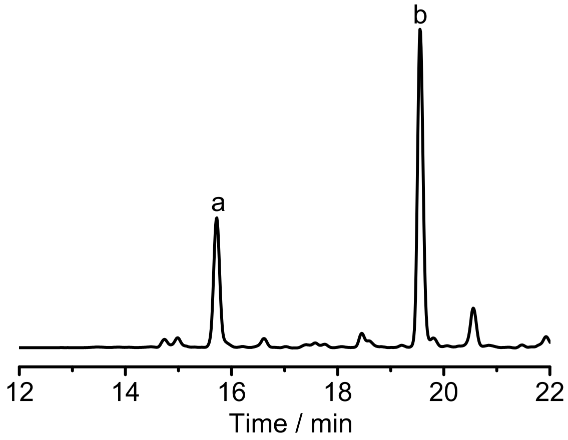


b)

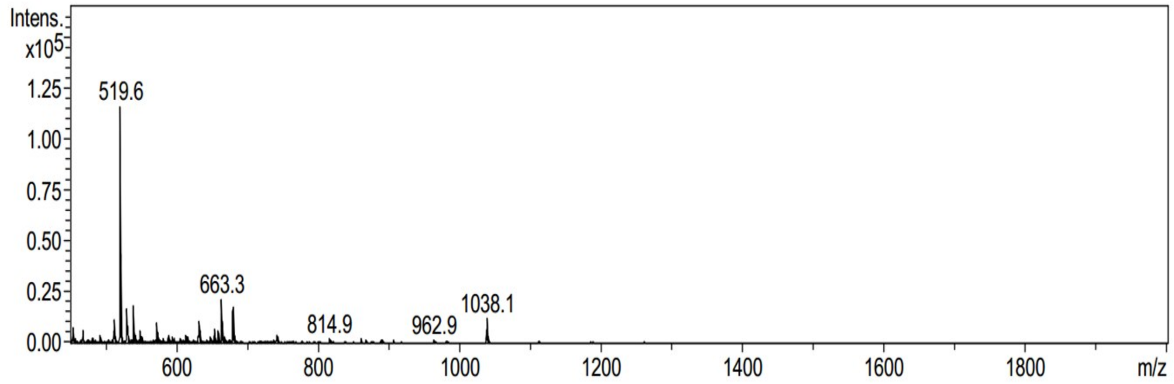


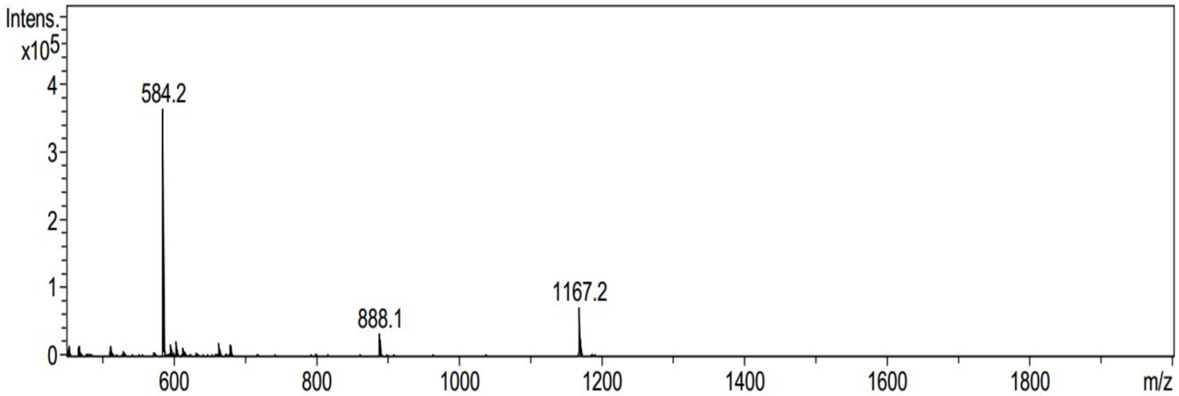


**Figure S10.** Tryptic digestion HPLC/MS analysis of **4c**: a) chromatogram of digested fragments from **4c**; b) mass spectra of fragments a and b labeled in the chromatogram.

**Fragment analysis:**

| Peak NO. | Disulfide pairing | *m/z*(M)$^+$ expected | *m/z*(M)$^+$or(M+2H)$^{2+}$ found |
|---|---|---|---|
| a | 2-6 | 1038.18 | 1038.1/519.6 |
| b | 1-5 | 1167.38 | 1167.2/584.2 |

The arrow indicates the cleavage site of trypsin digestion.

Ac-WGPenGGK↓GGPenGGGK↓GCGGK↓GGPenK↓CGWK↓GGCGW-$NH_2$

1 2 3 4 5 6

These fragments indicate the formation of 1-5, 2-6, 3-4

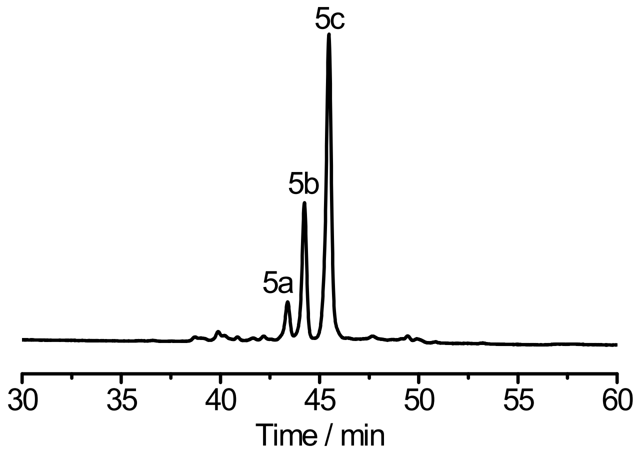


**Figure S11.** Chromatogram of the products formed after the oxidation of peptide **5**.

a)

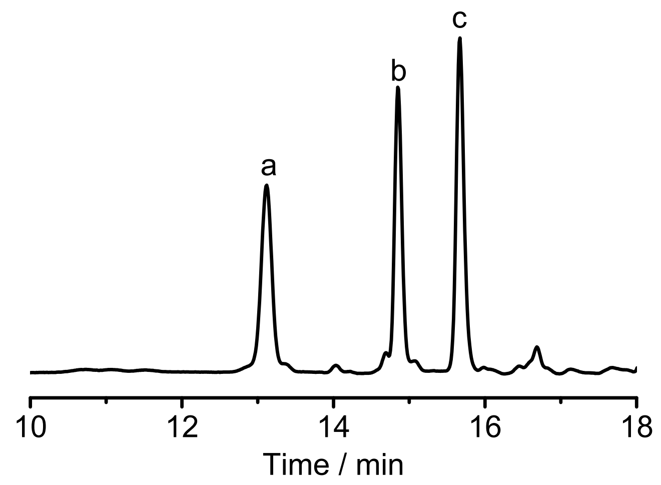


b)

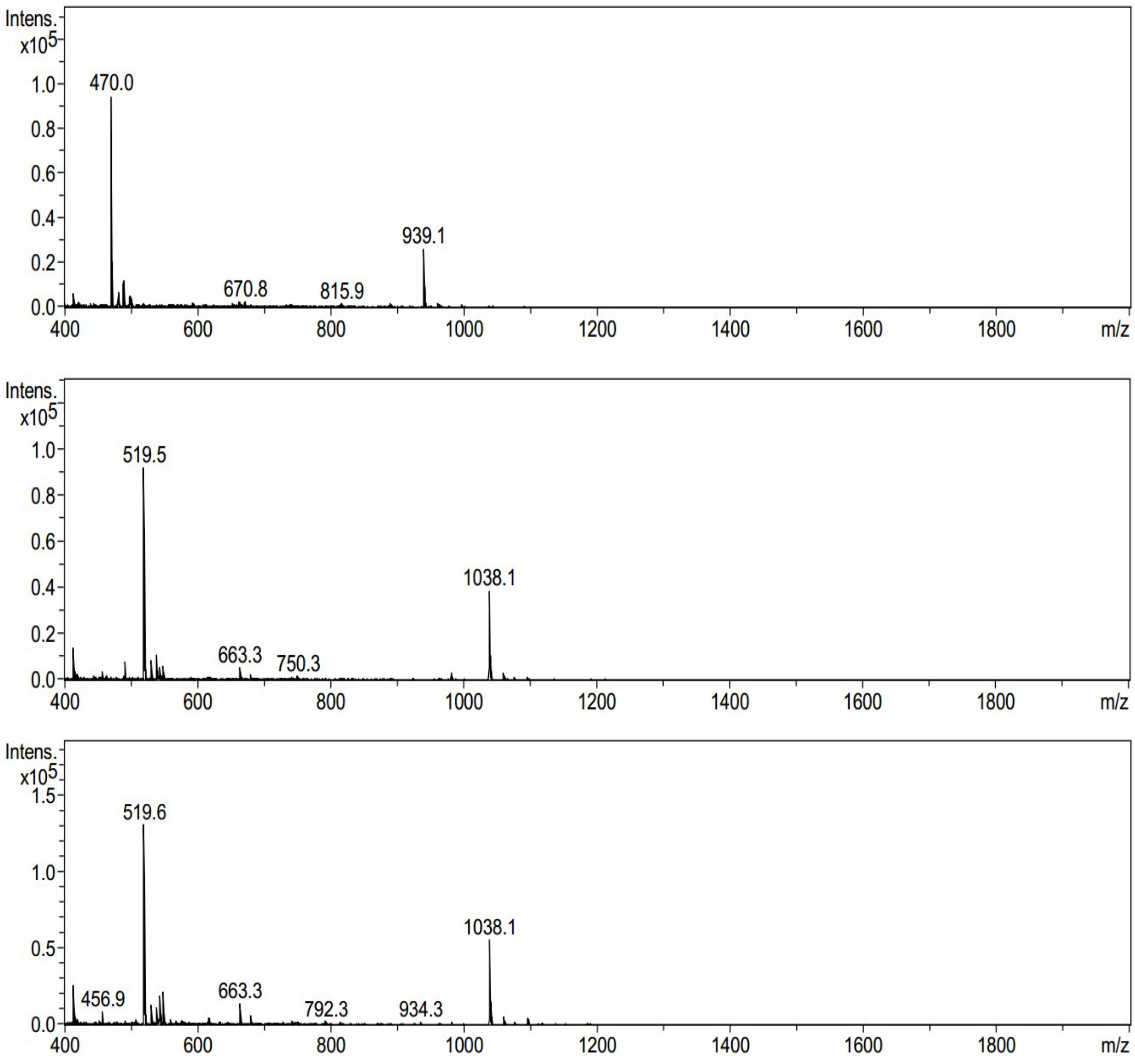


**Figure S12.** Tryptic digestion HPLC/MS analysis of **5a**: a) chromatogram of digested fragments from **5a**; b) mass spectra of fragments a-c (from top to bottom) labeled in the chromatogram.

**Fragment analysis:**

| Peak NO. | Disulfide pairing | $m/z$(M)$^{+}$ expected | $m/z$(M)$^{+}$or(M+2H)$^{2+}$ found |
|---|---|---|---|
| a | 3-5 | 939.13 | 939.1/470.0 |
| b | 2-6 | 1038.18 | 1038.1/519.5 |
| c | 1-4 | 1038.23 | 1038.1/519.6 |

The arrow indicates the cleavage site of trypsin digestion.

Ac-WGPenGGK↓GGPenGGGK↓GCGGK↓GGKC↓PenGWK↓GGCGW-$NH_2$

1 2 3 4 5 6

These fragments indicate the formation of 1-4, 2-6, 3-5.

Note that although fragments b and c had similar molecular weight values. *N*-terminally acetylated fragment with long retention time compare with other fragments. Thus, the disulfide pairing of fragment c is 1-4, fragment b is 2-5.

a)

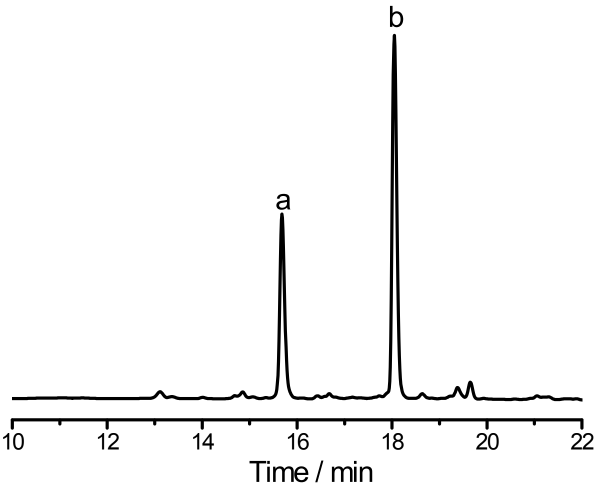


b)

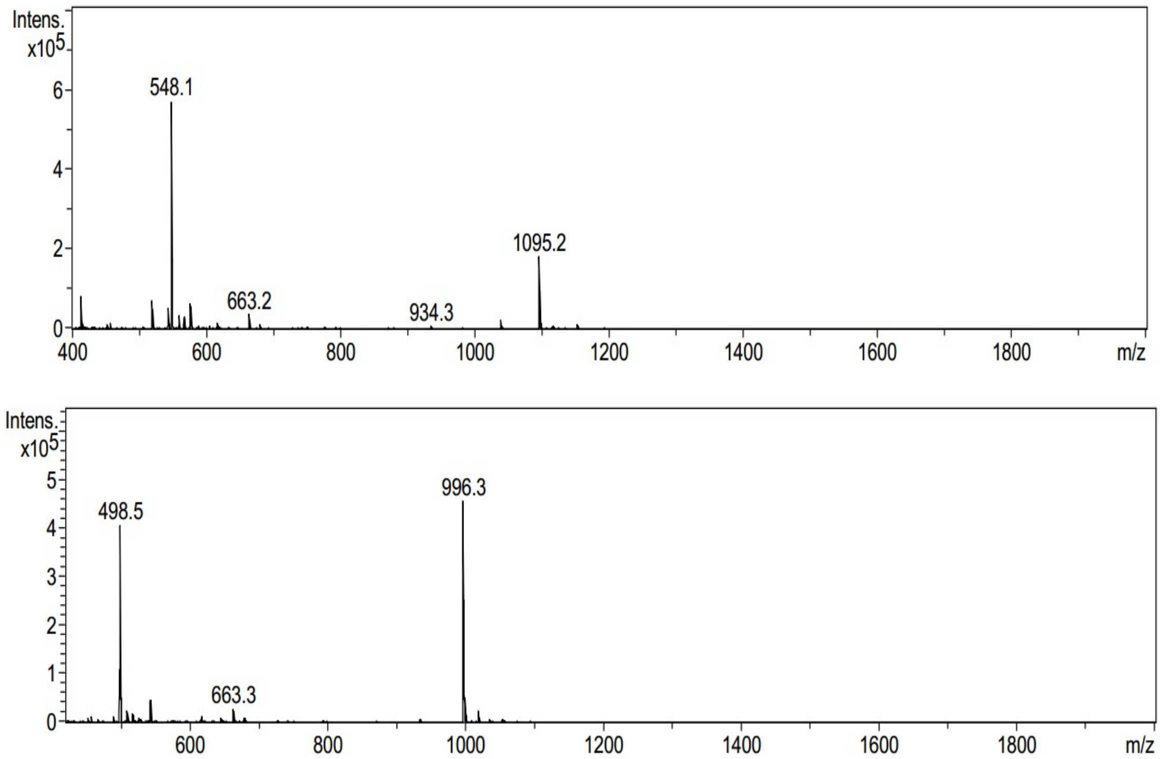


**Figure S13.** Tryptic digestion HPLC/MS analysis of **5b**: a) chromatogram of digested fragments from **5b**; b) mass spectra of fragments a and b labeled in the chromatogram.

**Fragment analysis:**

| Peak NO. | Disulfide pairing | *m/z*(M)$^+$ expected | *m/z*(M)$^+$or(M+2H)$^{2+}$ found |
|---|---|---|---|
| a | 1-3 | 1095.28 | 1095.2/548.1 |
| b | 5-6 | 996.18 | 996.3/498.5 |

The arrow indicates the cleavage site of trypsin digestion.

Ac-WGPenGGK↓GGPenGGGK↓GCGGK↓GGCK↓PenGWK↓GGCGW-$NH_2$

1 2 3 4 5 6

These fragments indicate the formation of 1-3, 2-4, 5-6.

a)

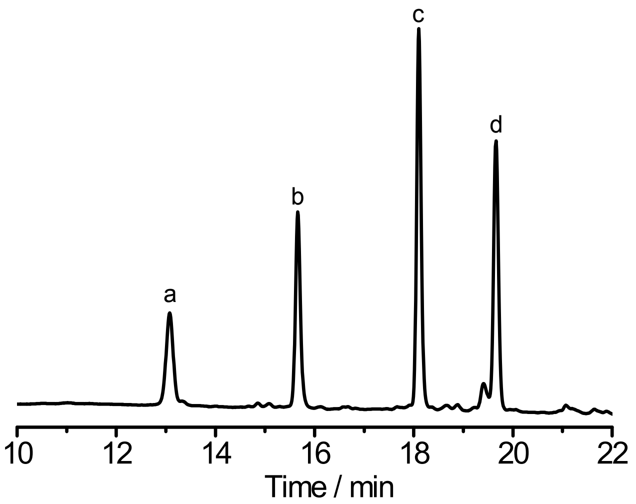
a
b
c
d
10
12
14
16
18
20
22
Time / min

b)

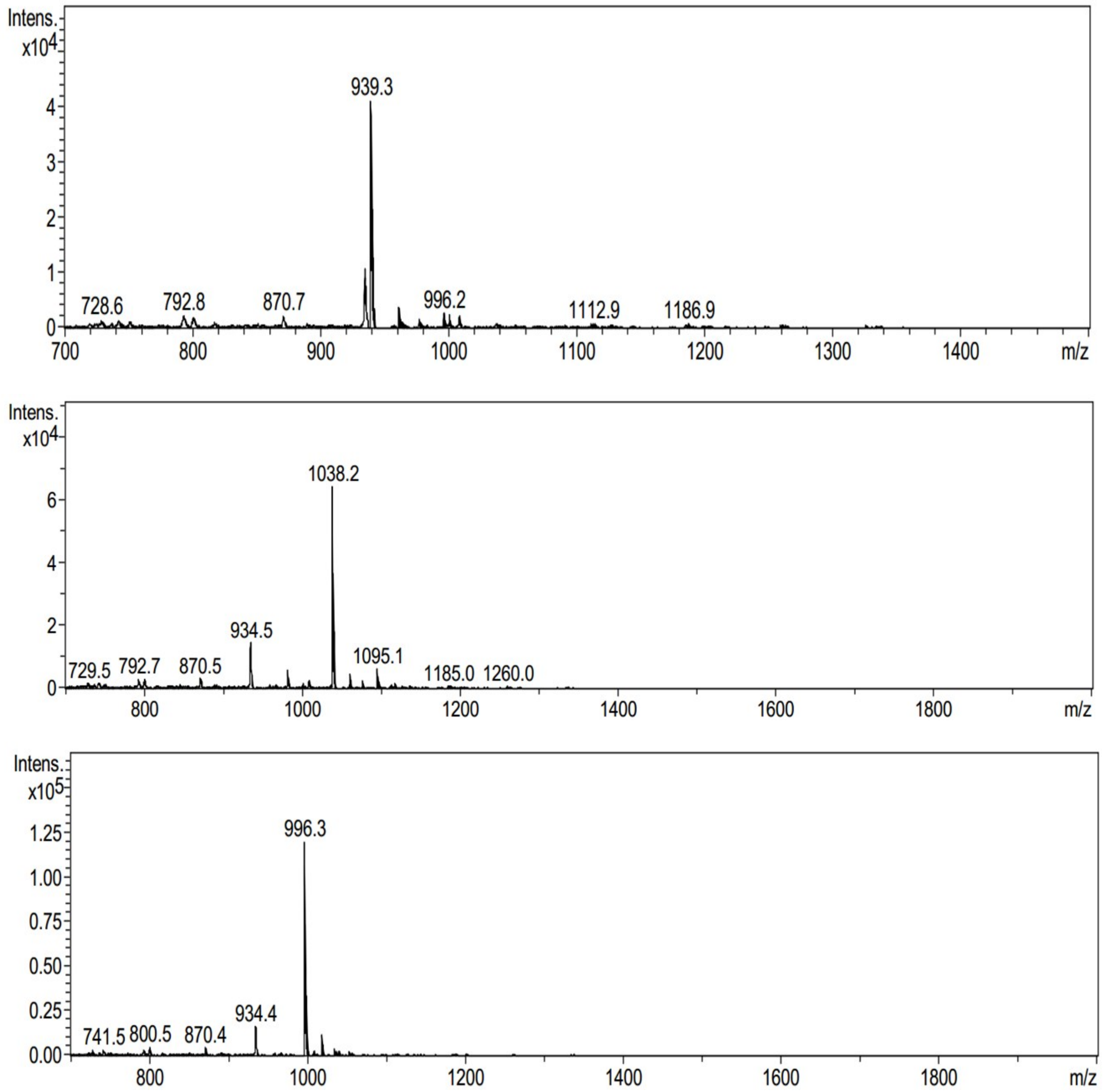
Intens.
x10^4
939.3
728.6
792.8
870.7
996.2
1112.9
1186.9
m/z
Intens.
x10^4
1038.2
934.5
729.5
792.7
870.5
1095.1
1185.0
1260.0
m/z
Intens.
x10^5
996.3
934.4
741.5
800.5
870.4
m/z

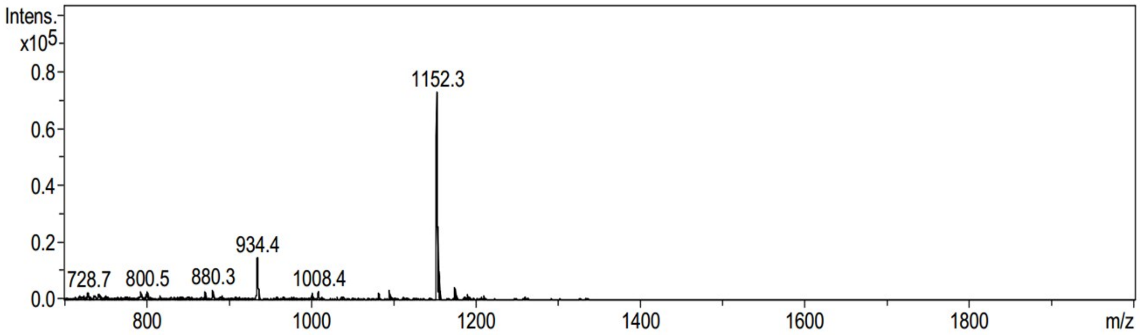


**Figure S14.** Tryptic digestion HPLC/MS analysis of **5c**: a) chromatogram of digested fragments from **5c**; b) mass spectra of fragments a-d labeled in the chromatogram.

**Fragment analysis:**

| Peak NO. | Disulfide pairing | *m/z*(M+H)$^+$ expected | *m/z*(M+H)$^+$ found |
|---|---|---|---|
| a | 3-5 | 939.13 | 939.3 |
| b | 1-4 | 1038.23 | 1038.2 |
| c | 5-6 | 996.1 | 996.3 |
| d | 1-6 | 1152.33 | 1152.3 |

The arrow indicates the cleavage site of trypsin digestion.

Ac-WGPenGGK↓GGPenGGGK↓GCGGK↓GGCK↓PenGWK↓GGCGW-$NH_2$
1 2 3 4 5 6

Fragments b and c indicate the formation of 1-4, 2-3, 5-6

Fragments a and d indicate the formation of 1-6, 2-4, 3-5.

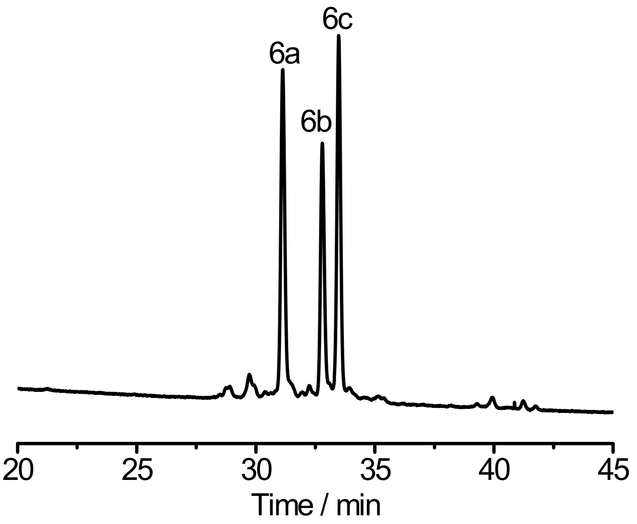


**Figure S15.** Chromatogram of the products formed after the oxidation of peptide **6**.

a)

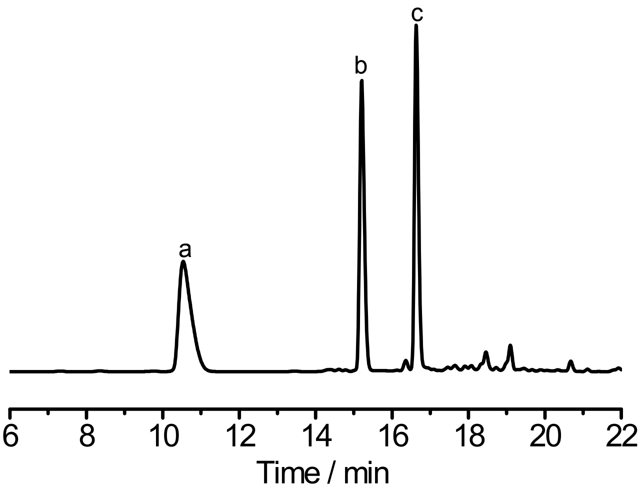


b)

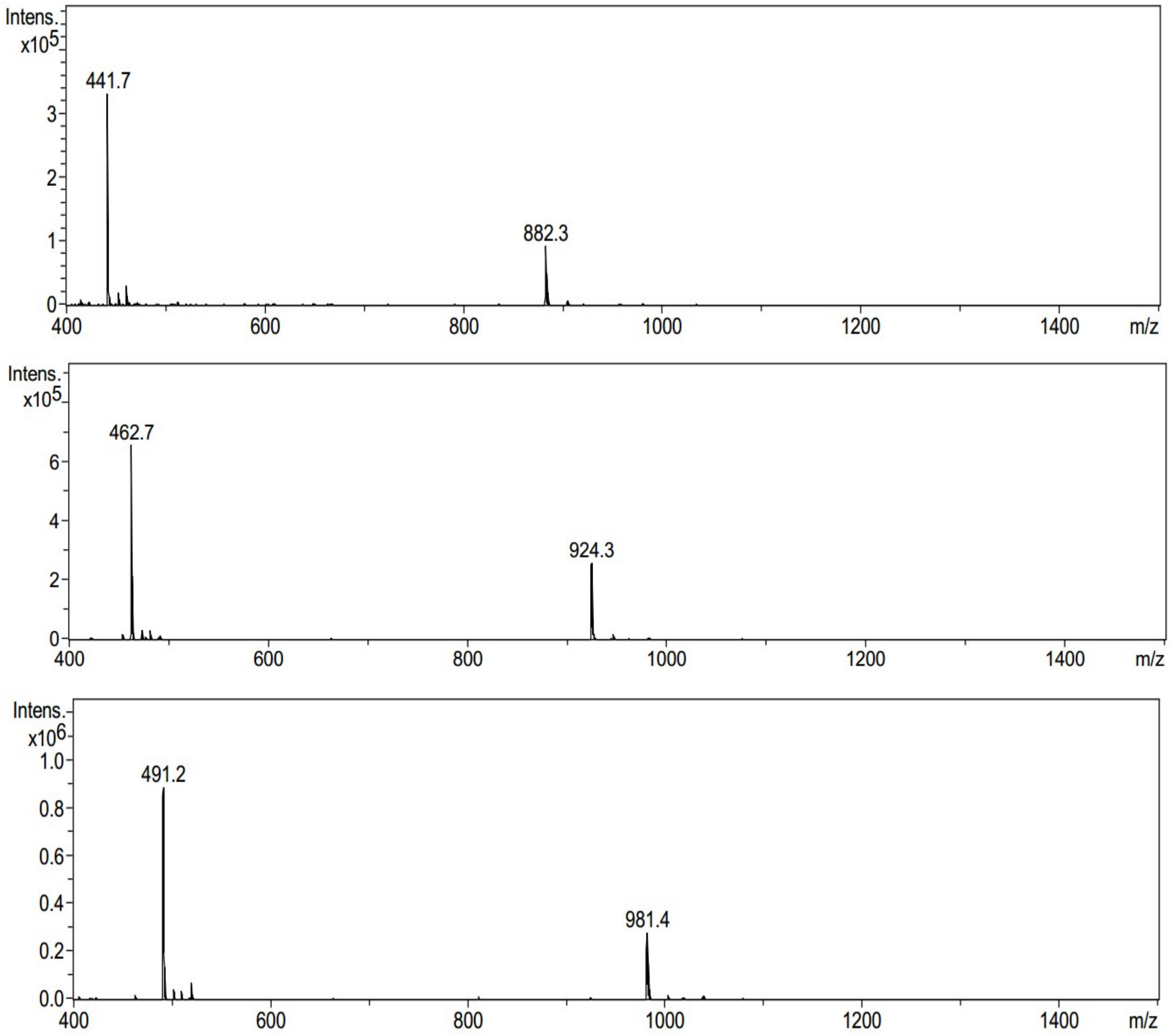


**Figure S16.** Tryptic digestion HPLC/MS analysis of **6a**: a) chromatogram of digested fragments from **6a**; b) mass spectra of fragments a-c labeled in the chromatogram.

**Fragment analysis:**

| Peak NO. | Disulfide pairing | *m/z*(M)$^+$ expected | *m/z*(M)$^+$ or(M+2H)$^{2+}$ found |
|---|---|---|---|
| a | 2-5 | 882.09 | 882.3/441.7 |
| b | 3-6 | 924.03 | 924.3/462.7 |
| c | 1-4 | 981.18 | 981.4/491.2 |

The arrow indicates the cleavage site of trypsin digestion.

Ac-WGCKPenGG K↓GGCGK↓GGGPenK↓CGWK↓GGPenGW-$NH_2$

1 2 3 4 5 6

These fragments indicate the formation of 1-4, 2-5, 3-6.

a)

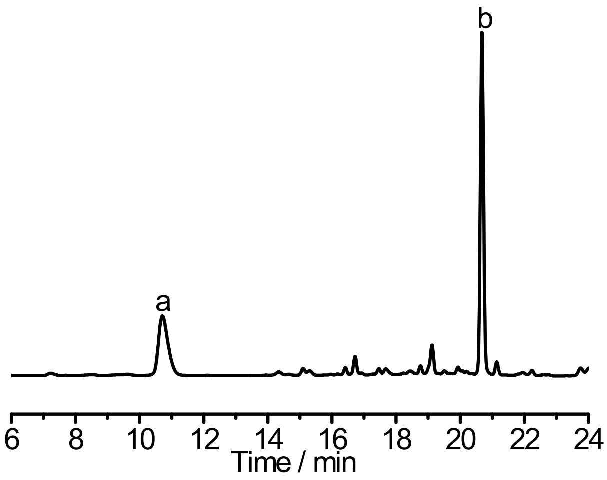


b)

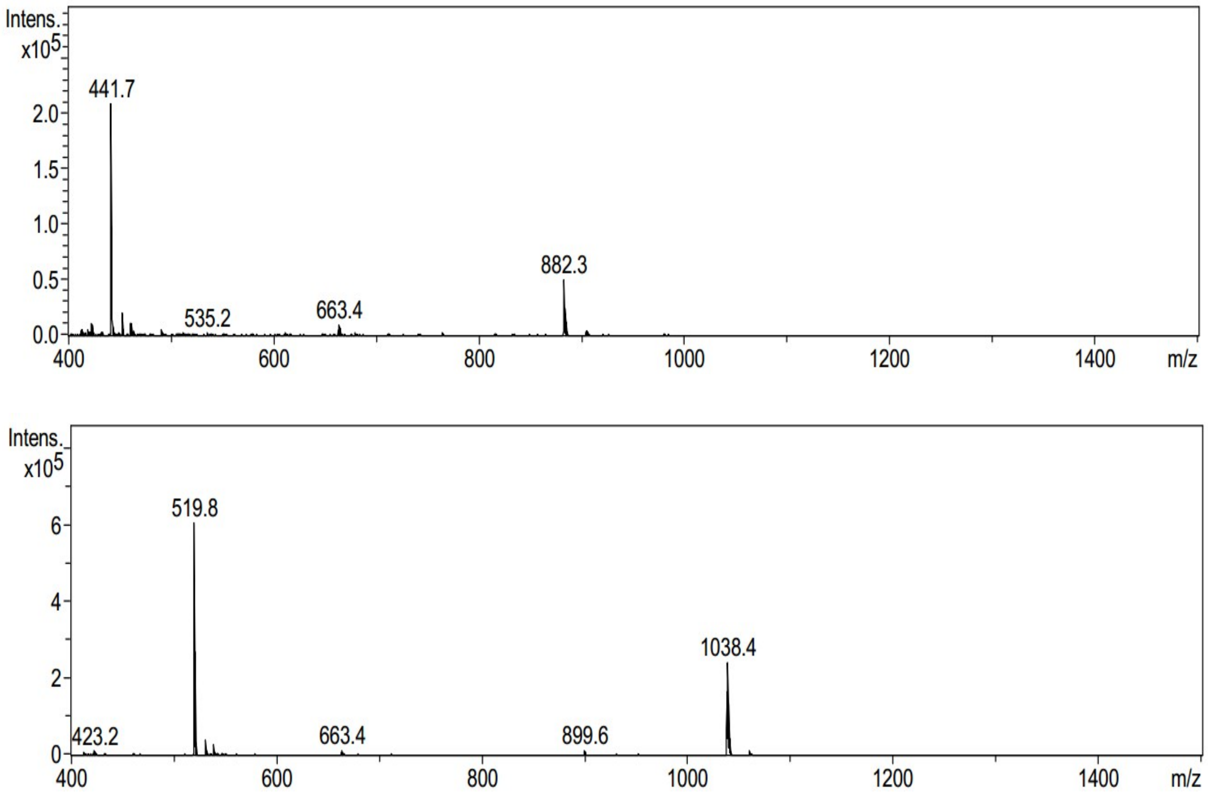


**Figure S17.** Tryptic digestion HPLC/MS analysis of **6b**: a) chromatogram of digested fragments from **6b**; b) mass spectra of fragments a and b labeled in the chromatogram.

**Fragment analysis:**

| **Peak NO.** | **Disulfide pairing** | ***m/z*(M)$^+$ expected** | ***m/z*(M)$^+$ or(M+2H)$^{2+}$ found** |
|---|---|---|---|
| a | 2-5 | 882.09 | 882.3/441.7 |
| b | 1-6 | 1038.18 | 1038.4/519.8 |

The arrow indicates the cleavage site of trypsin digestion.

Ac-WGCKPenGG K↓GGCGK↓GGGPenK↓CGWK↓GGPenGW-$NH_2$

1 2 3 4 5 6

These fragments indicate the formation of 1-6, 2-5, 3-4

a)

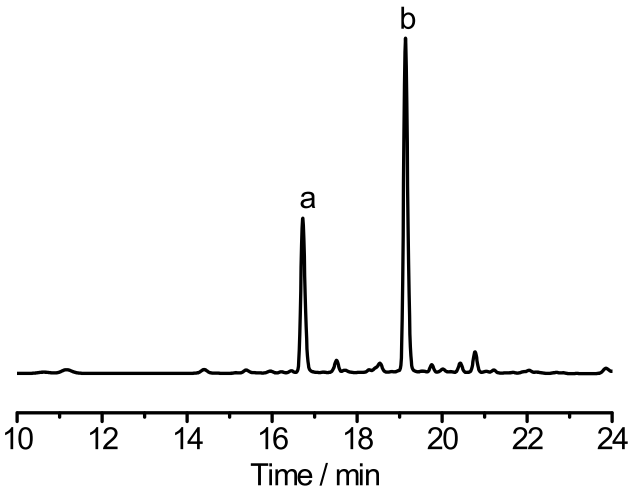


b)

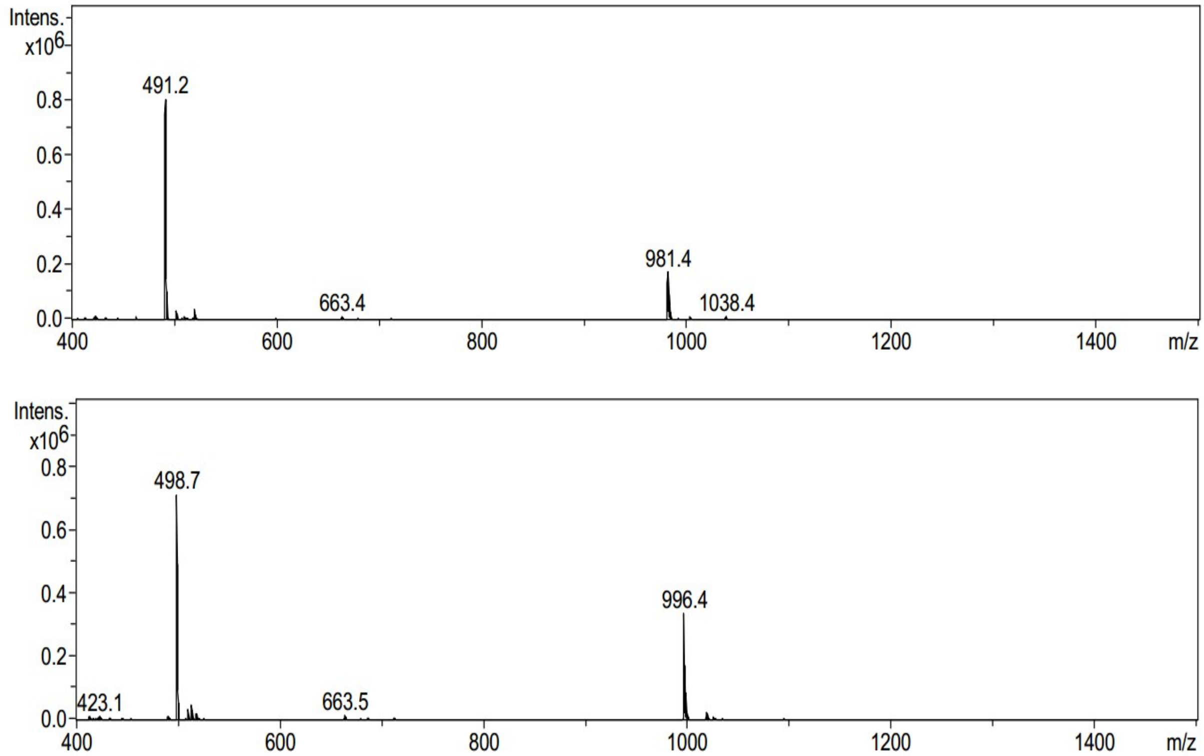


**Figure S18.** Tryptic digestion HPLC/MS analysis of **6c**: a) chromatogram of digested fragments from **6c**; b) mass spectra of fragments a and b labeled in the chromatogram.

**Fragment analysis:**

| Peak NO. | Disulfide pairing | ***m/z*(M)$^+$ expected** | ***m/z*(M)$^+$ or(M+2H)$^{2+}$ found** |
|---|---|---|---|
| a | 1-4 | 981.18 | 981.4/491.2 |
| b | 5-6 | 996.14 | 996.4/498.7 |

The arrow indicates the cleavage site of trypsin digestion.

Ac-WGCKPenGG K↓GGCGK↓GGGPenK↓CGWK↓GGPenGW-$NH_2$

1 2 3 4 5 6

These fragments indicate the formation of 1-4, 2-3, 5-6.

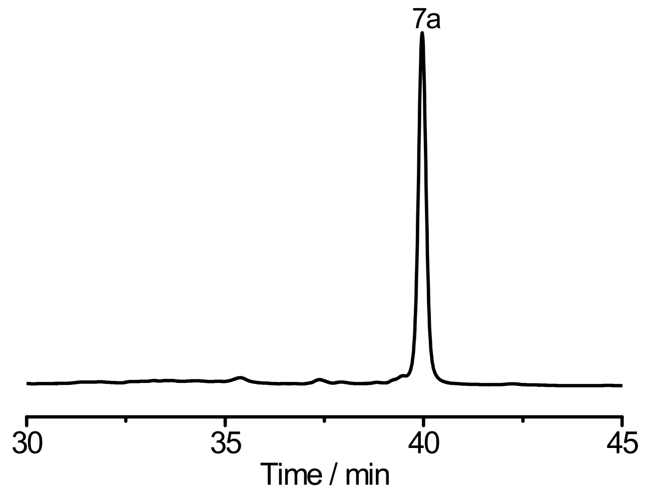


**Figure S19.** Chromatogram of the products formed after the oxidation of peptide **7**.

a)

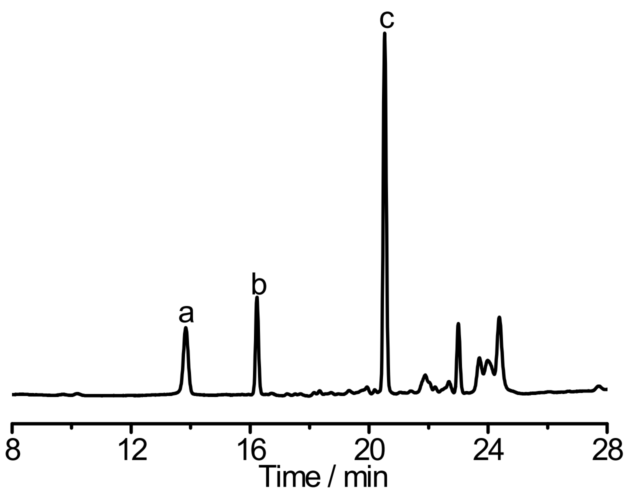


b)

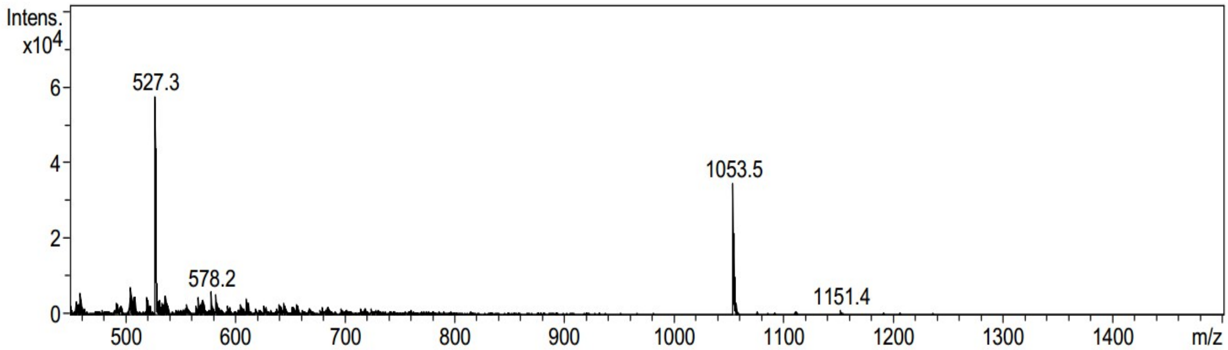


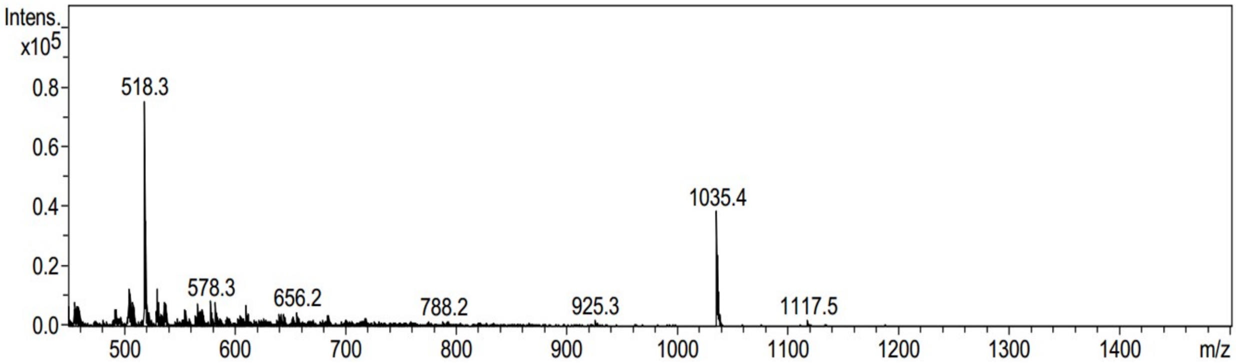


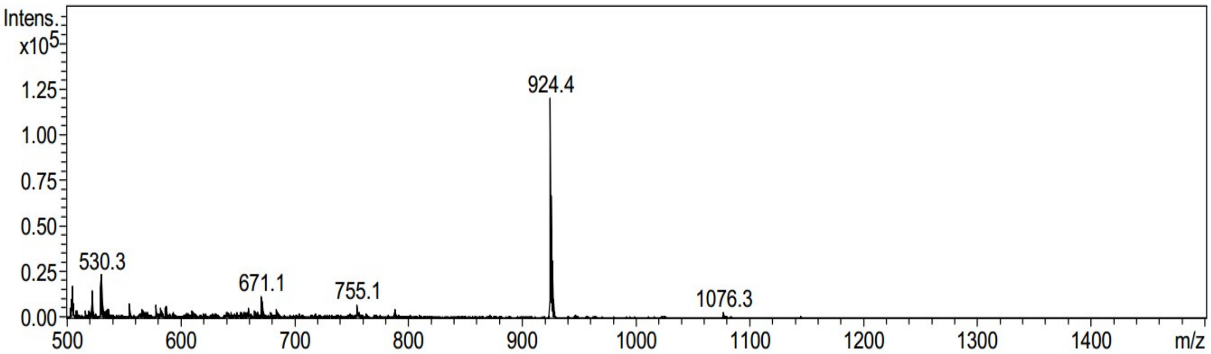


**Figure S20.** Tryptic digestion HPLC/MS analysis of **7a**: a) chromatogram of digested fragments from **7a**; b) mass spectra of fragments a-c labeled in the chromatogram.

**Fragment analysis:**

| Peak NO. | Disulfide pairing | *m/z*(M)$^+$ expected | *m/z*(M)$^+$ or(M+2H)$^{2+}$ found |
|---|---|---|---|
| a | 3-4 | 1053.24 | 1053.5/527.3 |
| b* | 3-4 | 1035.24 | 1035.4/518.3 |
| c | 1-6 | 924.13 | 924.4 |

The arrow indicates the cleavage site of trypsin digestion.

Ac-WGCKPenGGG K↓GCGG K↓GGPenGWK↓GGCK↓PenGW-$NH_2$

Ⅰ Ⅱ Ⅲ Ⅳ Ⅴ Ⅵ

These fragments indicate the formation of 1-6, 2-5, 3-4

b*: the cleavage site within the CGGKGGPenGWK fragment was not cleaved.

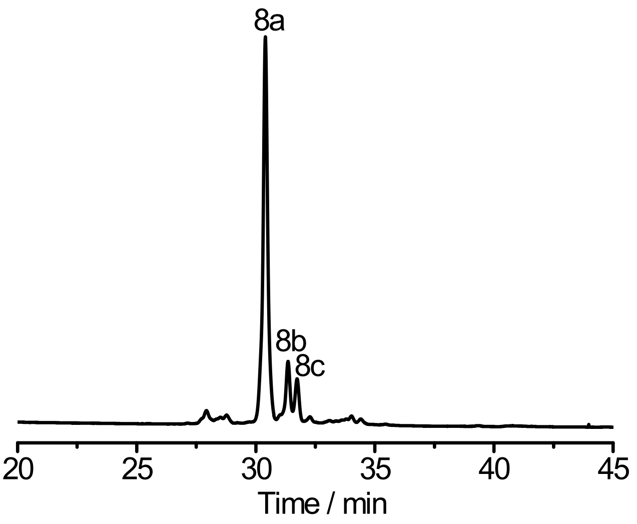


**Figure S21.** Chromatogram of the products formed after the oxidation of peptide **8**.

a)

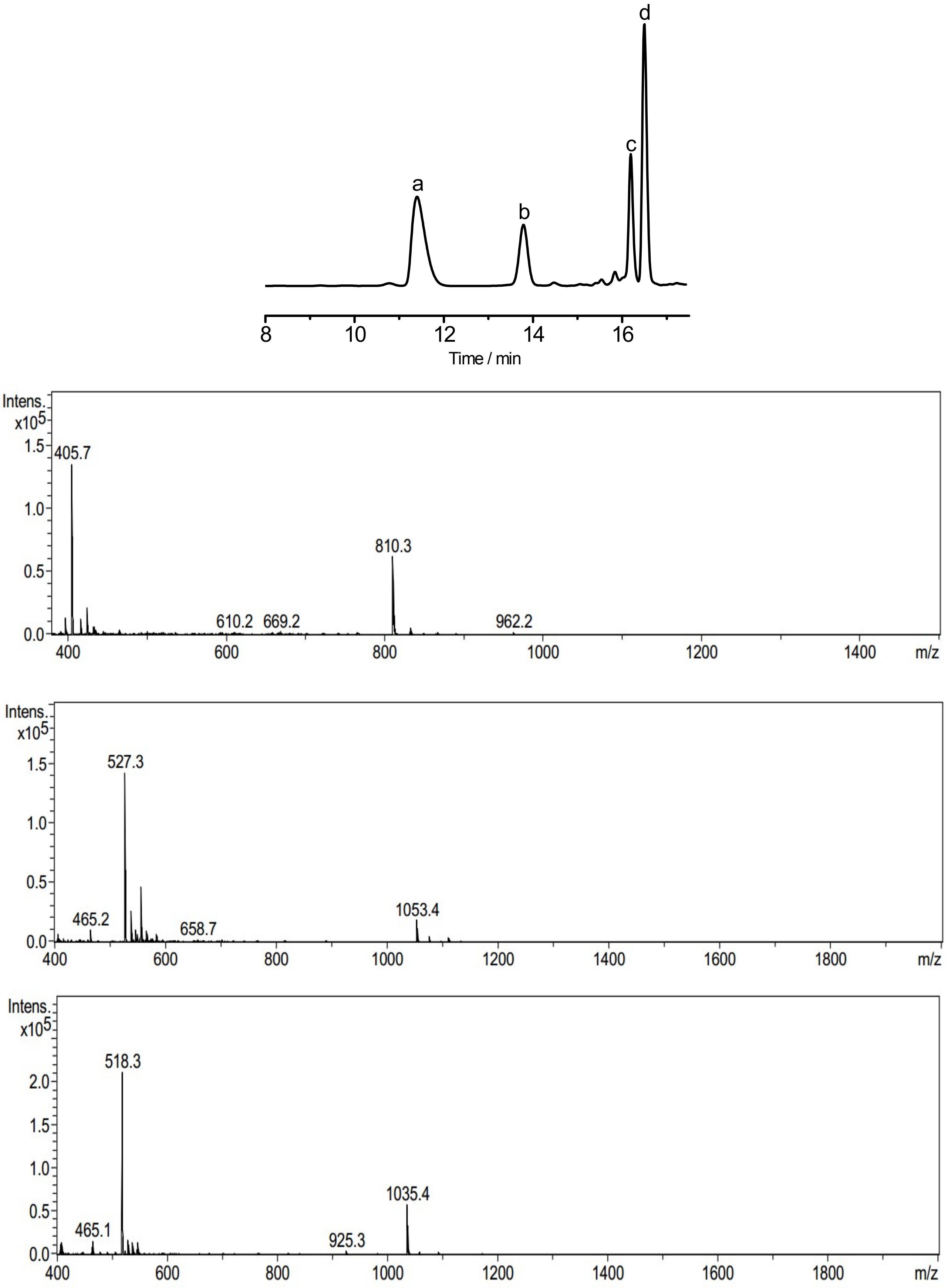

a
b
c
d
8
10
12
14
16
Time / min
Intens.
x10^5
1.5
1.0
0.5
0.0
405.7
810.3
610.2
669.2
962.2
400
600
800
1000
1200
1400
m/z
Intens.
x10^5
1.5
1.0
0.5
0.0
527.3
465.2
658.7
1053.4
400
600
800
1000
1200
1400
1600
1800
m/z
Intens.
x10^5
2.0
1.5
1.0
0.5
0.0
518.3
465.1
925.3
1035.4
400
600
800
1000
1200
1400
1600
1800
m/z

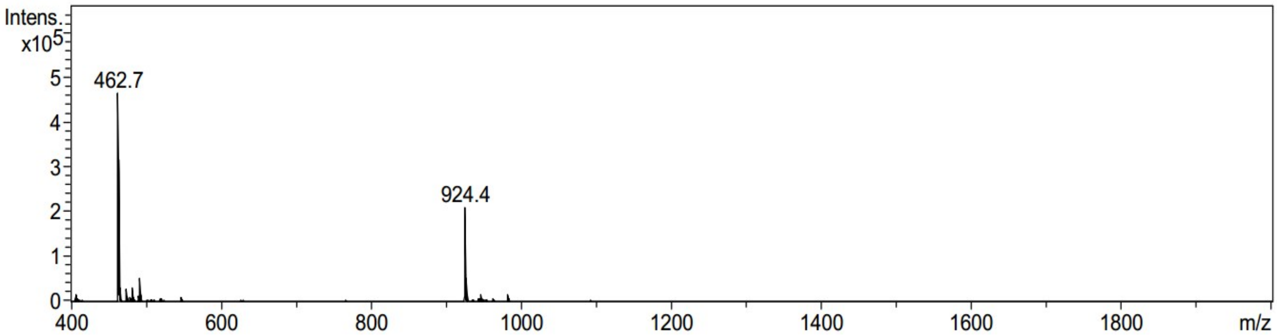


**Figure S22.** Tryptic digestion HPLC/MS analysis of **8a**: a) chromatogram of digested fragments from **8a**; b) mass spectra of fragments a-d labeled in the chromatogram.

**Fragment analysis:**

| **Peak NO.** | **Disulfide pairing** | ***m/z*(M)⁺ expected** | ***m/z*(M)⁺ or(M+2H)²⁺ found** |
|---|---|---|---|
| a | 2-6 | 809.98 | 810.3/405.99 |
| b | 3-4 | 1053.24 | 1053.4/527.3 |
| c* | 3-4 | 1035.24 | 1035.4/518.3 |
| d | 1-5 | 924.13 | 924.4/462.7 |

The arrow indicates the cleavage site of trypsin digestion.

Ac-WGCKPenGGG K↓GCGG K↓GGPenGWK↓GGPenK↓CGW-$NH_2$
1 2 3 4 5 6

These fragments indicate the formation of 1-5, 2-6, 3-4

c*: the cleavage site within the CGGKGGPenGWK fragment was not cleaved.

a)

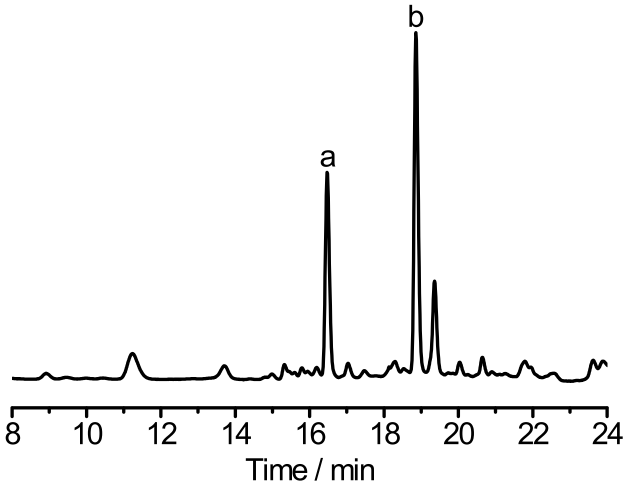


b)

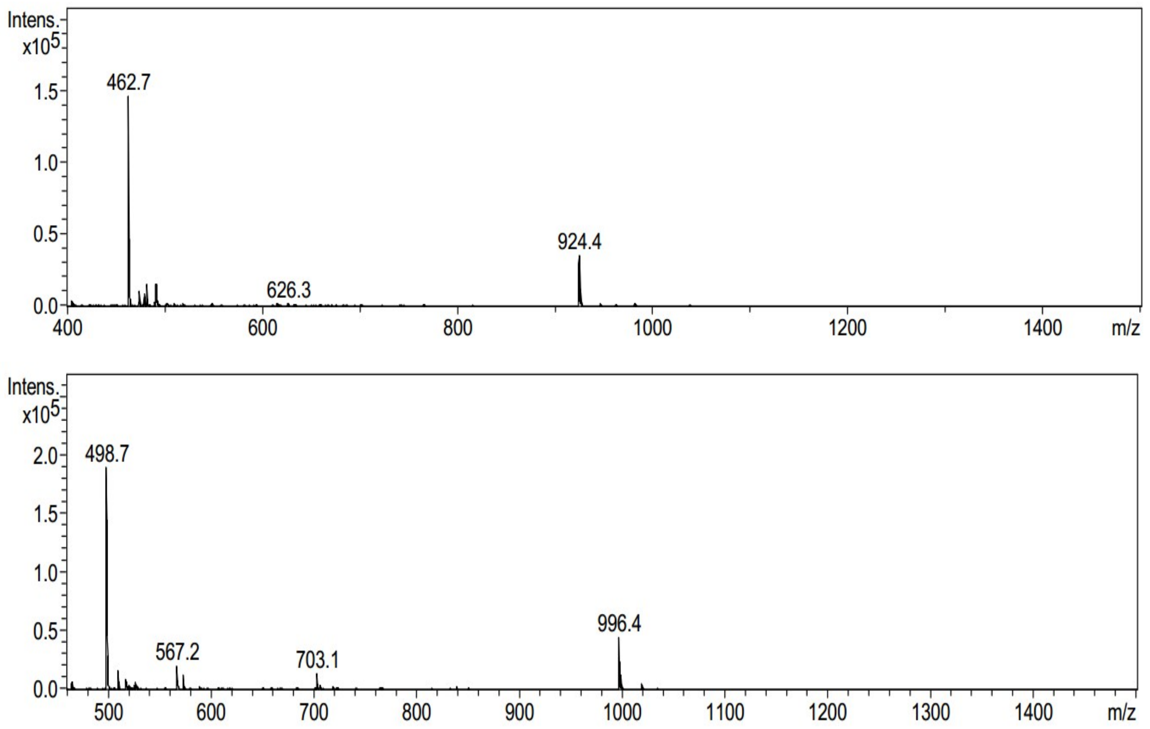


**Figure S23.** Tryptic digestion HPLC/MS analysis of **8b**: a) chromatogram of digested fragments from **8b**; b) mass spectra of fragments a and b labeled in the chromatogram.

**Fragment analysis:**

| Peak NO. | Disulfide pairing | *m/z*(M)$^+$ expected | *m/z*(M)$^+$ or(M+2H)$^{2+}$ found |
|---|---|---|---|
| a | 1-5 | 924.13 | 924.4/462.7 |
| b | 4-6 | 996.19 | 996.4/498.7 |

The arrow indicates the cleavage site of trypsin digestion.

Ac-WGCKPenGGG K↓GCGG K↓GGPenGWK↓GGPenK↓CGW-$NH_2$

1 2 3 4 5 6

These fragments indicate the formation of 1-5, 2-3, 4-6

a)

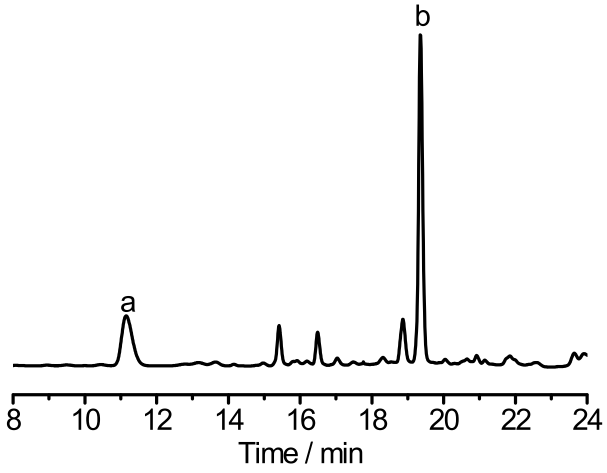


b)

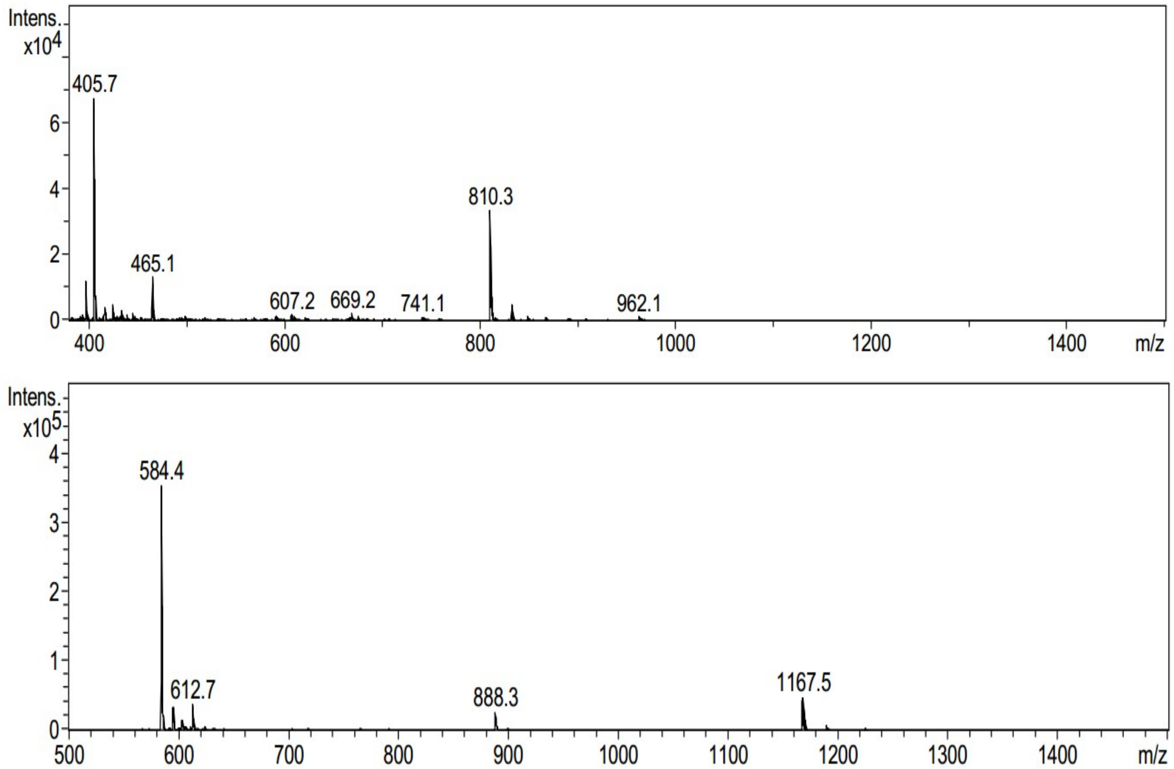


**Figure S24.** Tryptic digestion HPLC/MS analysis of **8c**: a) chromatogram of digested fragments from **8c**; b) mass spectra of fragments a and b labeled in the chromatogram.

**Fragment analysis:**

| Peak NO. | Disulfide pairing | *m/z*(M)$^+$ expected | *m/z*(M)$^+$ or(M+2H)$^{2+}$ found |
|---|---|---|---|
| a | 2-6 | 809.98 | 810.3/405.7 |
| b | 1-4 | 1167.39 | 1167.5/584.4 |

The arrow indicates the cleavage site of trypsin digestion.

Ac-WGCKPenGGG K↓GCGG K↓GGPenGWK↓GGPenK↓CGW-$NH_2$

1 2 3 4 5 6

These fragments indicate the formation of 1-4, 2-6, 3-5

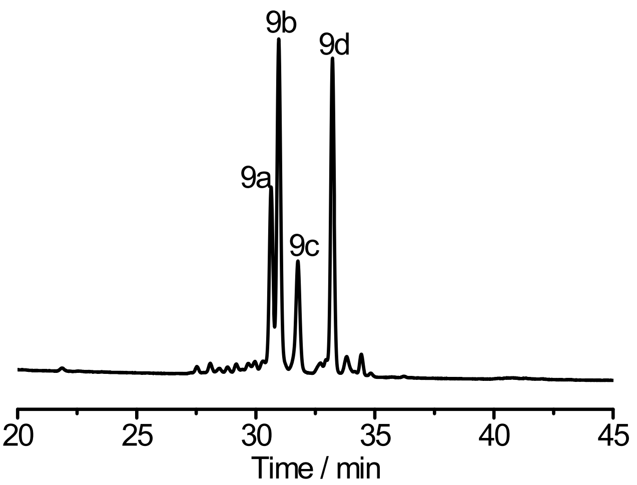


**Figure S25.** Chromatogram of the products formed after the oxidation of peptide **9**.

a)

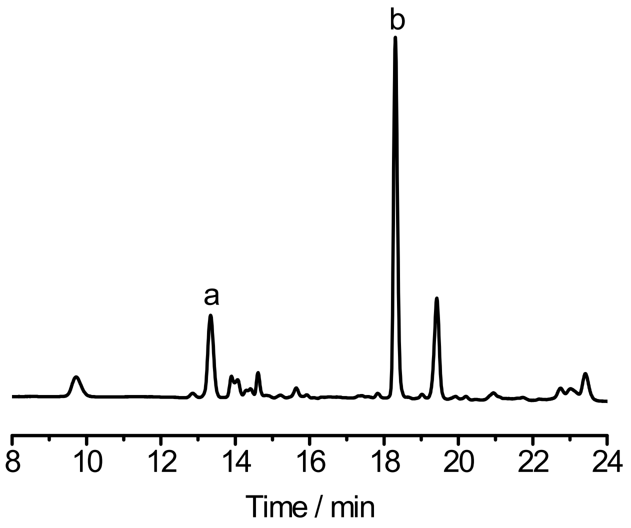


b)

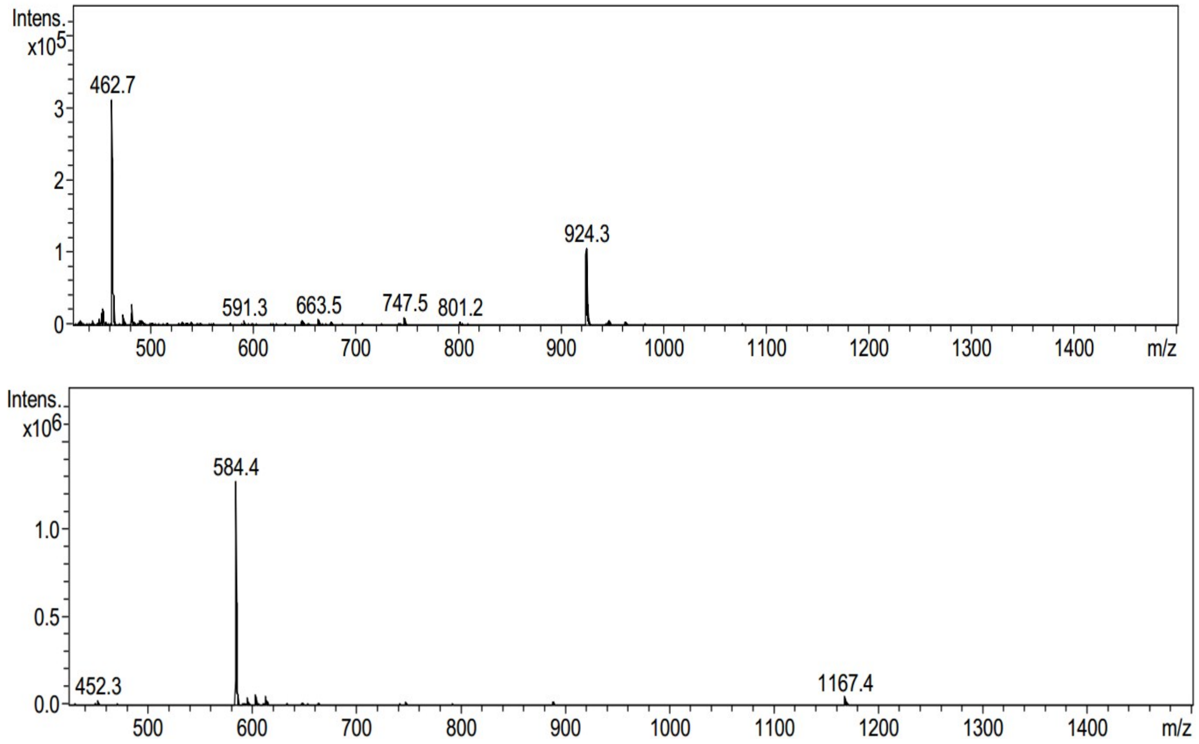


**Figure S26.** Tryptic digestion HPLC/MS analysis of **9a**: a) chromatogram of digested fragments from **9a**; b) mass spectra of fragments a and b labeled in the chromatogram.

**Fragment analysis:**

| Peak NO. | Disulfide pairing | *m/z*(M)$^+$ expected | *m/z*(M)$^+$ or(M+2H)$^{2+}$ found |
|---|---|---|---|
| a | 3-6 | 924.01 | 924.3/462.7 |
| b | 1-4 | 1167.39 | 1167.4/584.4 |

The arrow indicates the cleavage site of trypsin digestion.

Ac-WGCGGK↓GGPenK↓CGGGK↓GCK↓PenGWK↓GGPenGW-$NH_2$

1 2 3 4 5 6

These fragments indicate the formation of 1-4, 2-5, 3-6

a)

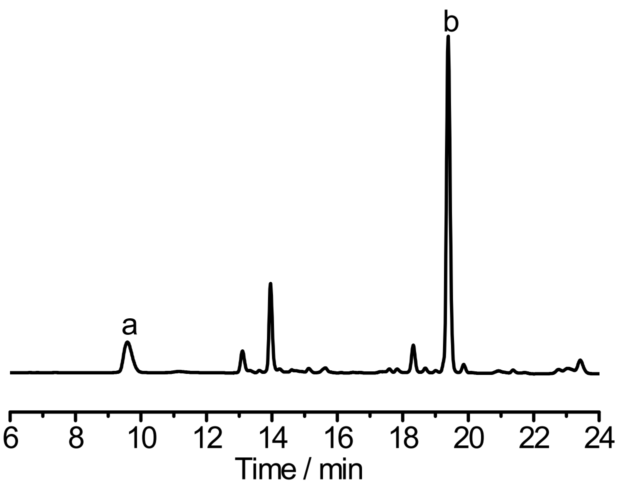


a)

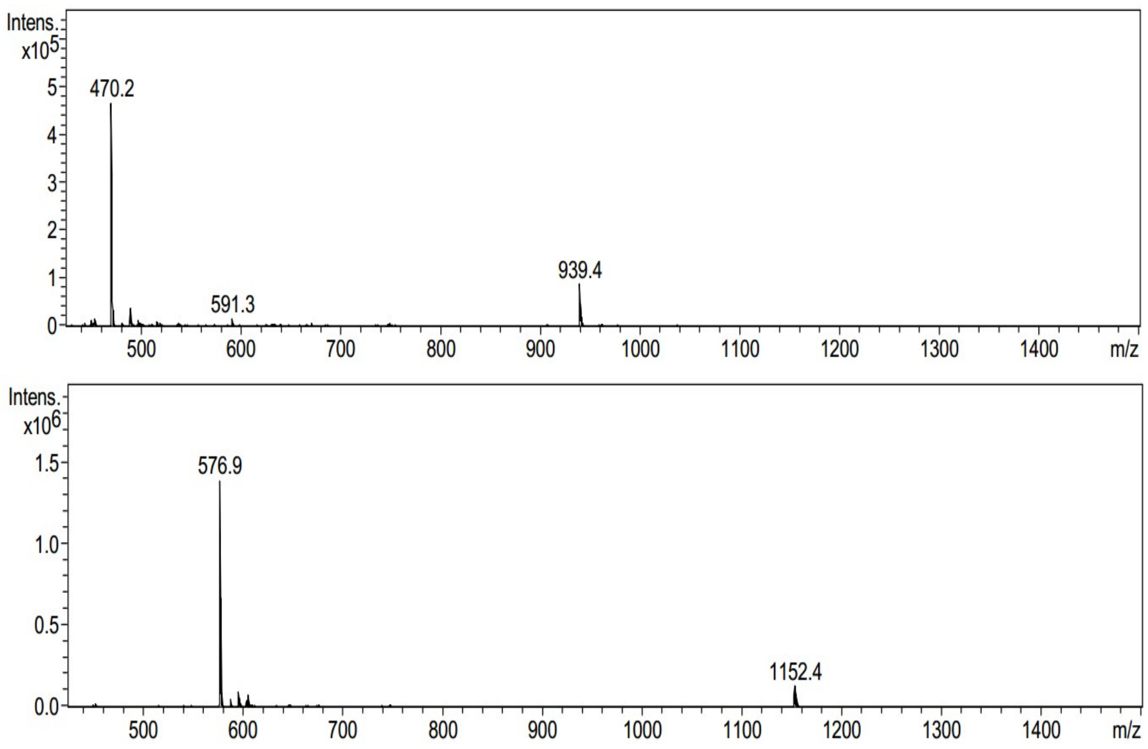


**Figure S27.** Tryptic digestion HPLC/MS analysis of **9b**: a) chromatogram of digested fragments from **9b**; b) mass spectra of fragments a and b labeled in the chromatogram.

**Fragment analysis:**

| Peak NO. | Disulfide pairing | *m/z*(M)$^+$ expected | *m/z*(M)$^+$ or(M+2H)$^{2+}$ found |
|---|---|---|---|
| a | 3-5 | 939.07 | 939.4/470.2 |
| b | 1-6 | 1152.33 | 1152.4/576.9 |

The arrow indicates the cleavage site of trypsin digestion.

Ac-WGCGGK↓GGPenK↓CGGGK↓GCK↓PenGWK↓GGPenGW-$NH_2$

1 2 3 4 5 6

These fragments indicate the formation of 1-6, 2-4, 3-5

a)

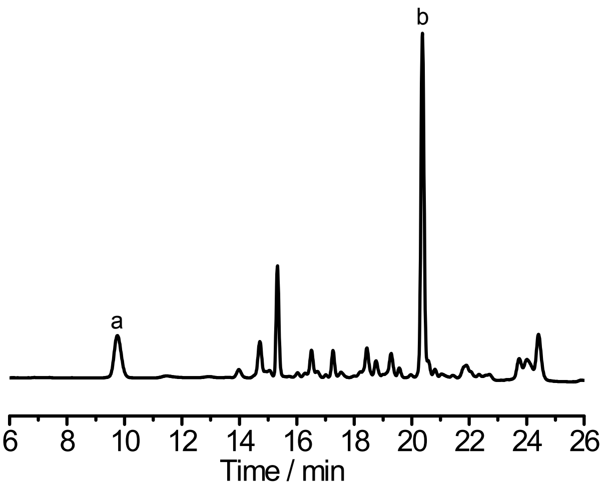


b)

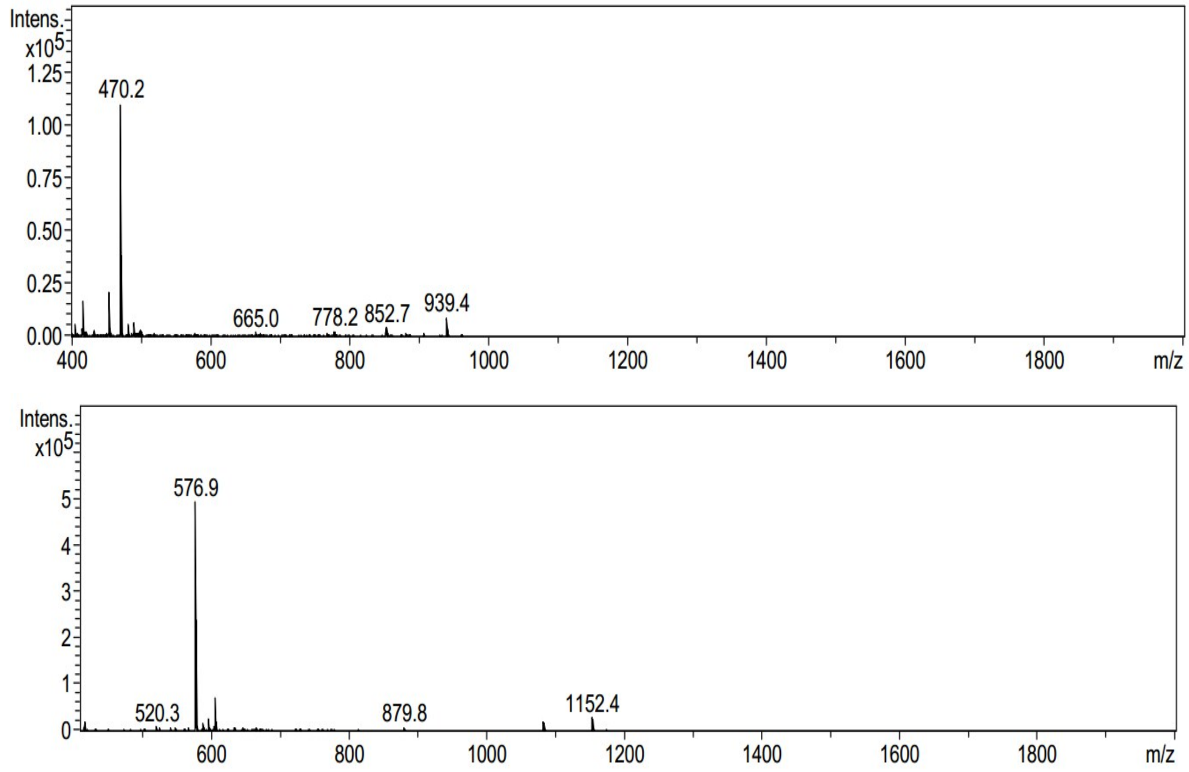


**Figure S28.** Tryptic digestion HPLC/MS analysis of **9c**: a) chromatogram of digested fragments from **9c**; b) mass spectra of fragments a and b labeled in the chromatogram.

**Fragment analysis:**

| Peak NO. | Disulfide pairing | *m/z*(M)$^+$ expected | *m/z*(M)$^+$ or(M+2H)$^{2+}$ found |
|---|---|---|---|
| a | 3-5 | 939.07 | 939.4/470.2 |
| b | 1-6 | 1152.33 | 1152.4/576.9 |

The arrow indicates the cleavage site of trypsin digestion.

Ac-WGCGGK↓GGPenK↓CGGGK↓GCK↓PenGWK↓GGPenGW-$NH_2$

1 2 3 4 5 6

These fragments indicate the formation of 1-6, 2-4, 3-5

a)

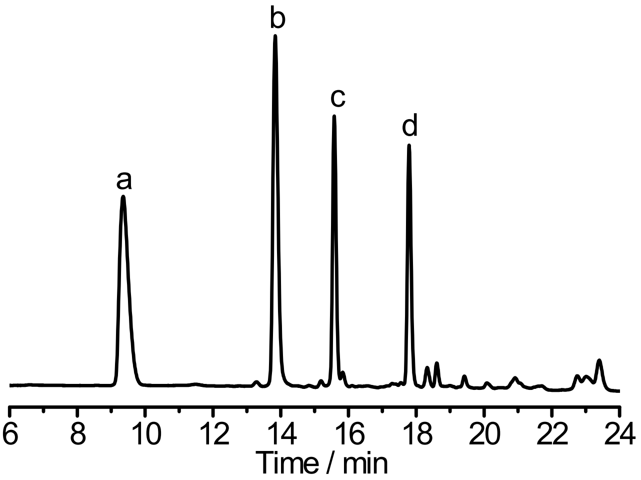

a
b
c
d
6
8
10
12
14
16
18
20
22
24
Time / min


b)

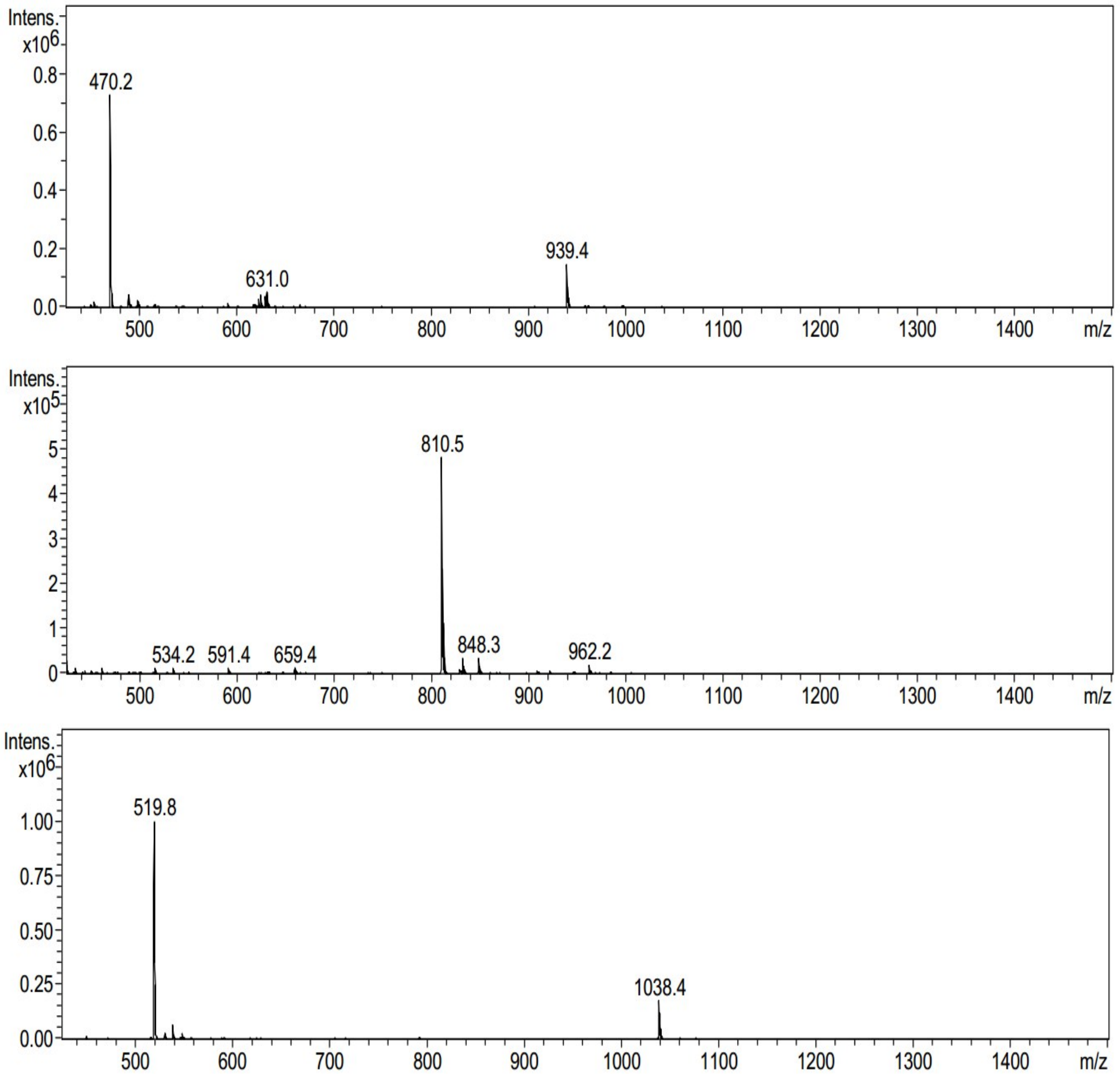

Intens.
x10^6
0.8
0.6
0.4
0.2
0.0
470.2
631.0
939.4
m/z
Intens.
x10^5
810.5
534.2
591.4
659.4
848.3
962.2
m/z
Intens.
x10^6
1.00
0.75
0.50
0.25
0.00
519.8
1038.4
500
600
700
800
900
1000
1100
1200
1300
1400
m/z

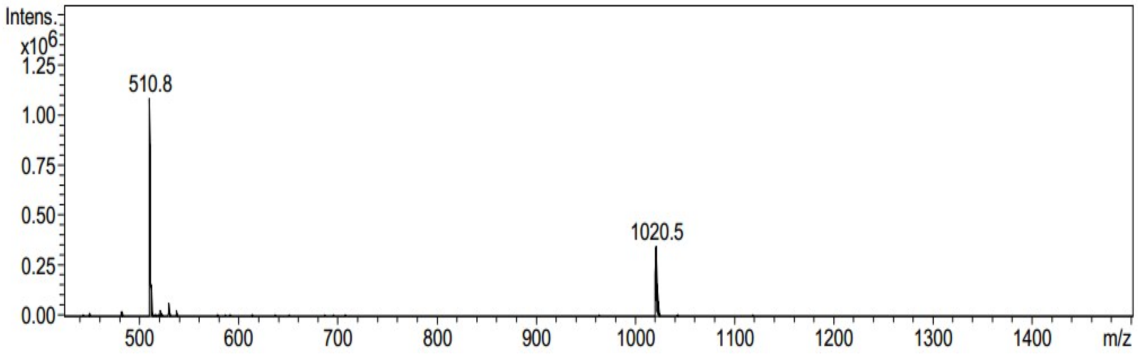


**Figure S29.** Tryptic digestion HPLC/MS analysis of **9d**: a) chromatogram of digested fragments from **9d**; b) mass spectra of fragments a-d labeled in the chromatogram.

**Fragment analysis:**

| **Peak NO.** | **Disulfide pairing** | ***m/z*(M)$^+$ expected** | ***m/z*(M)$^+$ or (M+2H)$^{2+}$ found** |
|---|---|---|---|
| a | 3-5 | 939.07 | 939.4/470.2 |
| b | 4-6 | 809.98 | 810.5 |
| c | 1-2 | 1038.23 | 1038.4/519.8 |
| d* | 1-2 | 1020.23 | 1020.5/510.8 |

The arrow indicates the cleavage site of trypsin digestion.

Ac-WGCGGK↓GGPenK↓CGGGK↓GCK↓PenGWK↓GGPenGW-$NH_2$
1 2 3 4 5 6

These fragments indicate the formation of 1-2, 3-5, 4-6

d*: the cleavage site within the Ac-WGCGGKGGPenK fragment was not cleaved.

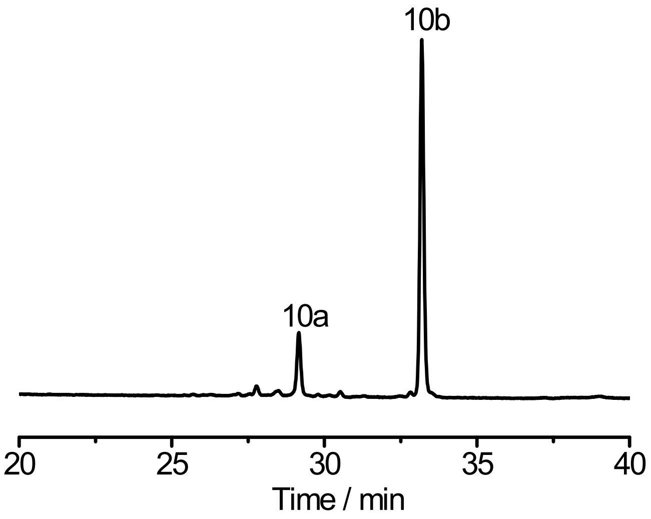


**Figure S30.** Chromatogram of the products formed after the oxidation of peptide **10**.

a)

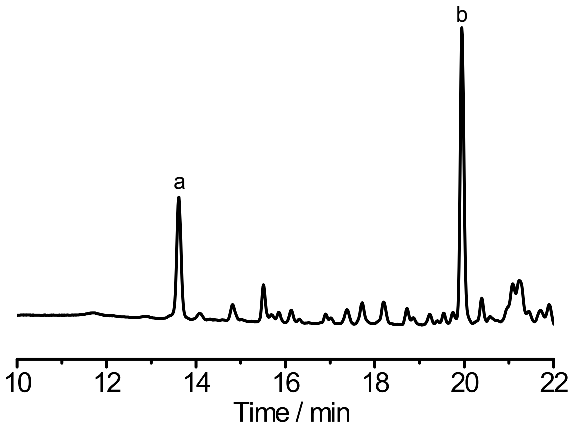


b)

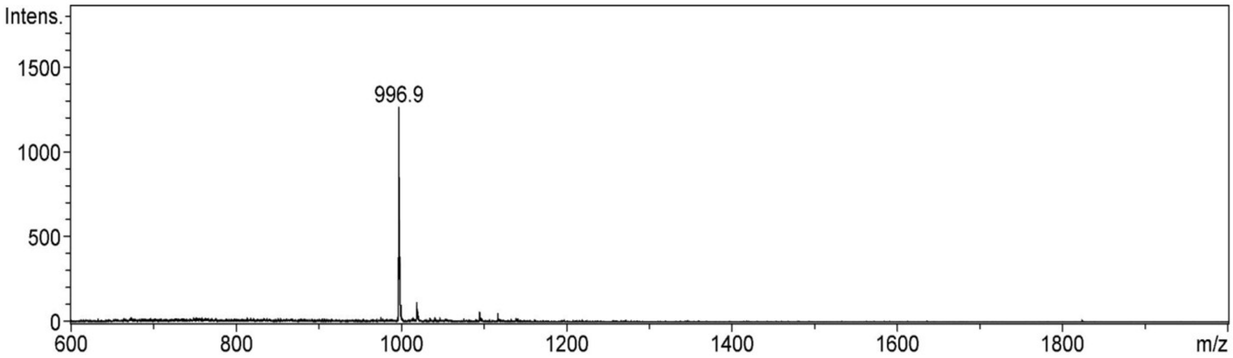


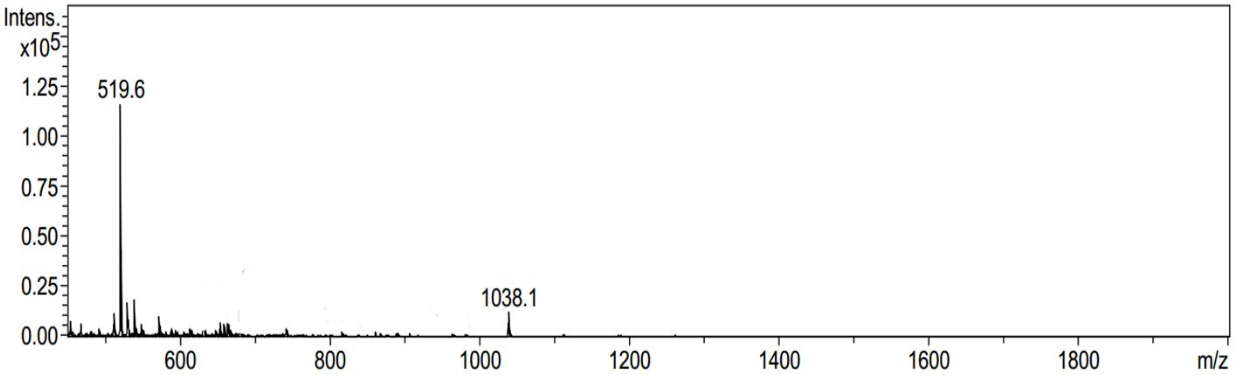


**Figure S31.** Tryptic digestion HPLC/MS analysis of **10a**: a) chromatogram of digested fragments from **10a**; b) mass spectra of fragments a and b labeled in the chromatogram.

**Fragment analysis:**

| Peak NO. | Disulfide pairing | *m/z*(M)$^+$ expected | *m/z*(M)$^+$ found |
|---|---|---|---|
| a | 4-5 | 996.15 | 996.9 |
| b | 1-6 | 1040.18 | 1040.6 |

The arrow indicates the cleavage site of trypsin digestion.

Ac-WGCK↓PenGGK↓GGCKPen↓GK↓GGGCGWK↓GGPenGW-$NH_2$

1 2 3 4 5 6

These fragments indicate the formation of 1-6, 2-3, 4-5

a)

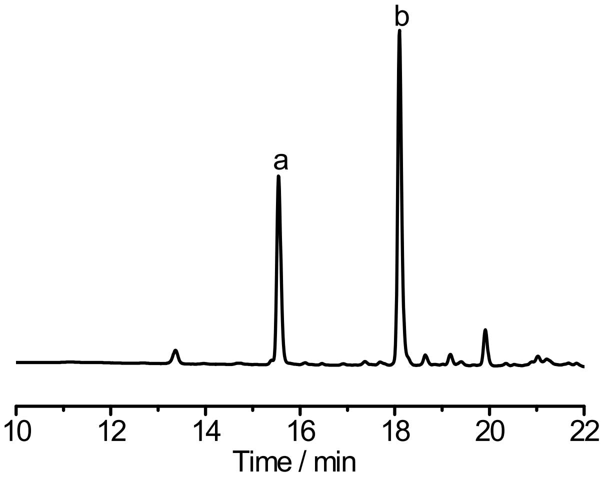


b)

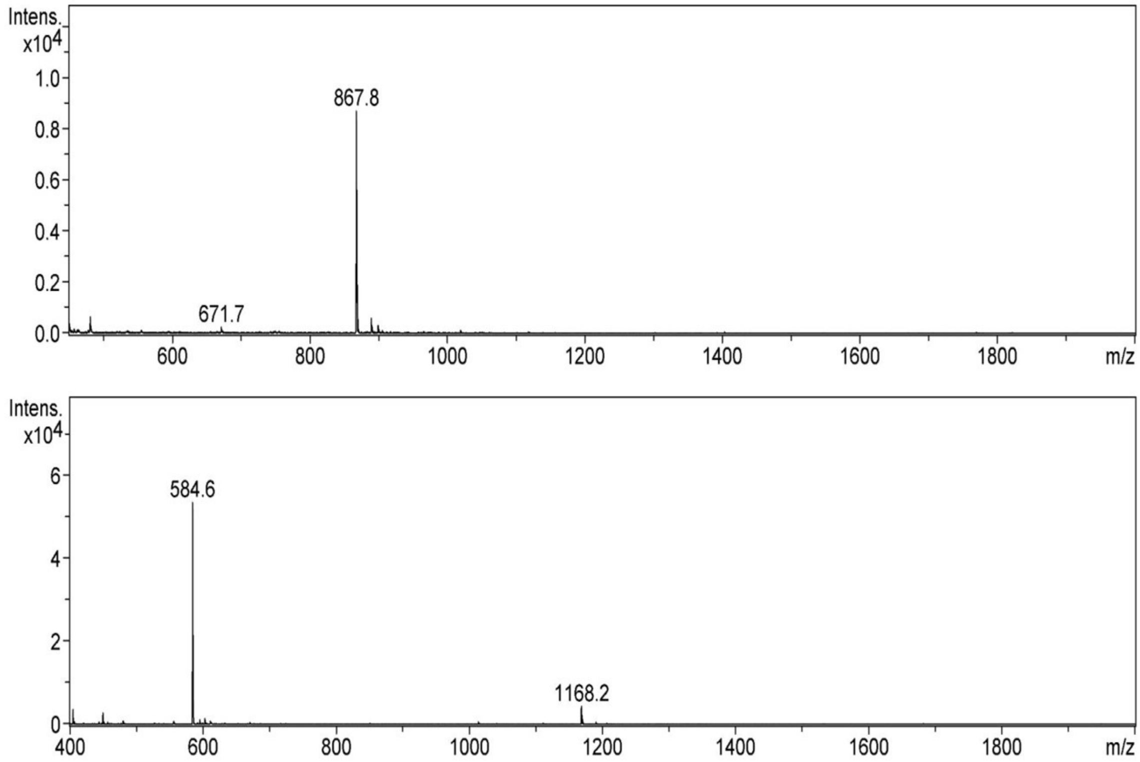


**Figure S32.** Tryptic digestion HPLC/MS analysis of **10b**: a) chromatogram of digested fragments from **10b**; b) mass spectra of fragments a-b labeled in the chromatogram.

**Fragment analysis:**

| **Peak NO.** | **Disulfide pairing** | ***m/z*(M)$^+$ expected** | ***m/z*(M)$^+$ or (M+2H)$^{2+}$ found** |
|---|---|---|---|
| a | 1-4 | 867.03 | 867.8 |
| b | 5-6 | 1168.3 | 1168.2/584.6 |

The arrow indicates the cleavage site of trypsin digestion.

Ac-WGCK↓PenGGK↓GGCKPen↓GK↓GGGCGWK↓GGPenGW-$NH_2$

1 2 3 4 5 6

These fragments indicate the formation of 1-4, 2-3, 5-6

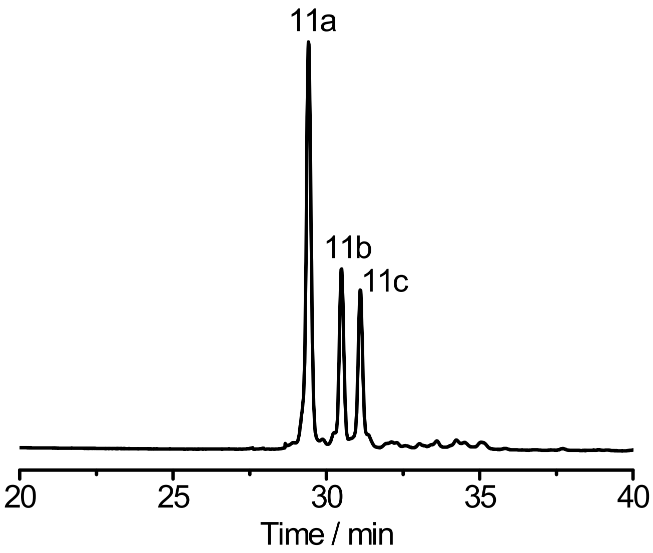


**Figure S33.** Chromatogram of the products formed after the oxidation of peptide **11**.

a)

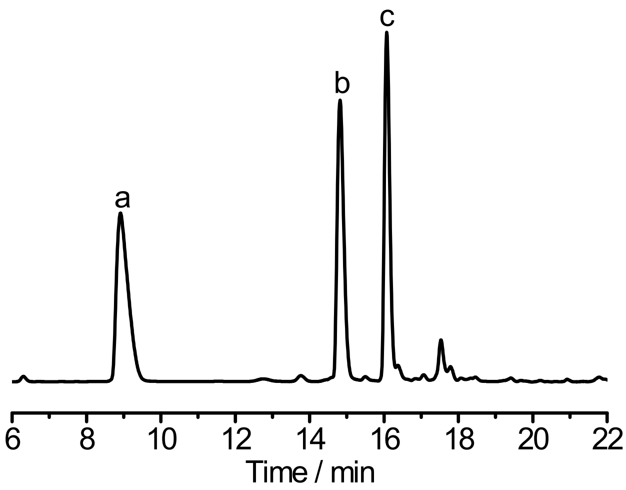

a
b
c
6 8 10 12 14 16 18 20 22
Time / min


b)

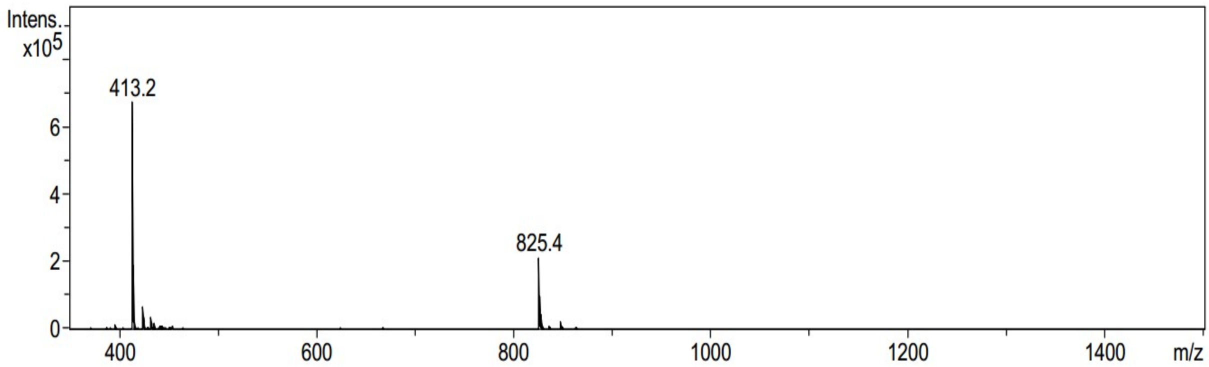

Intens.
x10^5
413.2
825.4
6
4
2
0
400 600 800 1000 1200 1400 m/z


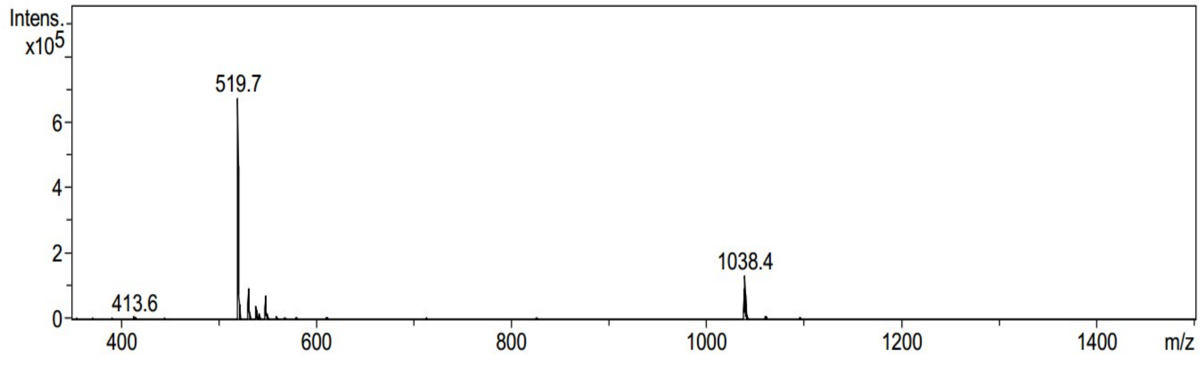

Intens.
x10^5
519.7
413.6
1038.4
6
4
2
0
400 600 800 1000 1200 1400 m/z


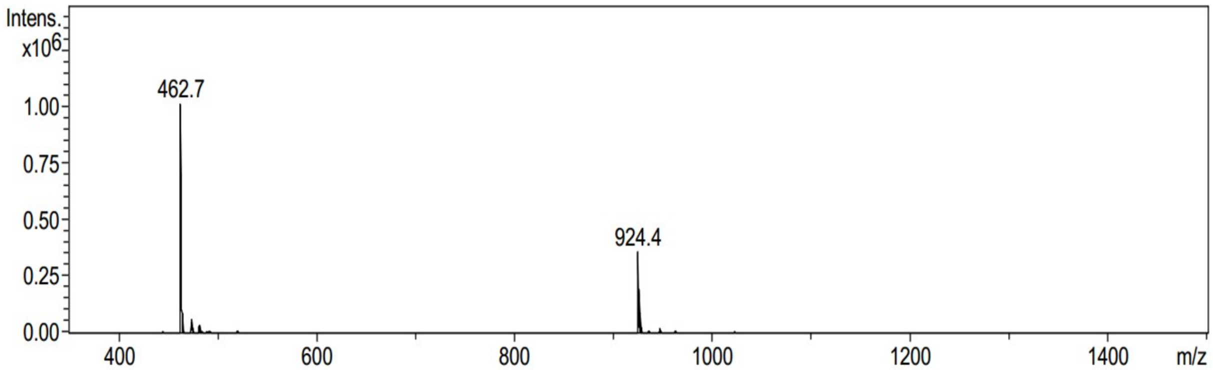

Intens.
x10^6
462.7
924.4
1.00
0.75
0.50
0.25
0.00
400 600 800 1000 1200 1400 m/z

**Figure S34.** Tryptic digestion HPLC/MS analysis of **11a**: a) chromatogram of digested fragments from **11a**; b) mass spectra of fragments a-c labeled in the chromatogram.

**Fragment analysis:**

| Peak NO. | Disulfide pairing | *m/z*(M)$^+$ expected | *m/z*(M)$^+$ or (M+2H)$^{2+}$ found |
|---|---|---|---|
| a | 2-5 | 825.03 | 825.4/413.2 |
| b | 3-6 | 1038.18 | 1038.4/519.7 |
| c | 1-4 | 924.13 | 924.4/462.7 |

The arrow indicates the cleavage site of trypsin digestion.

Ac-WGPenK↓CGK↓GGGC↓GGK↓GGCKPenGWK↓GGPenGW-$NH_2$

1 2 3 4 5 6

These fragments indicate the formation of 1-4, 2-5, 3-6

a)

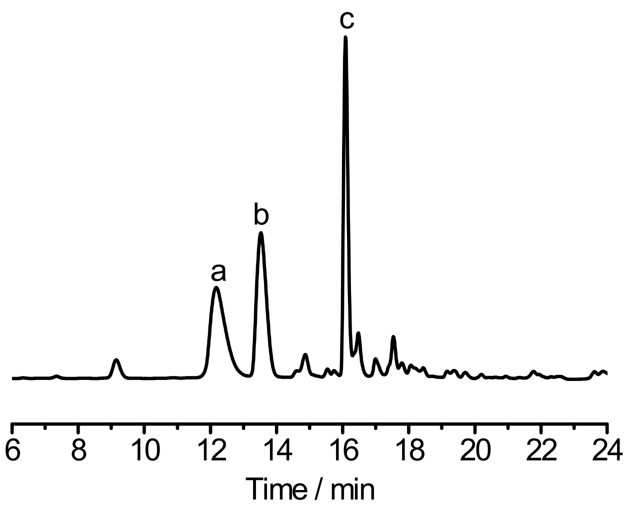

a
b
c
6
8
10
12
14
16
18
20
22
24
Time / min


b)

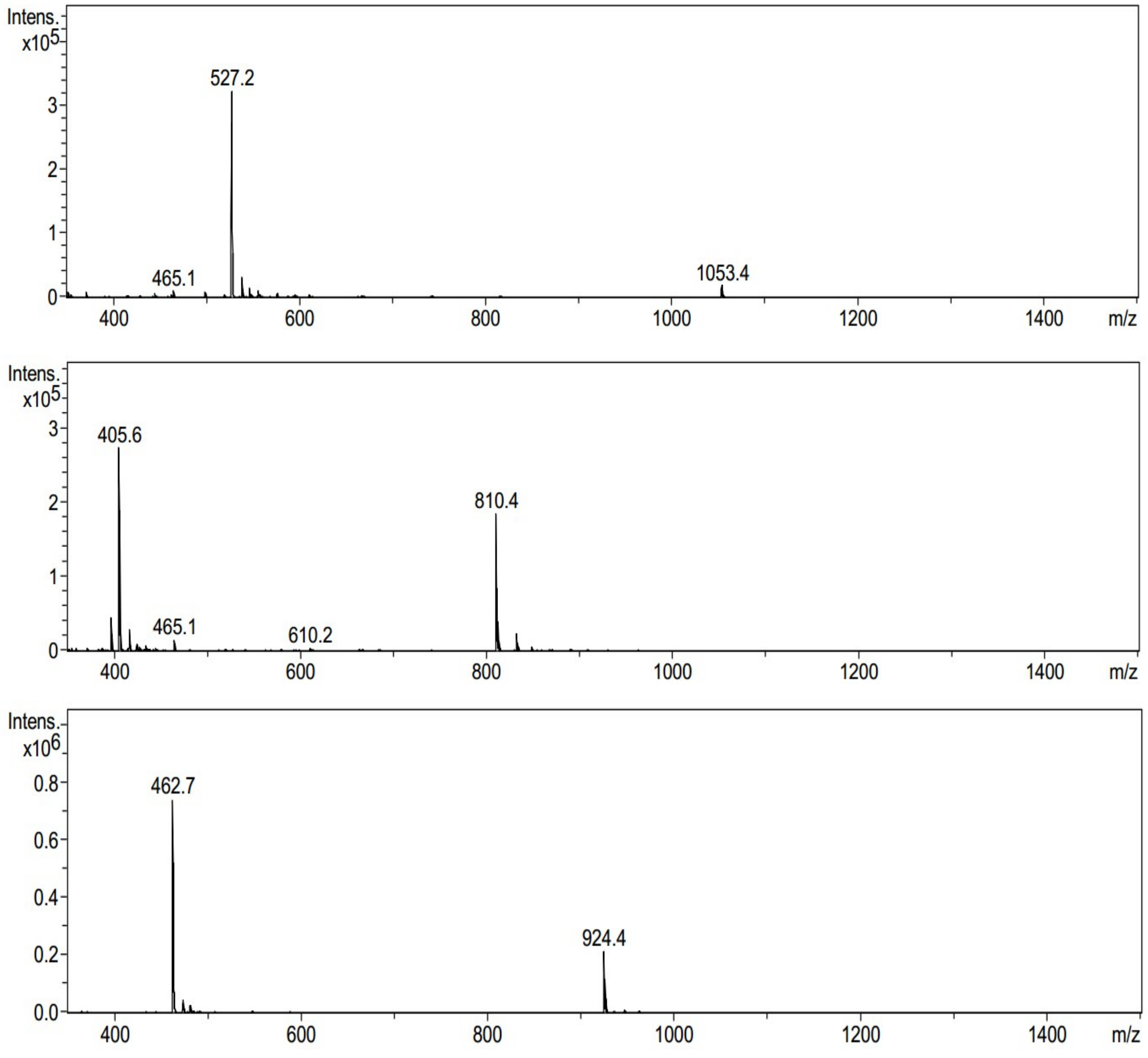

Intens.
x10^5
527.2
465.1
1053.4
m/z
Intens.
x10^5
405.6
810.4
465.1
610.2
m/z
Intens.
x10^6
462.7
924.4
m/z

**Figure S35.** Tryptic digestion HPLC/MS analysis of **11b**: a) chromatogram of digested fragments from **11b**; b) mass spectra of fragments a-c labeled in the chromatogram.

**Fragment analysis:**

| Peak NO. | Disulfide pairing | $m/z$(M)$^+$ expected | $m/z$(M)$^+$ or (M+2H)$^{2+}$ found |
|---|---|---|---|
| a | 3-5 | 1053.23 | 1053.4/527.2 |
| b | 2-6 | 809.98 | 810.4/405.6 |
| c | 1-4 | 924.13 | 924.4/462.7 |

The arrow indicates the cleavage site of trypsin digestion.

Ac-WGPenK↓CGK↓GGGC↓GGK↓GGCKPenGWK↓GGPenGW-$NH_2$
1 2 3 4 5 6

These fragments indicate the formation of 1-4, 2-6, 3-5

a)

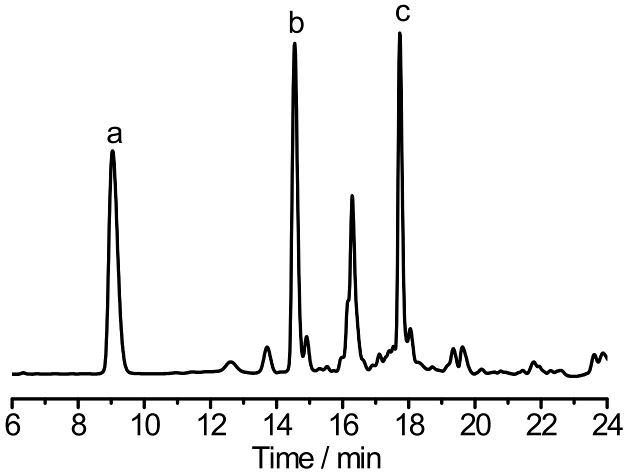
a
b
c
6
8
10
12
14
16
18
20
22
24
Time / min

b)

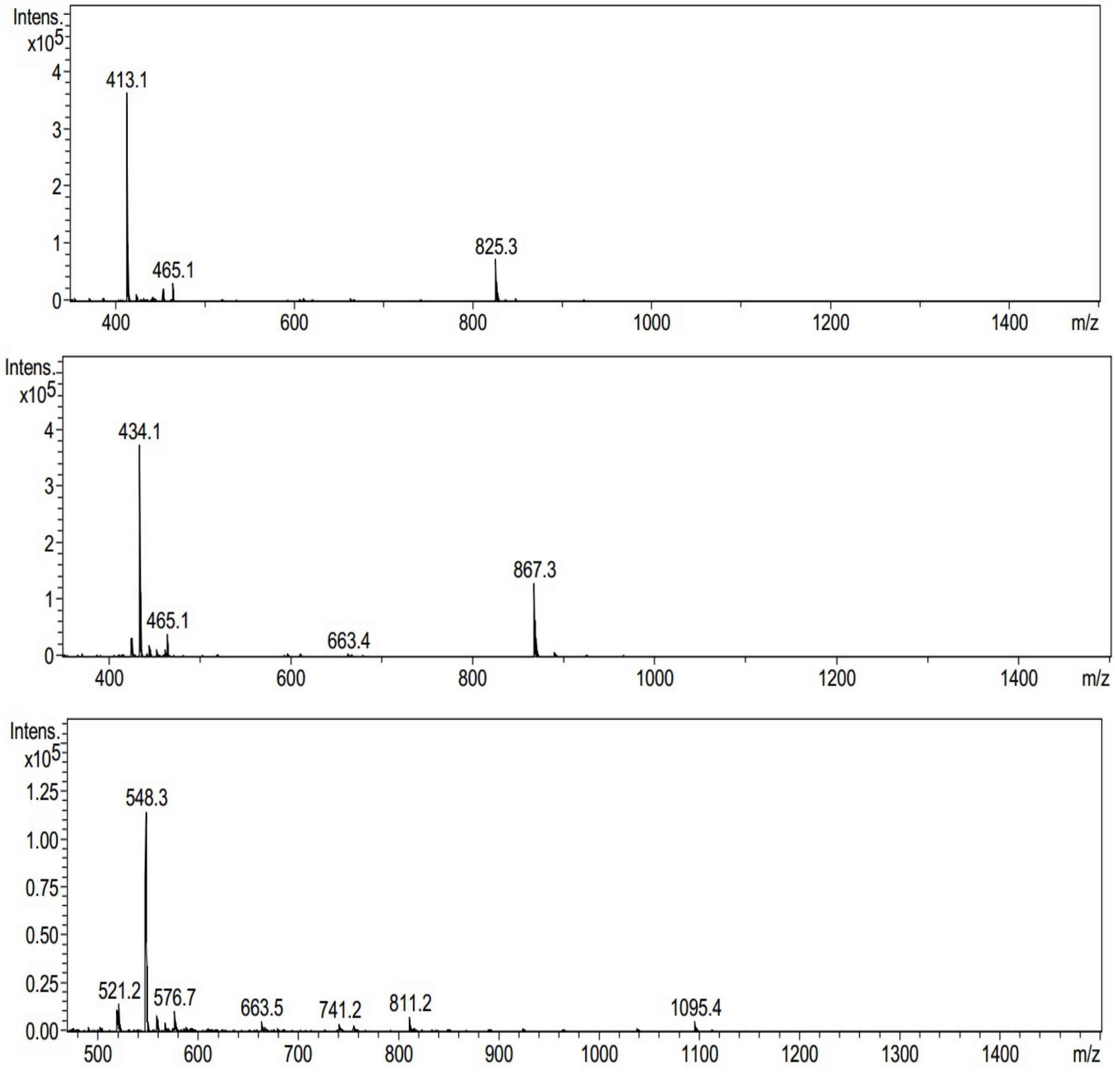
Intens.
x10^5
413.1
465.1
825.3
m/z
434.1
465.1
663.4
867.3
548.3
521.2
576.7
663.5
741.2
811.2
1095.4

**Figure S36.** Tryptic digestion HPLC/MS analysis of **11c**: a) chromatogram of digested fragments from **11c**; b) mass spectra of fragments a-c labeled in the chromatogram.

**Fragment analysis:**

| **Peak NO.** | **Disulfide pairing** | ***m/z*(M)$^+$ expected** | ***m/z*(M)$^+$ or (M+2H)$^{2+}$ found** |
|---|---|---|---|
| a | 2-5 | 825.03 | 825.3/413.1 |
| b | 4-6 | 867.03 | 867.3/434.1 |
| c | 1-3 | 1095.28 | 1095.4/548.3 |

The arrow indicates the cleavage site of trypsin digestion.

Ac-WGPenK↓CGK↓GGGC↓GGK↓GGCKPenGWK↓GGPenGW-$NH_2$

1 2 3 4 5 6

These fragments indicate the formation of 1-3, 2-5, 4-6

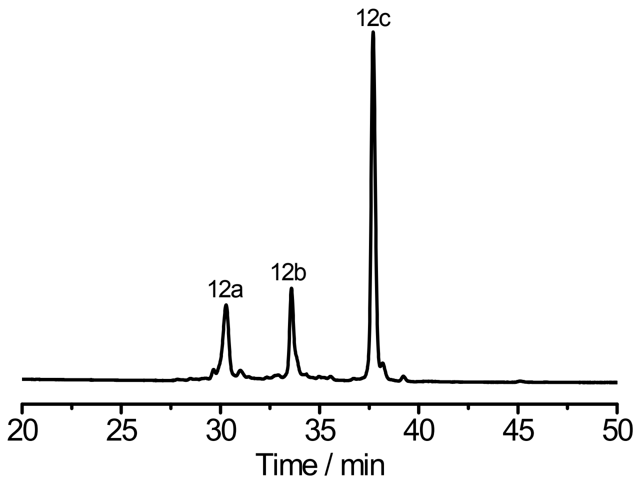


**Figure S37.** Chromatogram of the products formed after the oxidation of peptide **12**.

a)

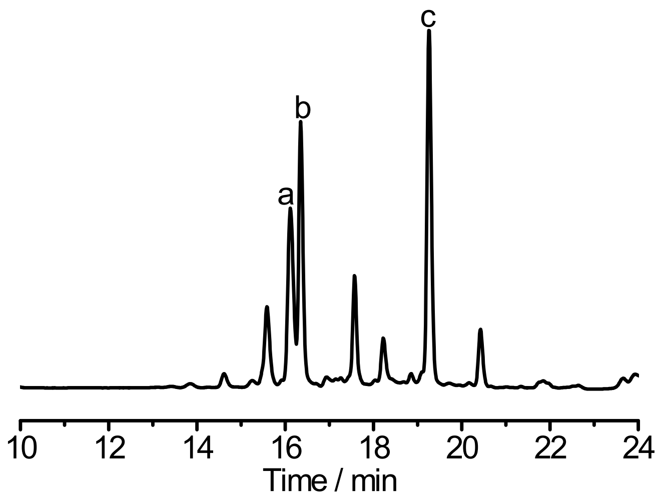


b)

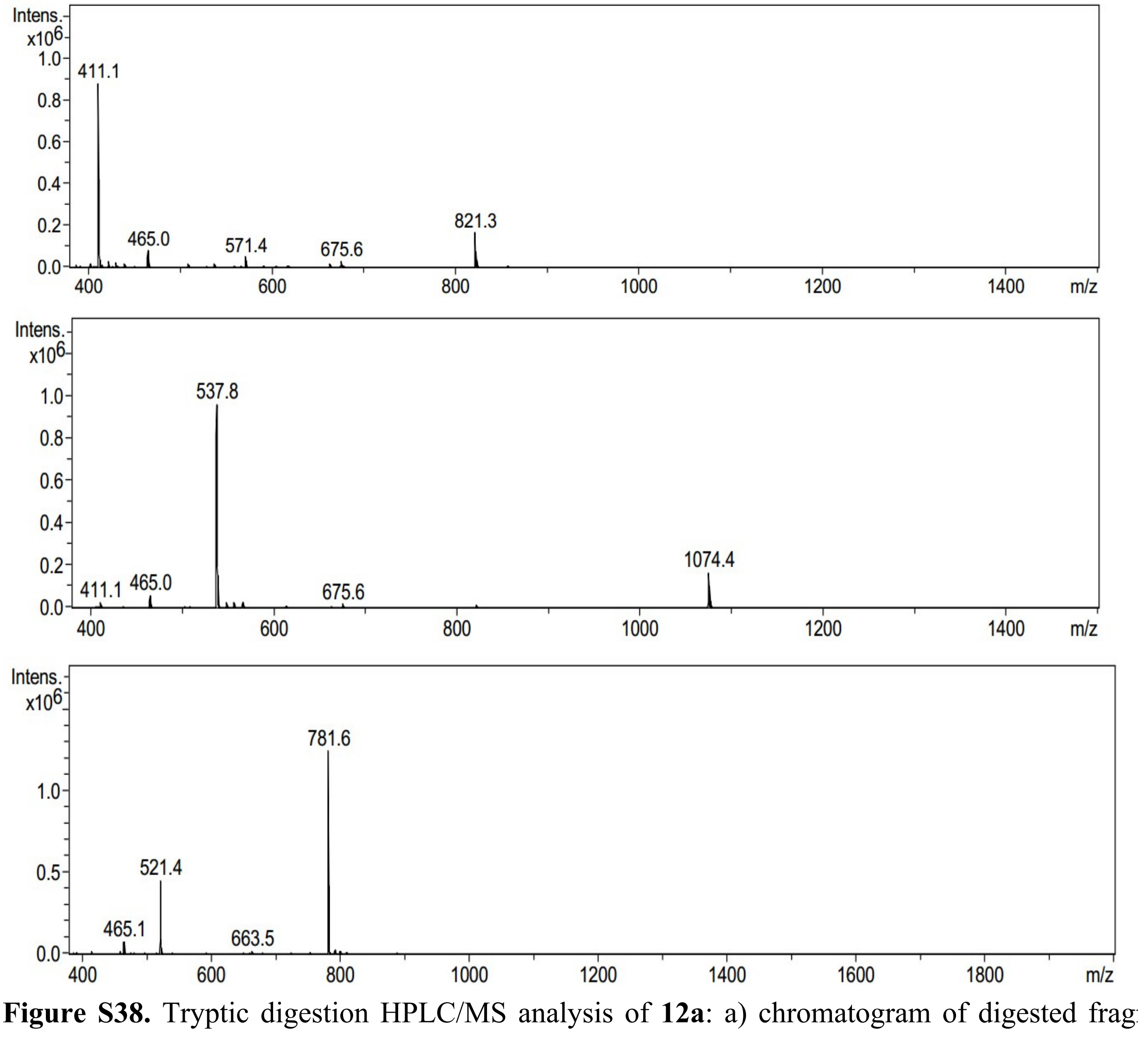


**Figure S38.** Tryptic digestion HPLC/MS analysis of **12a**: a) chromatogram of digested fragments from **12a**; b) mass spectra of fragments a-c labeled in the chromatogram.

**Fragment analysis:**

| Peak NO. | Disulfide pairing | *m/z*$(M)^+$ expected | *m/z*$(M)^+$ or $(M+2H)^{2+}$ or $(M+3H)^{3+}$found |
|---|---|---|---|
| a | 3-6 | 821.01 | 821.3/411.1 |
| b | 3-6 | 1074.31 | 1074.4/537.8 |
| c | 1-4 | 1561.82 | 781.6/521.4 |

The arrow indicates the cleavage site of trypsin digestion.

Ac-WGCK↓PenGK↓GCPR↓PR↓GDNPPLTPenGK↓GCK↓PenGW-$NH_2$

1 2 3 4 5 6

These fragments indicate the formation of 1-4, 2-5, 3-6

Fragment a:

**GCPR PenGW**

Fragment b:

**GCPRPR PenGW**

a)

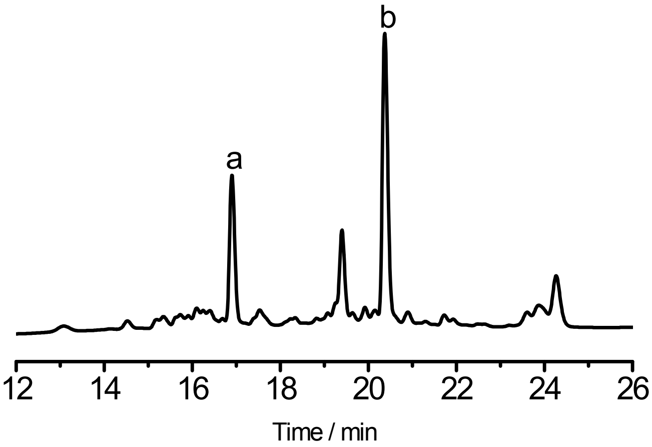


b)

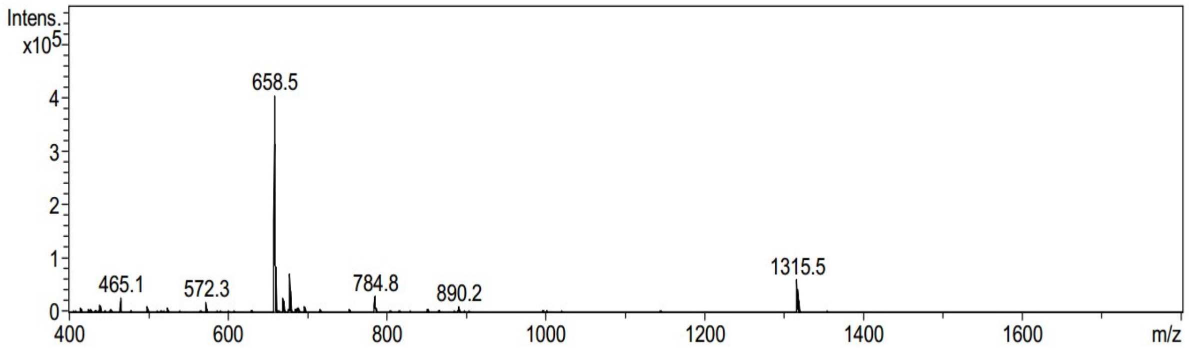


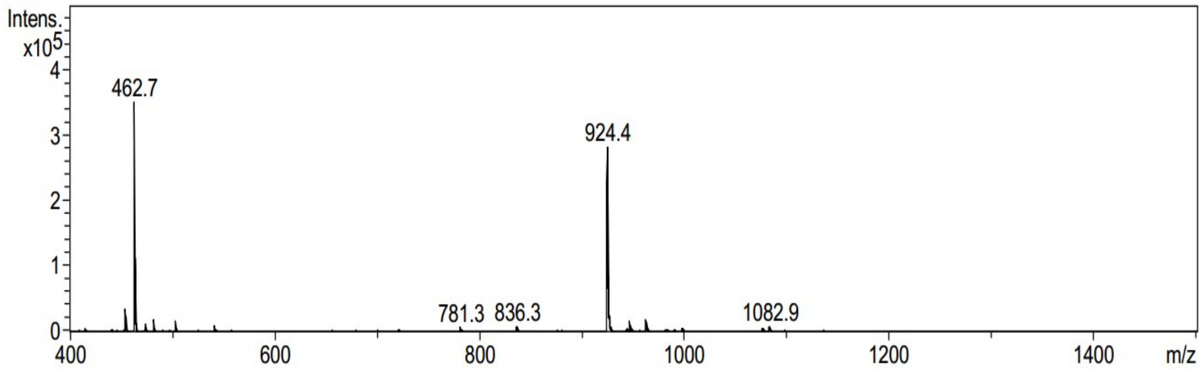


**Figure S39.** Tryptic digestion HPLC/MS analysis of **12b**: a) chromatogram of digested fragments from **12b**; b) mass spectra of fragments a and b labeled in the chromatogram.

**Fragment analysis:**

| Peak NO. | Disulfide pairing | *m/z*(M)$^+$ expected | *m/z*(M)$^+$ or (M+2H)$^{2+}$ found |
|---|---|---|---|
| a | 4-5 | 1315.57 | 1315.5/658.5 |
| b | 1-6 | 924.13 | 924.4/462.7 |

The arrow indicates the cleavage site of trypsin digestion.

Ac-WGCK↓PenGK↓GCPR↓PR↓GDNPPLTPenGK↓GCK↓PenGW-$NH_2$

1 2 3 4 5 6

These fragments indicate the formation of 1-6, 2-3, 4-5

a)

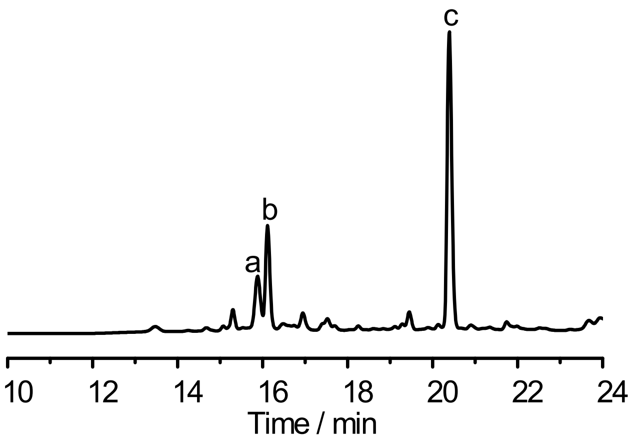


b)

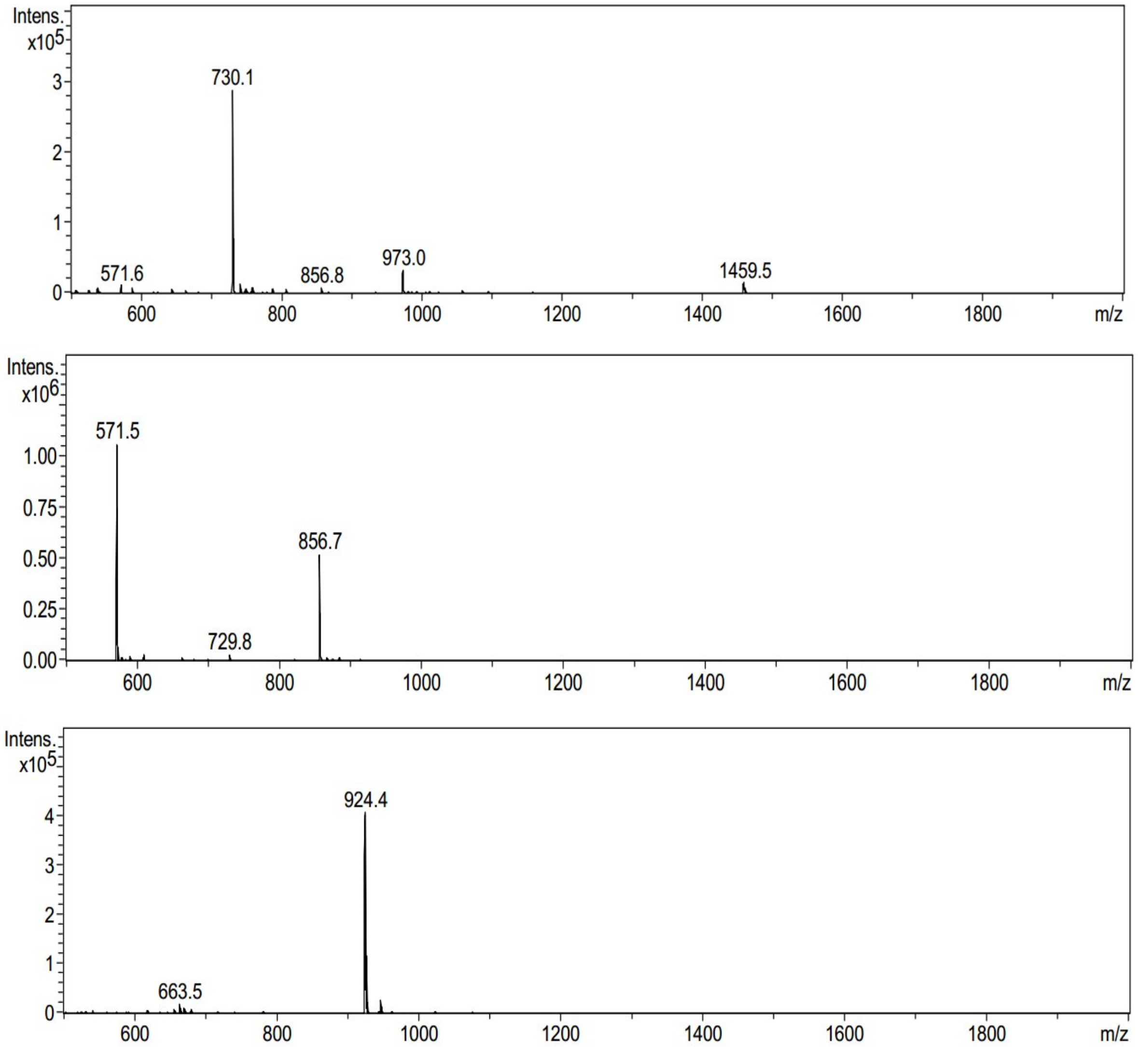


**Figure S40.** Tryptic digestion HPLC/MS analysis of **12c**: a) chromatogram of digested fragments from **12c**; b) mass spectra of fragments a-c labeled in the chromatogram.

**Fragment analysis:**

| Peak NO. | Disulfide pairing | *m/z*(M)$^+$ expected | *m/z*(M)$^+$ or (M+2H)$^{2+}$ or (M+3H)$^{3+}$ found |
|---|---|---|---|
| a | 3-4 | 1458.7 | 1459.5/730.1 |
| b | 3-4 | 1712.0 | 856.7/571.5 |
| c | 1-6 | 924.13 | 924.4/462.7 |

The arrow indicates the cleavage site of trypsin digestion.

Ac-WGCK↓PenGK↓GCPR↓PR↓GDNPPLTPenGK↓GCK↓PenGW-$NH_2$

1 2 3 4 5 6

These fragments indicate the formation of 1-6, 2-5, 3-4

Fragment a:

**GCPR GDNPPLTPenGK**

Fragment b:

**GCPRPR GDNPPLTPenGK**

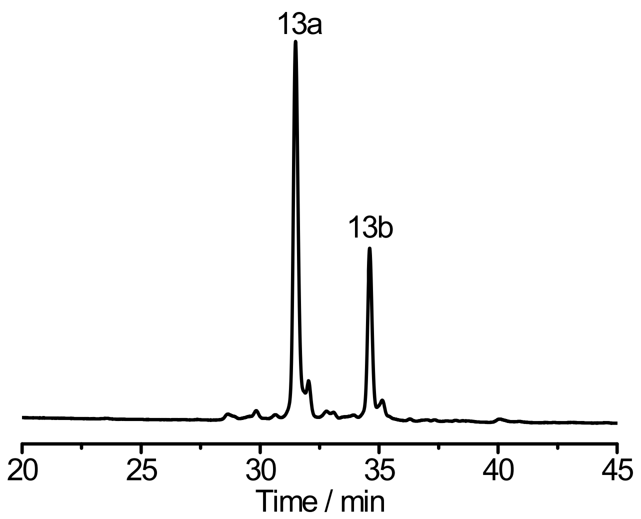


**Figure S41.** Chromatogram of the products formed after the oxidation of peptide **13**.

a)

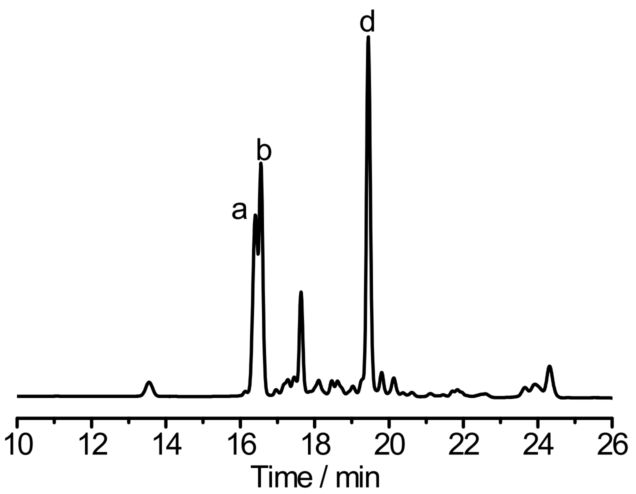

a
b
d
10 12 14 16 18 20 22 24 26
Time / min


b)

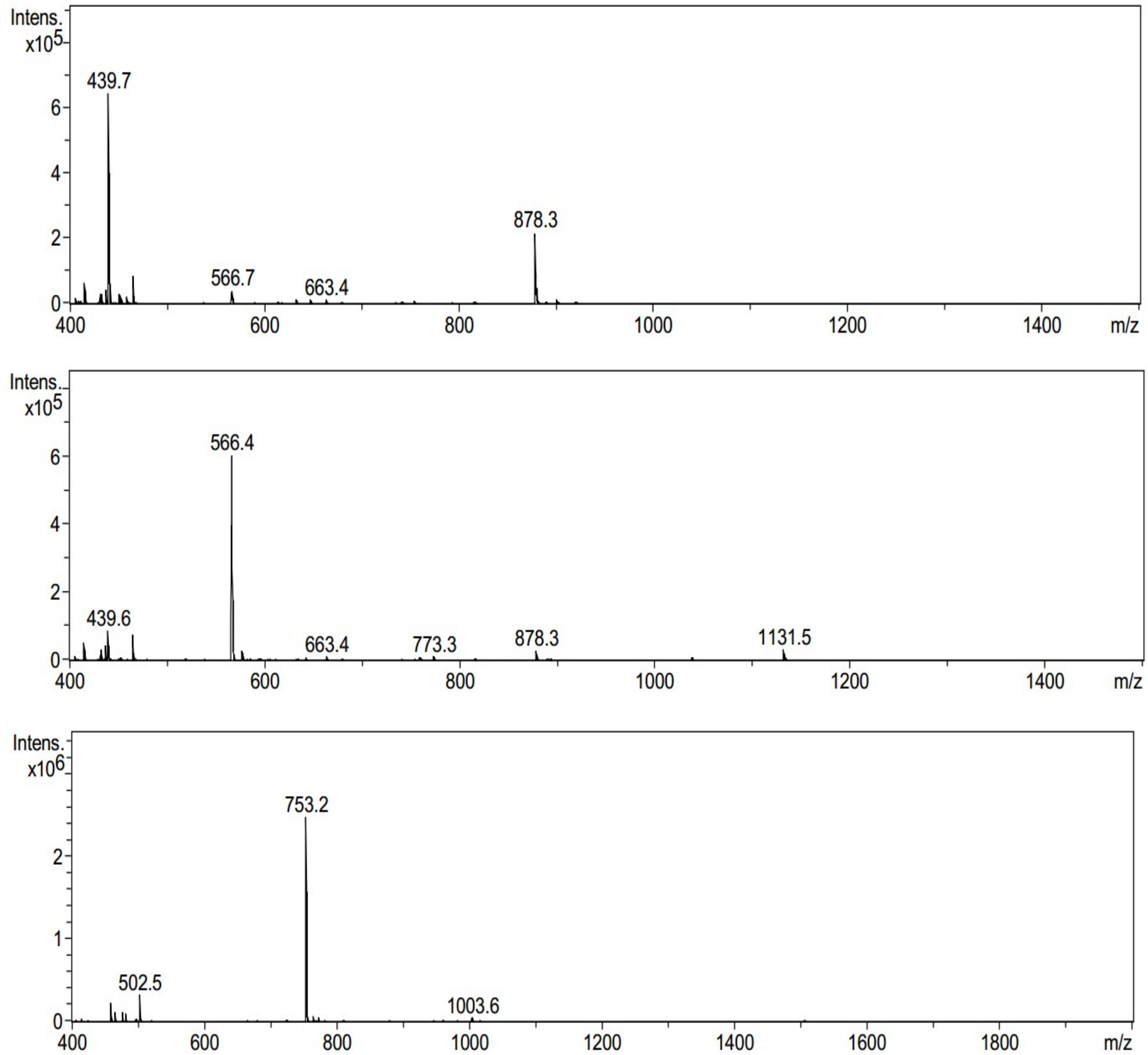

Intens. x10^5
439.7
878.3
566.7
663.4
m/z
Intens. x10^5
566.4
439.6
663.4
773.3
878.3
1131.5
m/z
Intens. x10^6
753.2
502.5
1003.6
m/z

**Figure S42.** Tryptic digestion HPLC/MS analysis of **13a**: a) chromatogram of digested fragments from **13a**; b) mass spectra of fragments a-c labeled in the chromatogram.

**Fragment analysis:**

| **Peak NO.** | **Disulfide pairing** | ***m/z*(M)$^+$ expected** | ***m/z*(M)$^+$ or (M+2H)$^{2+}$ found** |
|---|---|---|---|
| a | 3-6 | 878.06 | 878.3/439.7 |
| b | 3-6 | 1131.36 | 1131.5/566.4 |
| c | 1-4 | 1054.77 | 753.2 |

The arrow indicates the cleavage site of trypsin digestion.

Ac-WGPenK↓CGK↓GCPR↓PR↓GDNPPLTCK↓PenGK↓GPenGW-$NH_2$

1 2 3 4 5 6

These fragments indicate the formation of 1-4, 2-5, 3-6

Fragment a:

**GCPR GPenGW**

Fragment b:

**GCPRPR GPenGW**

a)

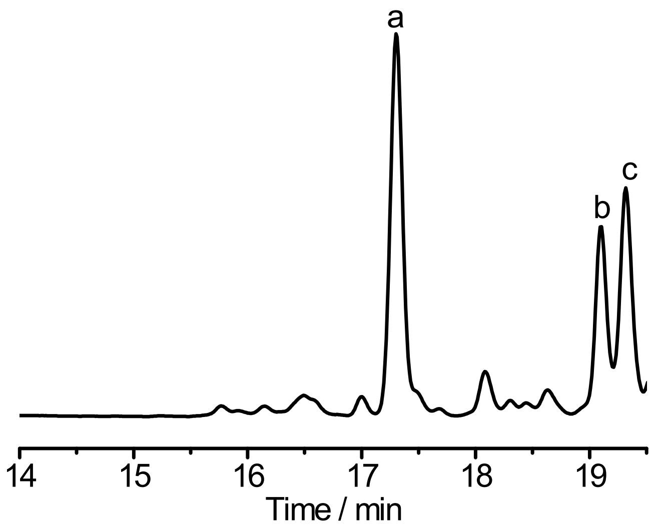

a
b
c
14
15
16
17
18
19
Time / min


b)

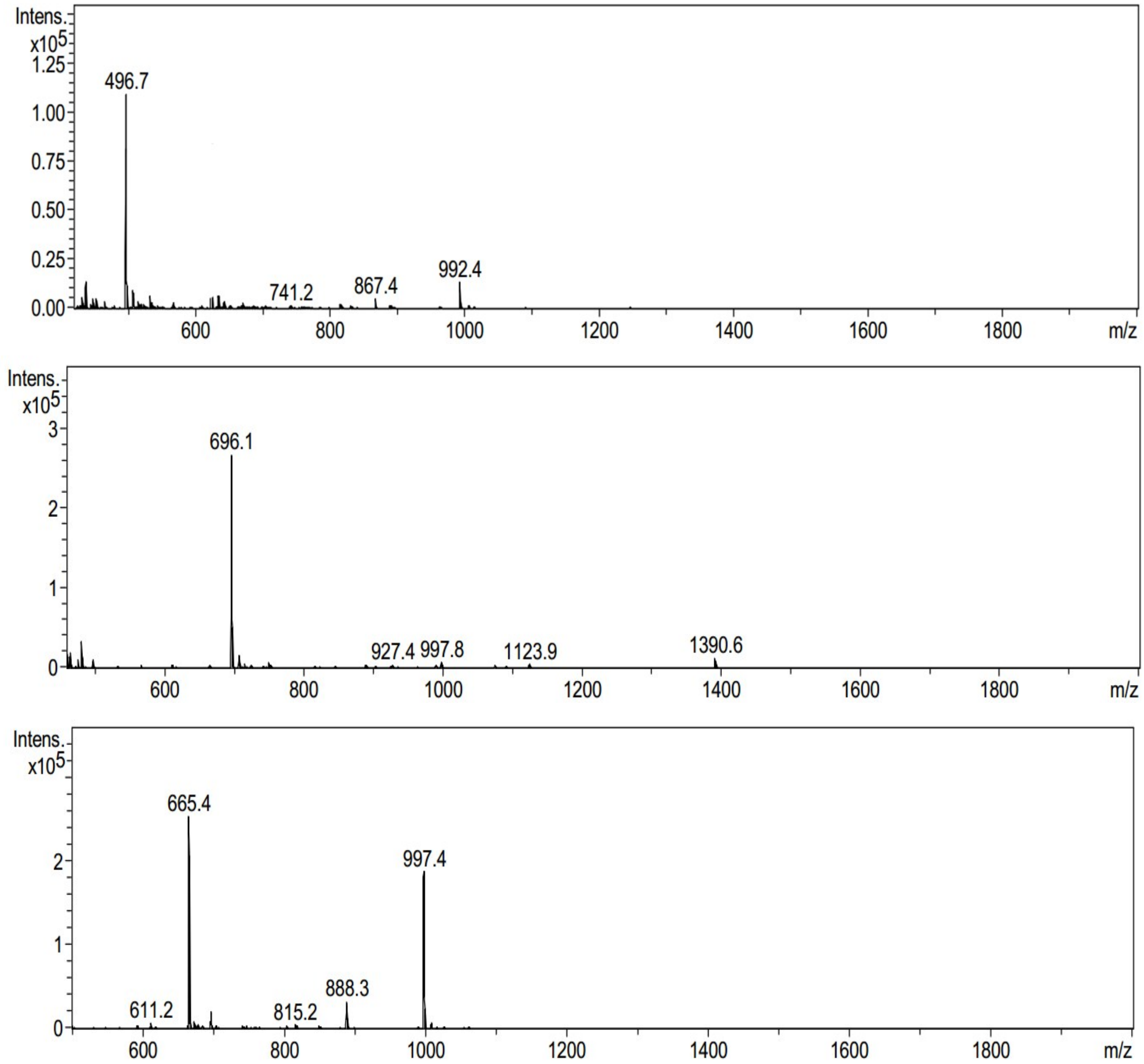

Intens.
x10^5
1.25
1.00
0.75
0.50
0.25
0.00
496.7
741.2
867.4
992.4
600
800
1000
1200
1400
1600
1800
m/z
Intens.
x10^5
3
2
1
0
696.1
927.4
997.8
1123.9
1390.6
Intens.
x10^5
2
1
0
665.4
997.4
611.2
815.2
888.3

**Figure S43.** Tryptic digestion HPLC/MS analysis of **13b**: a) chromatogram of digested fragments from **13b**; b) mass spectra of fragments a-c labeled in the chromatogram.

**Fragment analysis:**

| **Peak NO.** | **Disulfide pairing** | ***m/z*(M)⁺ expected** | ***m/z*(M)⁺ or (M+2H)²⁺ or (M+3H)³⁺ found** |
|---|---|---|---|
| a | 1-3 | 992.21 | 992.4/496.7 |
| b | 4-6 | 1390.62 | 1390.6/696.1 |
| c | 2-5 and 4-6 | 1995.45 | 997.4/665.4 |

The arrow indicates the cleavage site of trypsin digestion.

Ac-WGPenK↓CGK↓GCPR↓PR↓GDNPPLTCK↓PenGK↓GPenGW-$NH_2$
1 2 3 4 5 6

These fragments indicate the formation of 1-3, 2-5, 4-6

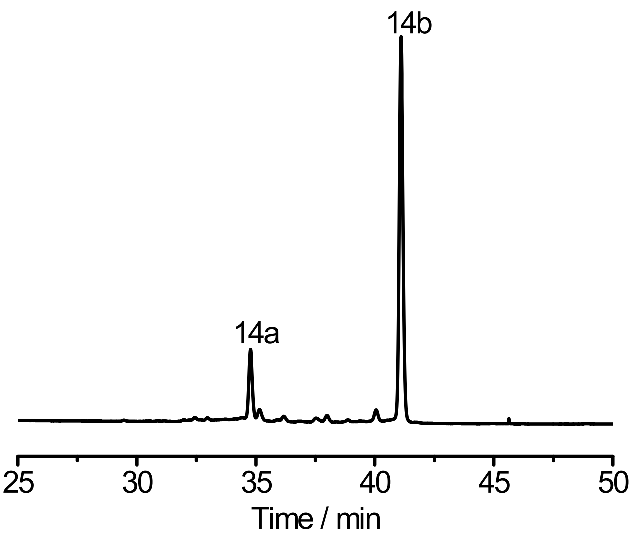


**Figure S44.** Chromatogram of the products formed after the oxidation of peptide **14**.

a)

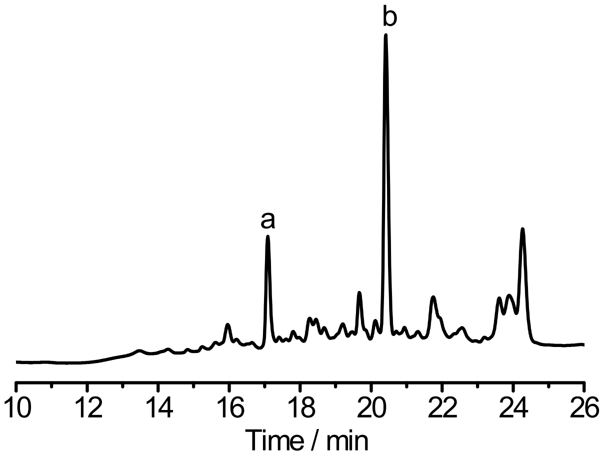


b)

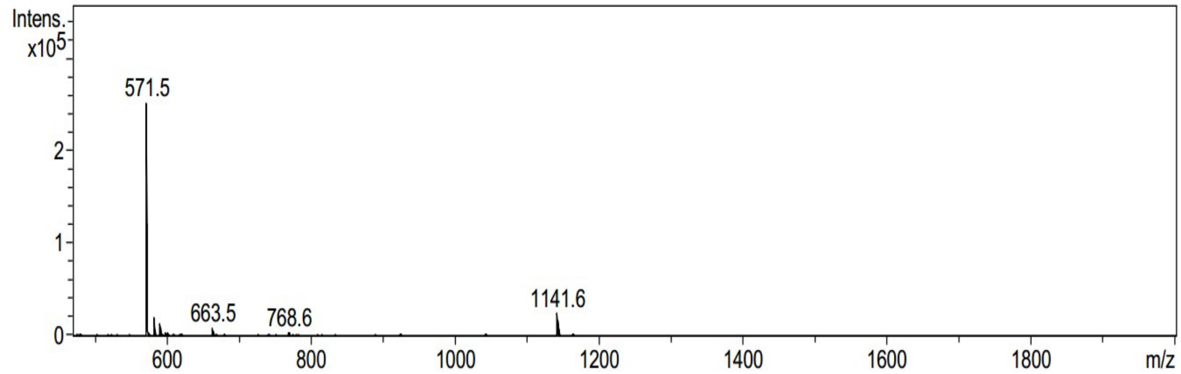


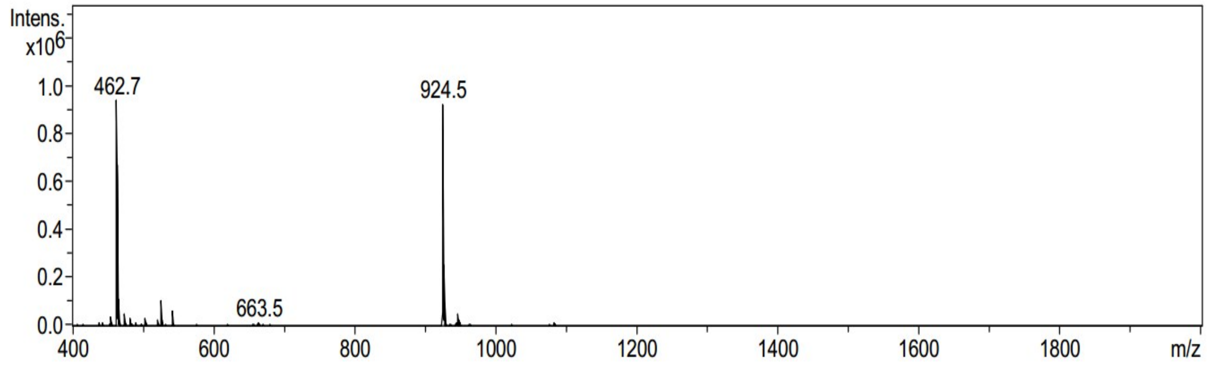


**Figure S45.** Tryptic digestion HPLC/MS analysis of **14a**: a) chromatogram of digested fragments from **14a**; b) mass spectra of fragments a and b labeled in the chromatogram.

**Fragment analysis:**

| Peak NO. | Disulfide pairing | *m/z*(M)$^+$ expected | *m/z*(M)$^+$ or (M+2H)$^{2+}$ or (M+3H)$^{3+}$ found |
|---|---|---|---|
| a | 2-3 | 1141.43 | 1141.6/571.5 |
| b | 1-6 | 924.13 | 924.5/462.7 |

The arrow indicates the cleavage site of trypsin digestion.

Ac-WGCK↓PenAEK↓VGCLFK↓K↓SPenQNKPTCK↓PenGW-$NH_2$

1 2 3 4 5 6

These fragments indicate the formation of 1-6, 2-3, 4-5

a)

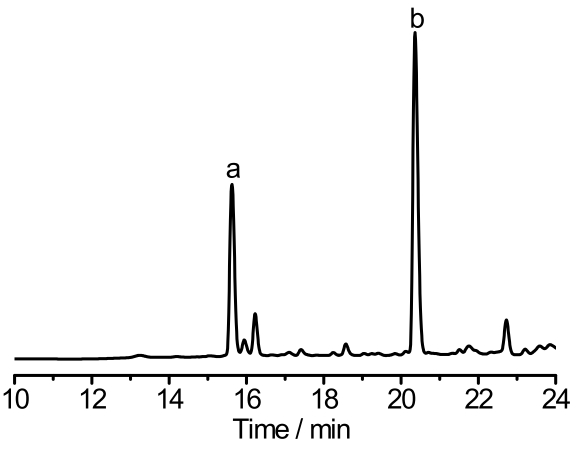


b)

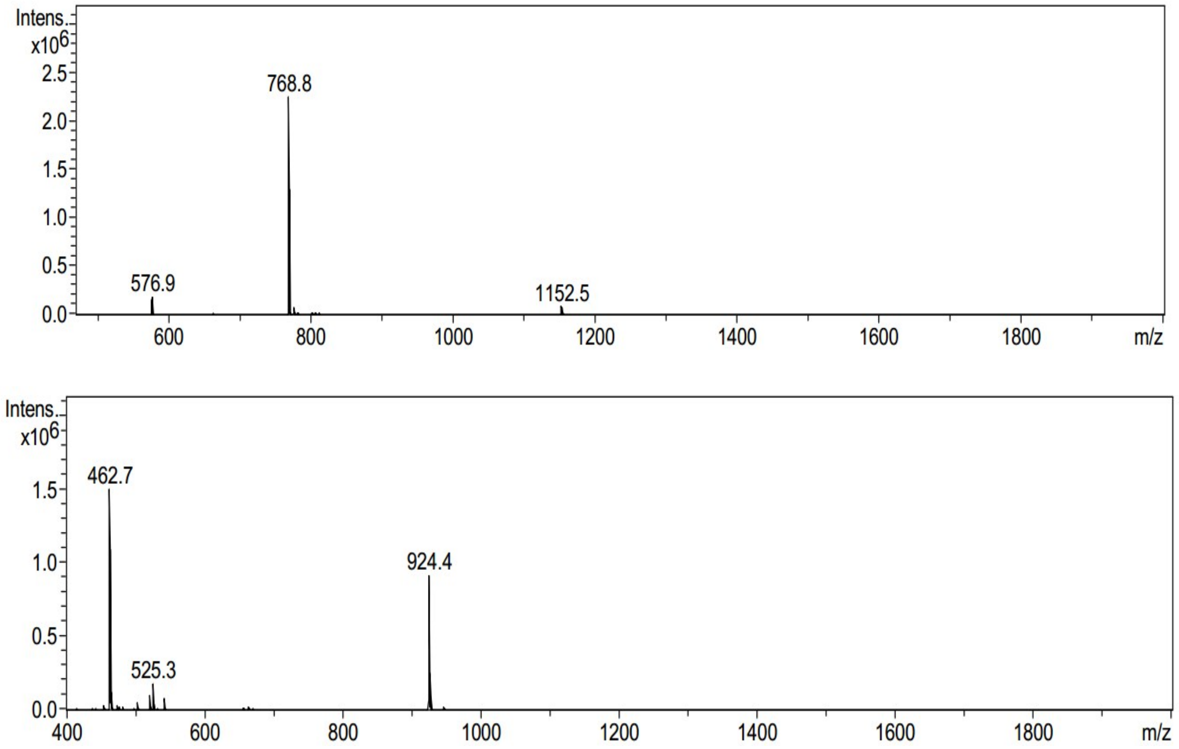


**Figure S46.** Tryptic digestion HPLC/MS analysis of **14b**: a) chromatogram of digested fragments from **14b**; b) mass spectra of fragments a and b labeled in the chromatogram.

**Fragment analysis:**

| Peak NO. | Disulfide pairing | $m/z$(M)$^+$ expected | $m/z$(M)$^+$ or (M+2H)$^{2+}$ or (M+3H)$^{3+}$ found |
|---|---|---|---|
| a | 2-5 and 3-4 | 2303.83 | 1152.5/768.8 |
| b | 1-6 | 924.13 | 924.5/462.7 |

The arrow indicates the cleavage site of trypsin digestion.

Ac-WGCK↓PenAEK↓VGCLFK↓K↓SPenQNKPTCK↓PenGW-$NH_2$

1 2 3 4 5 6

These fragments indicate the formation of 1-6, 2-5, 3-4

Fragemnts a：

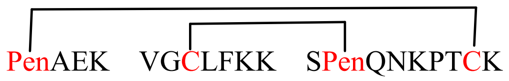

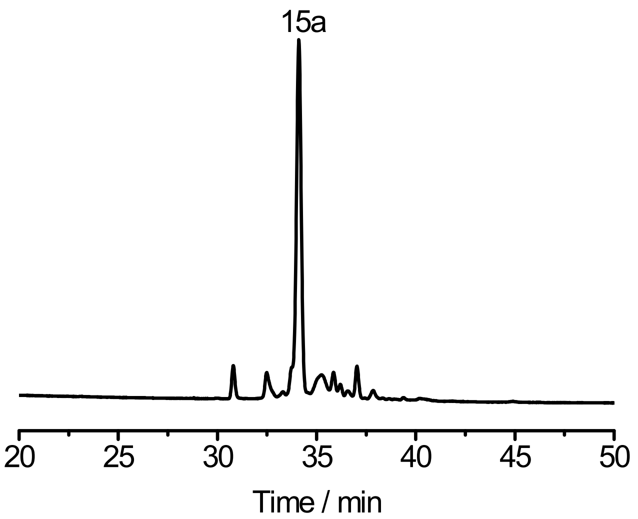


**Figure S47.** Chromatogram of the products formed after the oxidation of peptide **15**.

a)

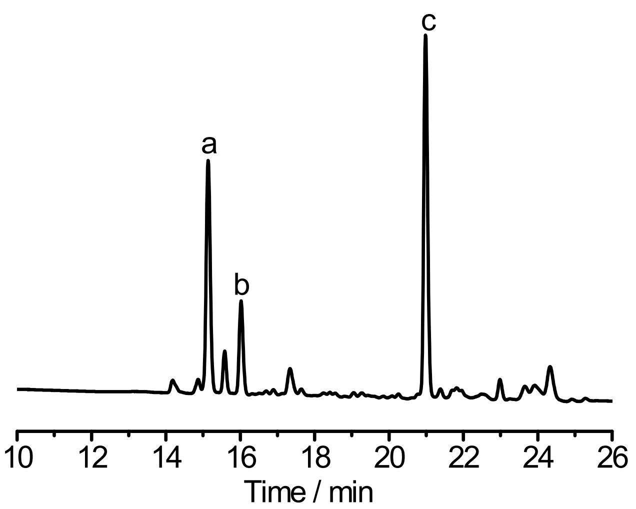
a
b
c
10 12 14 16 18 20 22 24 26
Time / min

b)

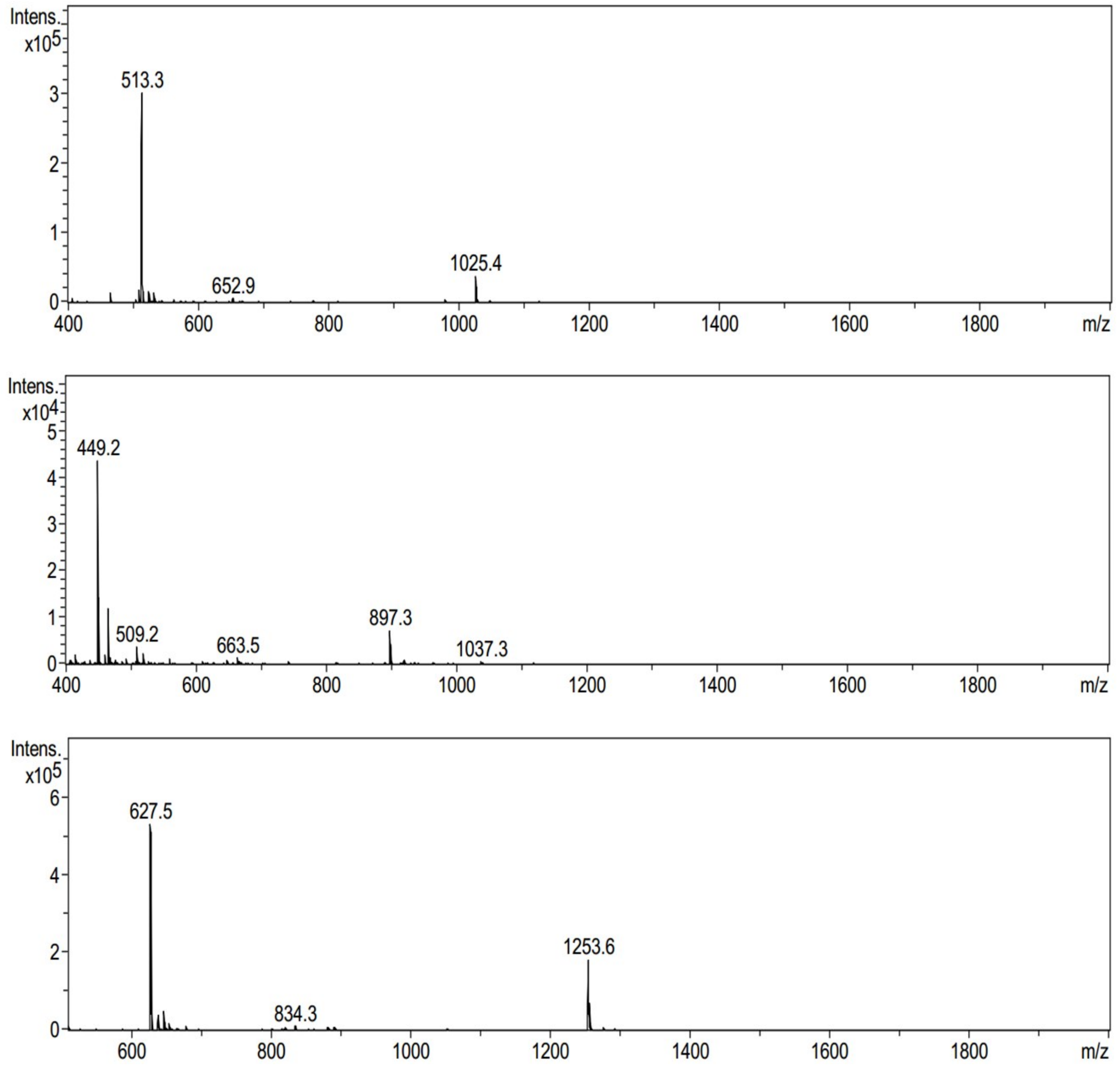
Intens.
x10^5
513.3
652.9
1025.4
400 600 800 1000 1200 1400 1600 1800 m/z
Intens.
x10^4
449.2
509.2
663.5
897.3
1037.3
400 600 800 1000 1200 1400 1600 1800 m/z
Intens.
x10^5
627.5
834.3
1253.6
600 800 1000 1200 1400 1600 1800 m/z

**Figure S48.** Tryptic digestion HPLC/MS analysis of **15a**: a) chromatogram of digested fragments from **15a**; b) mass spectra of fragments a-c labeled in the chromatogram.

**Fragment analysis:**

| **Peak NO.** | **Disulfide pairing** | ***m/z*(M)$^+$ expected** | ***m/z*(M)$^+$ or (M+2H)$^{2+}$ found** |
|---|---|---|---|
| a | 1-4 | 897.1 | 1152.5/768.8 |
| b | 1-4 | 1025.28 | 1025.4/513.3 |
| c | 3-6 | 1253.56 | 1253.6/627.5 |

The arrow indicates the cleavage site of trypsin digestion.

Ac-WGPenK↓CAEK↓VGCLFK↓K↓SCK↓PenQNK↓TPPenGW-$NH_2$

1 2 3 4 5 6

These fragments indicate the formation of 1-4, 2-5, 3-6

Fragemnts a:

Ac-WGPenK KSCK

Fragemnts b:

Ac-WGPenK SCK

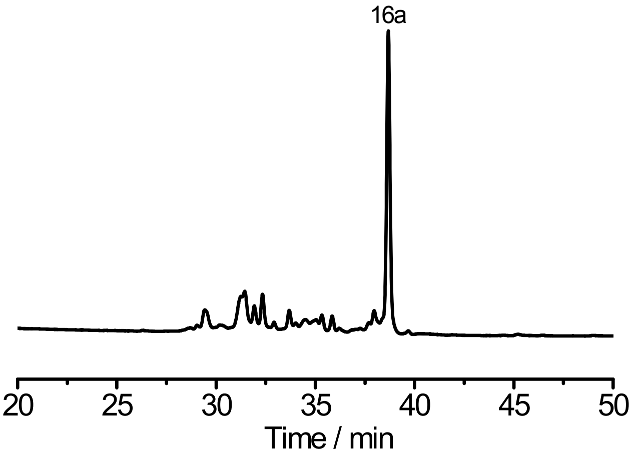


**Figure S49.** Chromatogram of the products formed after the oxidation of peptide **16**.

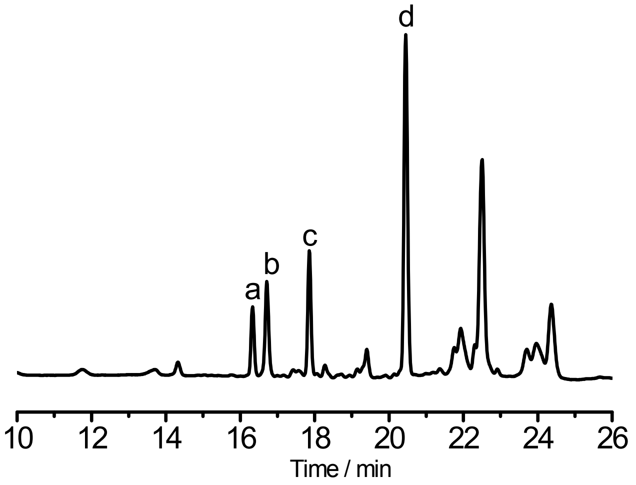

d
c
b
a
10
12
14
16
18
20
22
24
26
Time / min


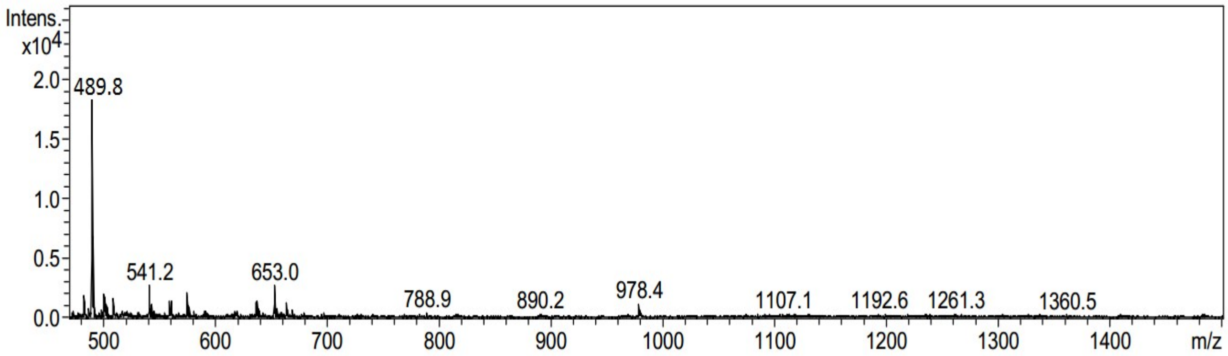

Intens.
x10^4
2.0
1.5
1.0
0.5
0.0
489.8
541.2
653.0
788.9
890.2
978.4
1107.1
1192.6
1261.3
1360.5
500
600
700
800
900
1000
1100
1200
1300
1400
m/z


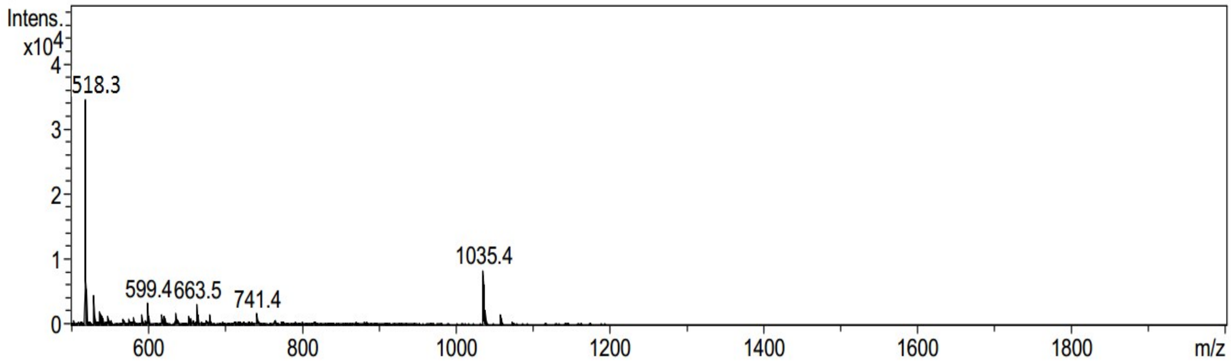

Intens.
x10^4
4
3
2
1
0
518.3
599.4
663.5
741.4
1035.4
600
800
1000
1200
1400
1600
1800
m/z


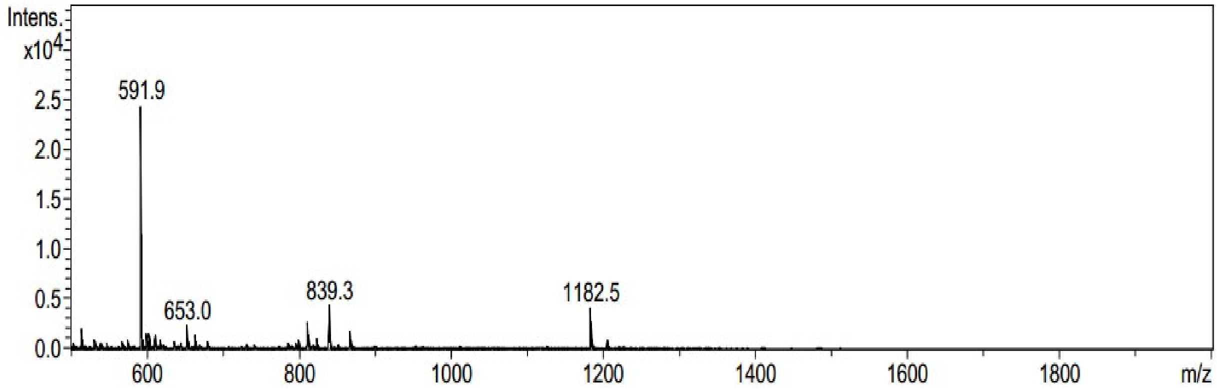

Intens.
x10^4
2.5
2.0
1.5
1.0
0.5
0.0
591.9
653.0
839.3
1182.5
600
800
1000
1200
1400
1600
1800
m/z

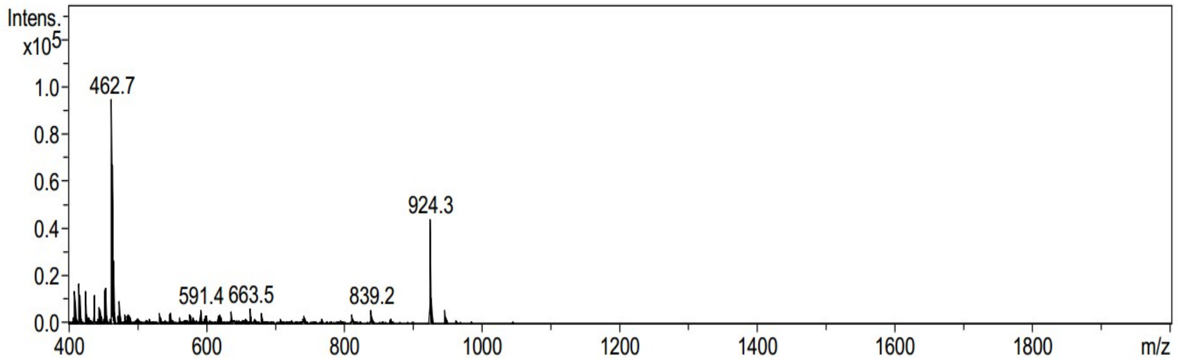


**Figure S50.** Tryptic digestion HPLC/MS analysis of **16a**: a) chromatogram of digested fragments from **16a**; b) mass spectra of fragments a-d labeled in the chromatogram.

**Fragment analysis:**

| Peak NO. | Disulfide pairing | *m/z*$(M)^+$ expected | *m/z*$(M)^+$ or $(M+2H)^{2+}$ found |
|---|---|---|---|
| a* | 5-6 | 978.191 | 978.4/489.8 |
| b* | 3-4 | 1253.56 | 1035.4/518.3 |
| c | 3-6 | 1182.4 | 1182.5/591.9 |
| d | 1-8 | 924.13 | 924.3/462.7 |

The arrow indicates the cleavage site of trypsin digestion.

Ac-WGCK↓PenGK↓GGPenGWK↓GCGGK↓GPenGGK↓GCGWK↓GCKPenGW-$NH_2$
1 2 3 4 5 6 7 8

a*: the cleavage site within the GPenGGKGCGWK fragment was not cleaved.

b*: the cleavage site within the GGPenGWKGCGGK fragment was not cleaved.

Fragments a*, b* and d indicate the formation of 1-8, 2-7, 3-4, 5-6.

Fragments c and d indicate the formation of 1-8, 2-7, 3-6, 4-5 or 1-8, 2-4, 3-6, 5-7. We speculate that it is most likely a connectivity of 1-8, 2-7, 3-6, 4-5, because the folding with connectivity of 1-8, 2-7, 3-4, 5-6 and 1-8, 2-7, 3-6, 4-5 are equally probable, and the formation of 1-8, 2-7, 3-4, 5-6 has been identified.

# Characterization of peptides

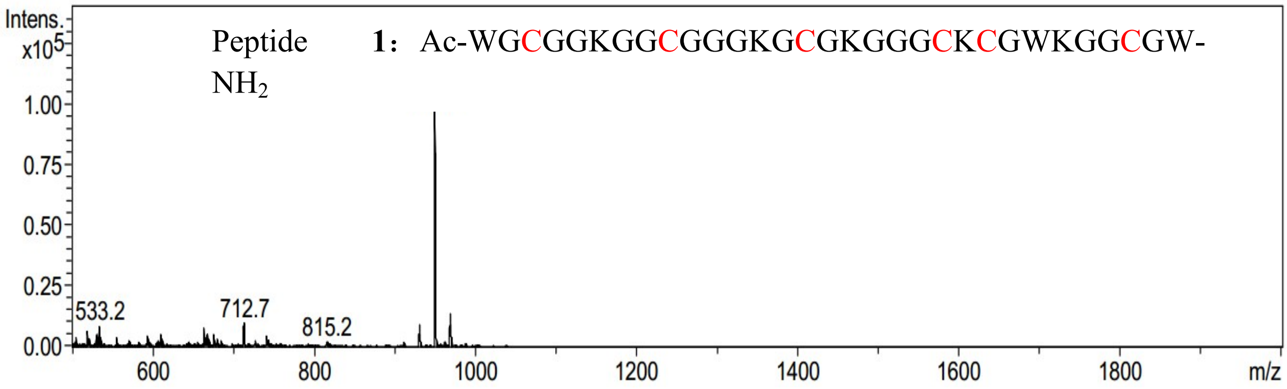


Mass spectrum of peptide **1**. Calcd (m/z): 2847.36; found 949.9 $(M+3H)^{3+}$, 712.7 $(M+4H)^{4+}$.

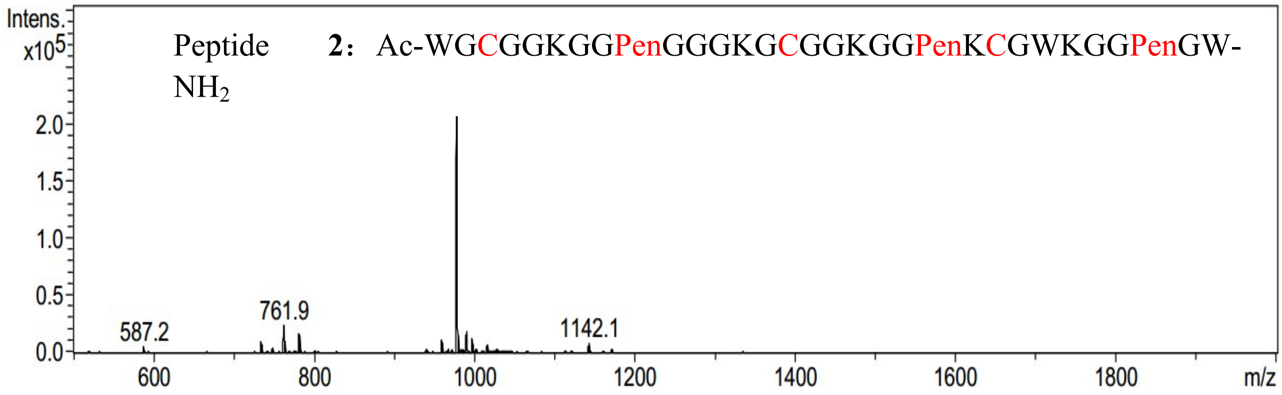


Mass spectrum of peptide **2**. Calcd (m/z): 2931.51; found 977.8 $(M+3H)^{3+}$, 733.7 $(M+4H)^{4+}$. 587.2 $(M+5H)^{5+}$.

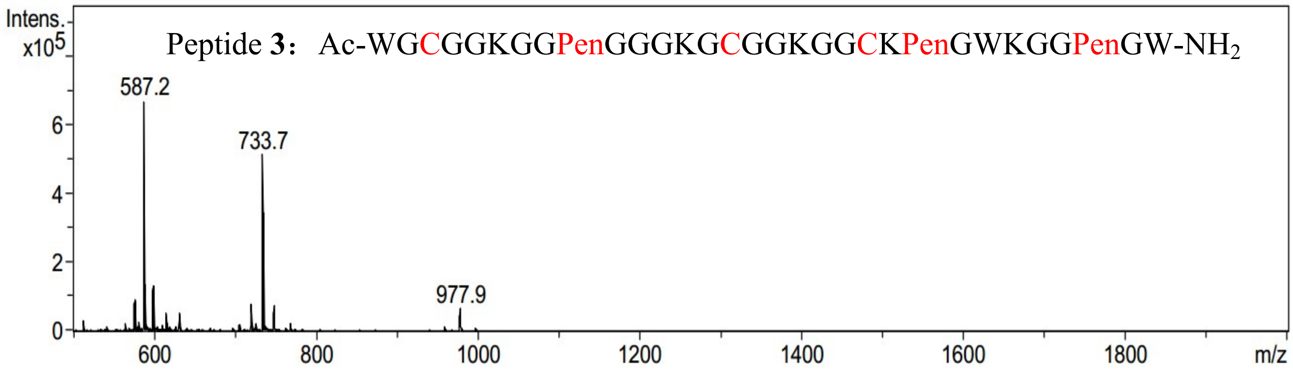


Mass spectrum of peptide **3**. Calcd (m/z): 2931.51; found 977.9 $(M+3H)^{3+}$, 733.7 $(M+4H)^{4+}$. 587.2 $(M+5H)^{5+}$.

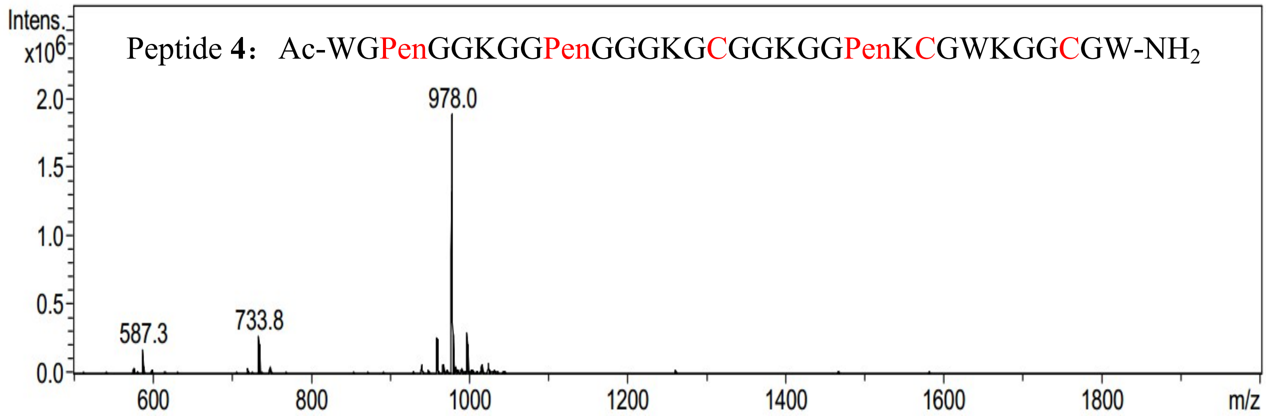


Mass spectrum of peptide **4**. Calcd (m/z): 2931.51; found 978.0 $(M+3H)^{3+}$, 733.8 $(M+4H)^{4+}$. 587.3 $(M+5H)^{5+}$.

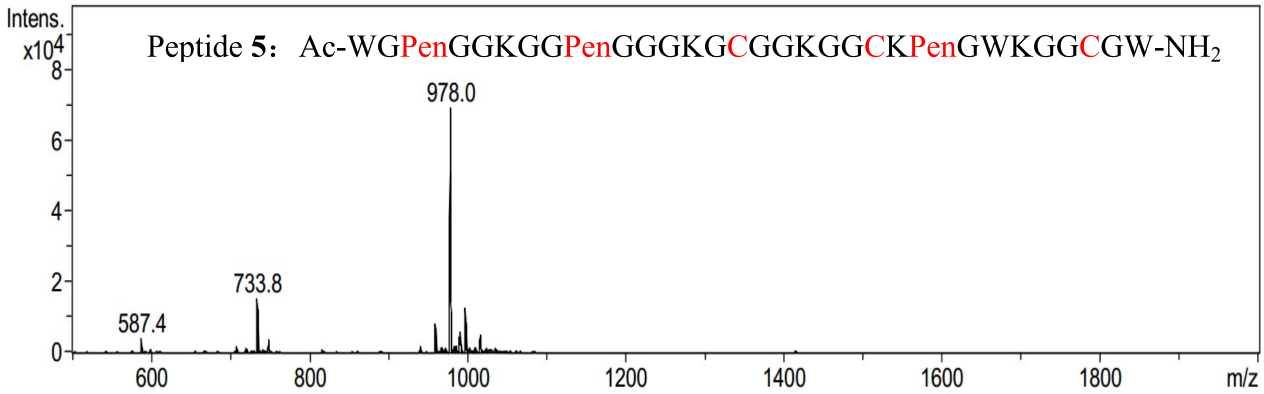


Mass spectrum of peptide **5**. Calcd (m/z): 2931.51; found 978.0 $(M+3H)^{3+}$, 733.8 $(M+4H)^{4+}$. 587.4 $(M+5H)^{5+}$.

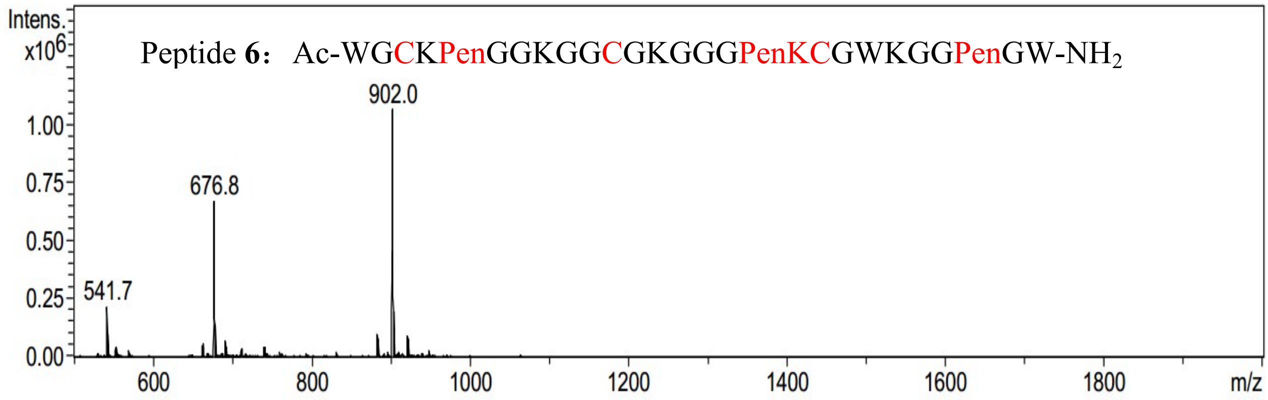


Mass spectrum of peptide **6**. Calcd (m/z): 2703.29; found 902.0 $(M+3H)^{3+}$, 676.8 $(M+4H)^{4+}$. 541.7 $(M+5H)^{5+}$.

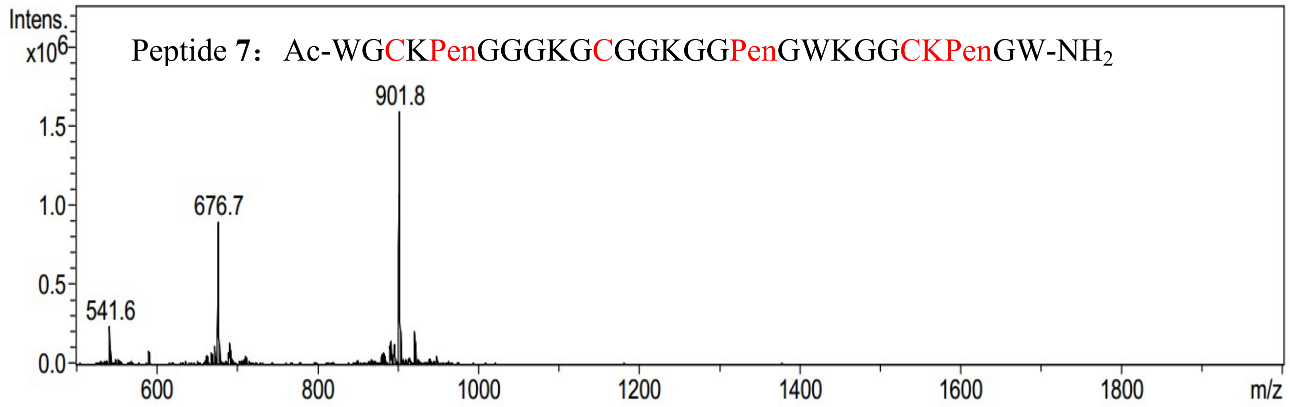


Mass spectrum of peptide **7**. Calcd (m/z): 2703.29; found 901.8 $(M+3H)^{3+}$, 676.7 $(M+4H)^{4+}$. 541.6 $(M+5H)^{5+}$.

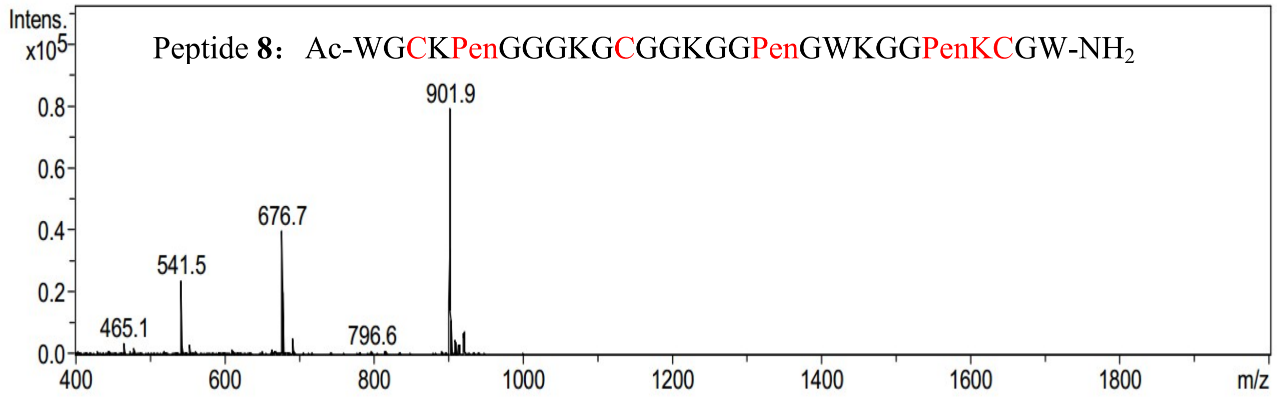


Mass spectrum of peptide **8**. Calcd (m/z): 2703.29; found 901.9 $(M+3H)^{3+}$, 676.7 $(M+4H)^{4+}$, 541.5 $(M+5H)^{5+}$.

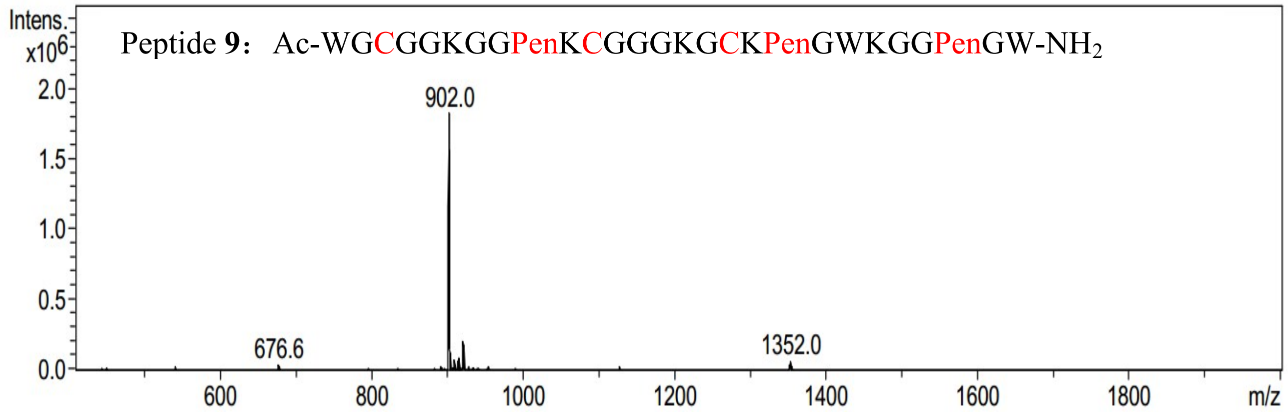


Mass spectrum of peptide **9**. Calcd (m/z): 2703.29; found 1352.0 $(M+2H)^{2+}$, 902.0 $(M+3H)^{3+}$, 676.7 $(M+4H)^{4+}$

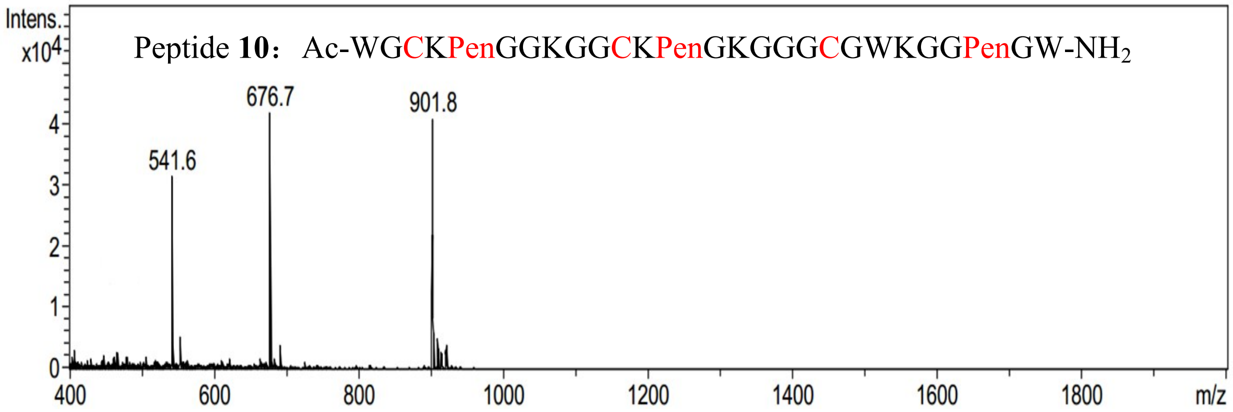


Mass spectrum of peptide **10**. Calcd (m/z): 2703.29; found 901.8 $(M+3H)^{3+}$, 676.7 $(M+4H)^{4+}$, 541.5 $(M+5H)^{5+}$.

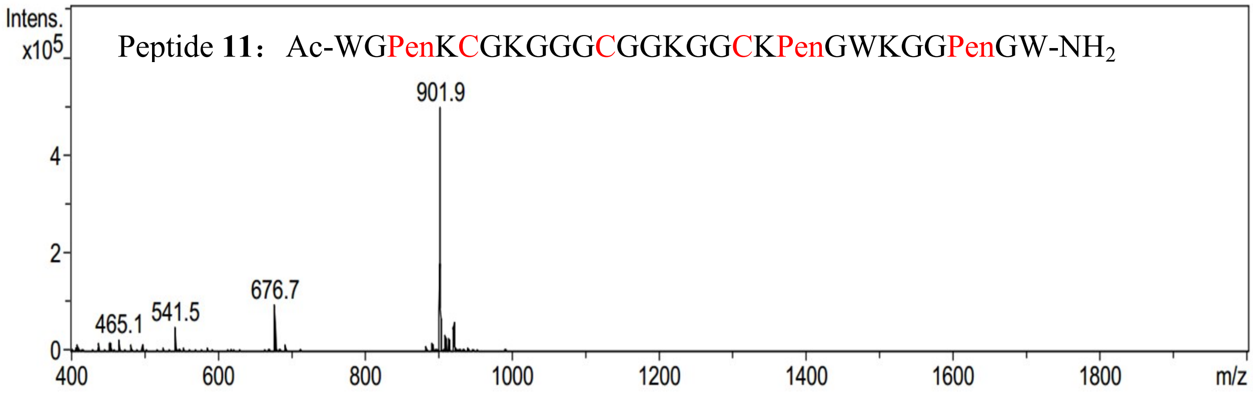


Mass spectrum of peptide **11**. Calcd (m/z): 2703.29; found 901.9 $(M+3H)^{3+}$, 676.7 $(M+4H)^{4+}$, 541.5 $(M+5H)^{5+}$.

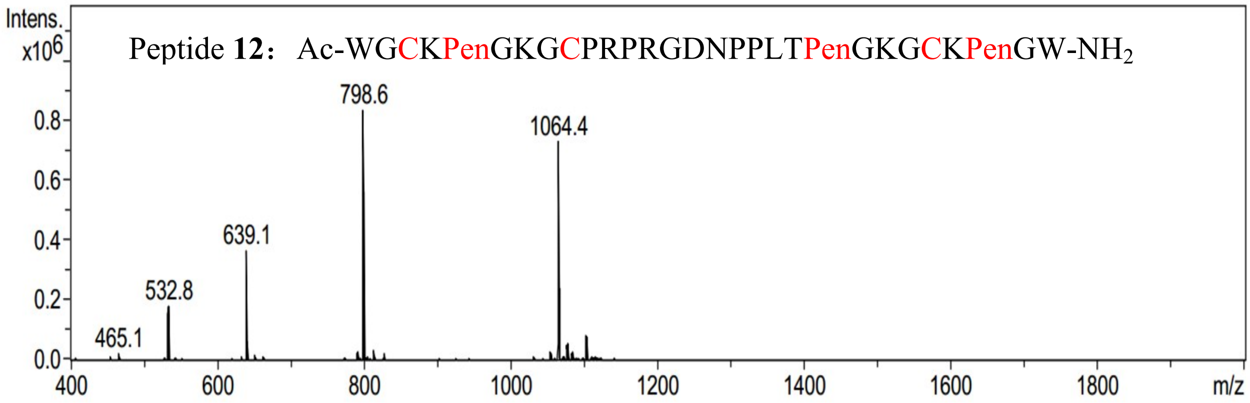


Mass spectrum of peptide **12**. Calcd (m/z): 3190.85; found 1064.4 $(M+3H)^{3+}$, 798.6$(M+4H)^{4+}$, 639.1 $(M+5H)^{5+}$, 532.8 $(M+6H)^{6+}$.

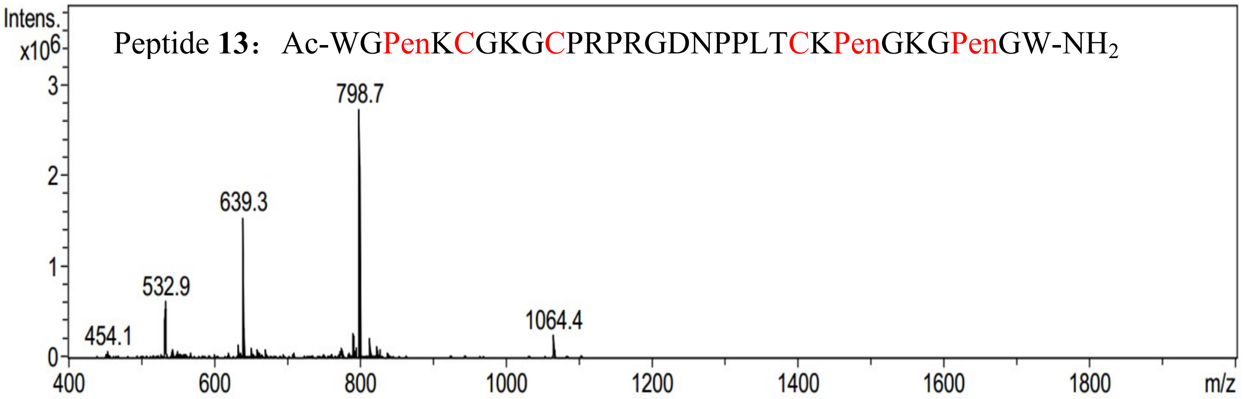


Mass spectrum of peptide **13**. Calcd (m/z): 3190.85; found 1064.4 $(M+3H)^{3+}$, 798.7$(M+4H)^{4+}$, 639.3 $(M+5H)^{5+}$, 532.9 $(M+6H)^{6+}$.

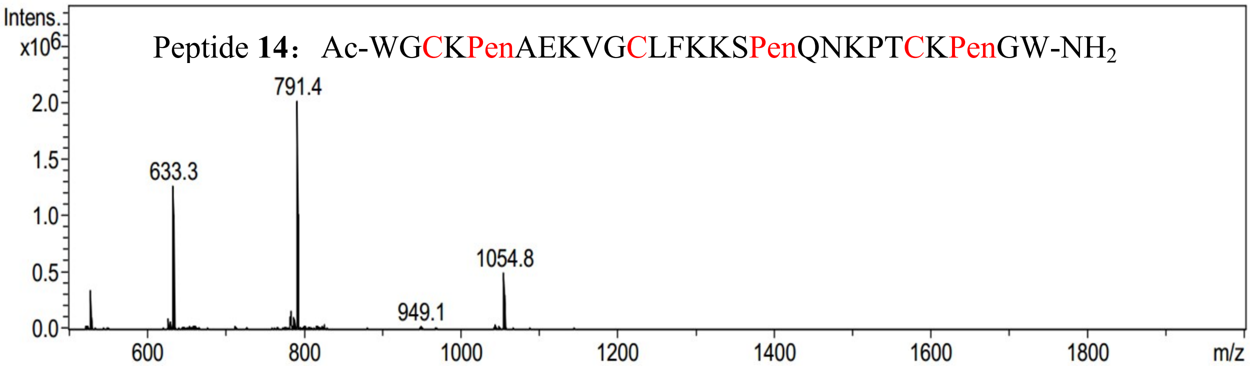


Mass spectrum of peptide **14**. Calcd (m/z): 3161.89; found 1054.8 $(M+3H)^{3+}$, 791.4 $(M+4H)^{4+}$, 633.3 $(M+5H)^{5+}$, 528.0 $(M+6H)^{6+}$.

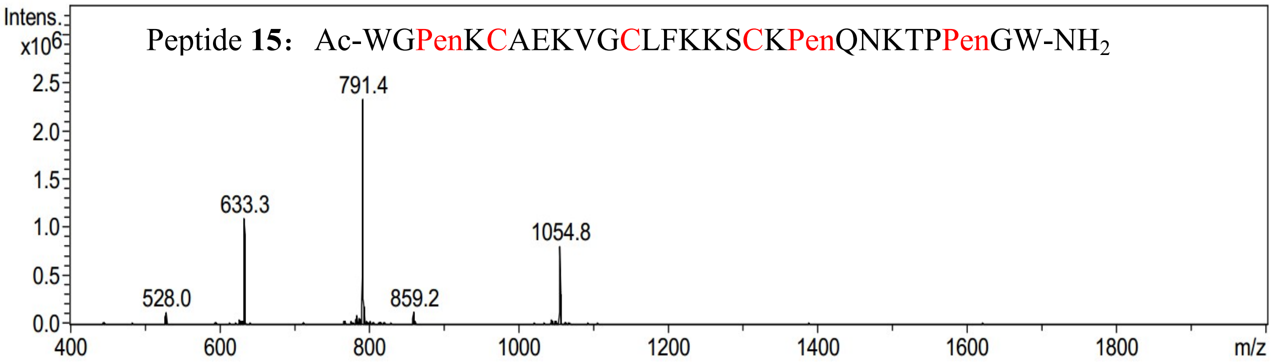


Mass spectrum of peptide **15**. Calcd (m/z): 3161.89; found 1054.8 $(M+3H)^{3+}$, 791.4 $(M+4H)^{4+}$, 633.3 $(M+5H)^{5+}$, 528.0 $(M+6H)^{6+}$.

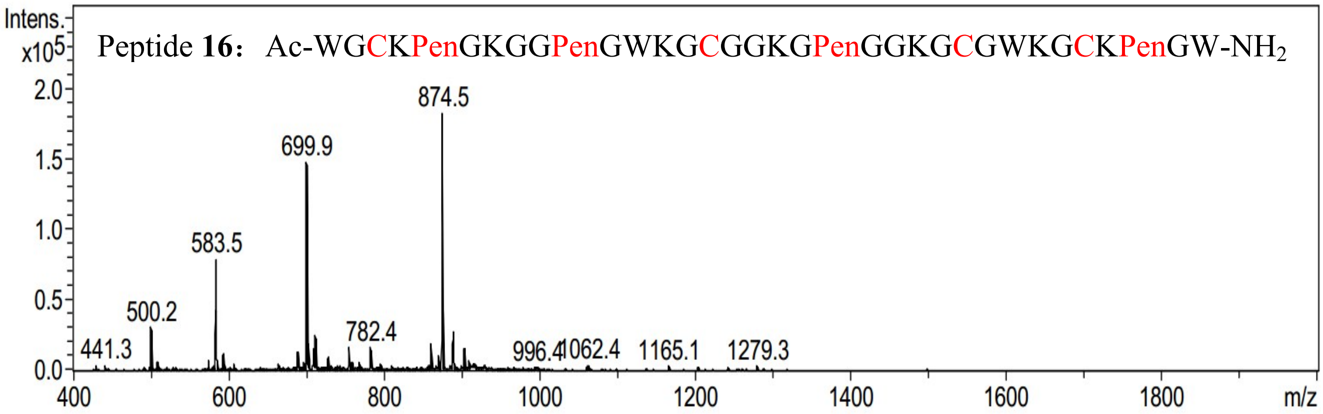


Mass spectrum of peptide **16**. Calcd (m/z): 3494.30; found 1165.1 $(M+3H)^{3+}$, 874.5 $(M+4H)^{4+}$, 699.9 $(M+5H)^{5+}$, 583.5 $(M+6H)^{6+}$, 500.2 $(M+7H)^{7+}$.
